# Discovery of Plant-Derived Natural Compounds as Novel GABA Aminotransferase Inhibitors: Structure-Based Discovery, Experimental Validation, and Molecular Dynamics Analysis

**DOI:** 10.3390/ph19020307

**Published:** 2026-02-12

**Authors:** Jinyoung Park, Muhammad Yasir, Eun-Taek Han, Won Sun Park, Jin-Hee Han, Jongseon Choe, Wanjoo Chun

**Affiliations:** 1 Department of Pharmacology, School of Medicine, Kangwon National University, Chuncheon 24341, Republic of Korea; jinyoung0326@kangwon.ac.kr (J.P.); yasir.khokhar1999@gmail.com (M.Y.); 2 Department of Medical Environmental Biology and Tropical Medicine, School of Medicine, Kangwon National University, Chuncheon 24341, Republic of Korea; ethan@kangwon.ac.kr (E.-T.H.); han.han@kangwon.ac.kr (J.-H.H.); 3 Department of Physiology, School of Medicine, Kangwon National University, Chuncheon 24341, Republic of Korea; parkws@kangwon.ac.kr; 4 Department of Microbiology and Immunology, School of Medicine, Kangwon National University, Chuncheon 24341, Republic of Korea; jchoe@kangwon.ac.kr

**Keywords:** GABA, GABA aminotransferase inhibitor, epilepsy, molecular docking, molecular dynamics simulation, quercetin, salvianolic acid A, scutellarein

## Abstract

**Background/Objectives:** γ-Aminobutyric acid aminotransferase (GABA-AT) is a key enzyme responsible for GABA catabolism and represents a validated therapeutic target for epilepsy. Although existing GABA-AT inhibitors such as vigabatrin are clinically effective, their long-term use is limited by safety concerns, highlighting the need for alternative inhibitors with improved profiles. In this study, we employed an integrated natural product-oriented discovery strategy to identify novel GABA-AT inhibitors from plant-derived compounds. **Methods**: A library of 1006 plant-derived compounds collected from seven medicinal plants traditionally associated with sedative or anxiolytic effects was subjected to primary virtual screening using GNINA. Top-ranked candidates were further refined through secondary precision docking using aglycone forms to account for biologically relevant metabolic conversion. Detailed interaction analyses and molecular dynamics simulations were performed to assess binding stability and energetic favorability. **Results:** Based on computational prioritization, quercetin, salvianolic acid A, and scutellarein were selected for experimental validation. Cell-based GABA-AT activity assays in HepG2 cells demonstrated that quercetin and salvianolic acid A significantly inhibited intracellular GABA-AT activity, exhibiting comparable or greater efficacy than vigabatrin, while scutellarein showed moderate inhibition. The observed cellular inhibitory effects were consistent with predicted binding modes and dynamic stability observed in in silico analyses. **Conclusions:** Collectively, this study highlights the utility of an aglycone-focused, structure-based screening strategy for natural product drug discovery and identifies plant-derived aglycones as promising GABA-AT inhibitor candidates for further pharmacological development.

## 1. Introduction

γ-Aminobutyric acid (GABA) is the principal inhibitory neurotransmitter in the central nervous system and plays a critical role in maintaining the balance between neuronal excitation and inhibition [1,2,3,4]. Dysregulation of GABAergic signaling has been closely associated with the pathophysiology of various neurological disorders, including epilepsy, anxiety disorders, and sleep disturbances [5,6,7]. The fundamental role of GABA_A_ receptor-mediated neurotransmission in epilepsy pathogenesis and its therapeutic implications have been extensively characterized [8]. Among these, epilepsy—a neurological disorder characterized by recurrent seizures resulting from excessive neuronal excitability—represents a particularly relevant therapeutic context for GABA-AT modulation. In this context, GABA aminotransferase (GABA-AT), the key enzyme responsible for GABA degradation, has emerged as an important therapeutic target for epilepsy treatment [9,10]. Inhibition of GABA-AT leads to elevated intracellular GABA levels, thereby enhancing inhibitory neurotransmission and suppressing seizure activity. Accordingly, GABA-AT inhibitors such as vigabatrin have been clinically employed for the management of refractory epilepsy [11,12,13,14,15]. Despite the established efficacy of GABAergic pharmacotherapy, the clinical use of existing GABA-AT inhibitors is limited by adverse effects and safety concerns associated with long-term administration [16], underscoring the need for the development of novel and safer GABA-AT-targeting agents with improved therapeutic profiles [9,17,18,19,20].

Medicinal plants traditionally used for sedative, anxiolytic, and anticonvulsant purposes represent a promising source of bioactive compounds capable of modulating GABAergic pathways. Natural products exhibit high structural diversity and favorable biocompatibility, offering advantages over synthetic compounds for discovering new drug candidates with improved safety profiles and novel mechanisms of action [21,22,23]. Therefore, the identification of GABA-AT inhibitors from phytochemicals derived from traditional medicinal plants constitutes a rational and strategic approach for antiepileptic drug discovery.

Several medicinal plants traditionally used for their calming, anxiolytic, or sedative properties are of particular interest in the search for natural GABA-AT inhibitors. *Valeriana officinalis* (valerian) is widely consumed for sleep disturbances and has been associated with modulation of GABAergic signaling [21]. *Passiflora incarnata* (passionflower) contains flavonoids that have been linked to anxiolytic effects [22], while *Piper methysticum* (kava) has been reported to influence GABA-related neurotransmission [21]. In addition, *Scutellaria baicalensis* (Baikal skullcap) and *Melissa officinalis* (lemon balm) are rich in polyphenolic constituents that may enhance GABA-mediated inhibitory tone through enzymatic modulation or receptor-level interactions [21,22]. Likewise, *Humulus lupulus* (hops) and *Magnolia officinalis* (magnolia bark) harbor bioactive compounds with central nervous system depressant properties, suggesting potential involvement in the regulation of GABA metabolism [24,25,26,27]. Collectively, these medicinal plants provide a biologically relevant and chemically diverse source of plant-derived compounds for discovering novel GABA-AT inhibitors.

**Figure 1:**
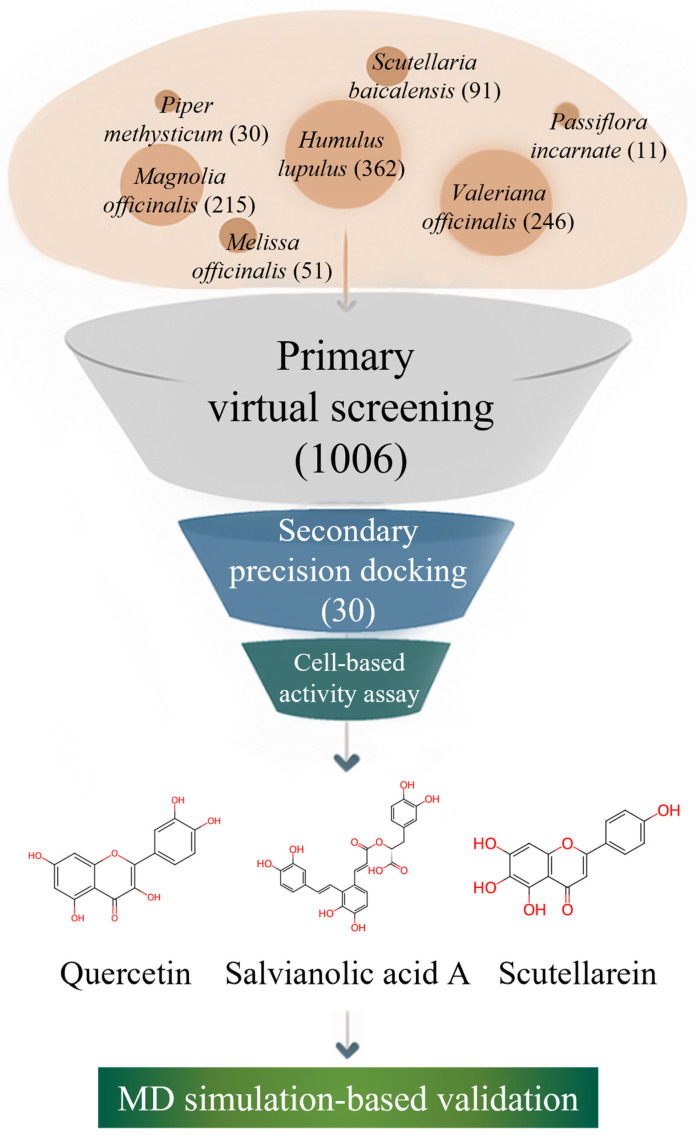
Workflow of the integrated natural product-based discovery of GABA-AT inhibitors, combining primary virtual screening, secondary structure-based docking, cell-based validation, and molecular dynamics simulation.

As illustrated in Figure 1, we systematically investigated plant-derived compounds reported in medicinal plants with documented neuro-calming effects to identify potential GABA-AT inhibitors. An initial high-throughput virtual screening was conducted using GNINA-based molecular docking to evaluate the binding affinity of a large phytochemical library toward GABA-AT. Compounds exhibiting high docking scores were subsequently subjected to more detailed three-dimensional molecular docking analyses using Discovery Studio 2021 to refine protein–ligand interaction profiles. This multi-stage computational strategy is consistent with recent drug discovery studies in which structure-based docking and molecular dynamics simulations were successfully combined with experimental validation to identify functionally relevant modulators of pharmacological targets [28]. The inhibitory effects of selected candidate compounds were then experimentally validated using a cell-based GABA-AT activity assay in HepG2 cells. Finally, molecular dynamics (MD) simulations were performed to further assess the stability and dynamic behavior of the GABA-AT–ligand complexes. Through this integrated computational and experimental approach, this study aims to identify and validate plant–derived GABA-AT inhibitors as potential therapeutic candidates for epilepsy.

## 2. Results and Discussion

### 2.1. Collection of Plant-Derived Compound Libraries from Medicinal Plants

Seven medicinal plants traditionally associated with neuro-calming, anxiolytic, or central nervous system (CNS)-depressant effects were selected as sources for constructing a plant-derived compound library. These plants—*Valeriana officinalis*, *Passiflora incarnata*, *Piper methysticum*, *Scutellaria baicalensis*, *Melissa officinalis*, *Humulus lupulus*, and *Magnolia officinalis*—have been widely used in traditional medicine systems for the management of sleep disorders, anxiety, or stress-related conditions [22,29,30]. Importantly, previous pharmacological and phytochemical studies have suggested that these species contain bioactive constituents capable of modulating GABAergic neurotransmission, either through receptor-level interactions or enzymatic regulation, making them particularly relevant for the identification of potential GABA-AT inhibitors [21].

Using the KNApSAcK Family Database, a systematic survey of reported plant-derived compounds was conducted for each plant species. As a result, a total of 1006 unique compounds were identified across the seven plants. Specifically, 246 compounds were retrieved from *Valeriana officinalis*, 11 compounds from *Passiflora incarnata*, and 30 compounds from *Piper methysticum*. In addition, *Scutellaria baicalensis* yielded 91 compounds, *Melissa officinalis* yielded 51 compounds, *Humulus lupulus* yielded the largest number with 362 compounds, and *Magnolia officinalis* contributed 215 compounds to the library.

Although the size of the compound libraries differed markedly among species, this heterogeneity was intentionally preserved to avoid biasing the subsequent computational screening toward plants with fewer but more intensively studied constituents. Instead, the inclusion of chemically diverse plant-derived compounds allowed for an unbiased evaluation of how structural features, rather than source plant abundance, influence predicted binding affinity toward GABA-AT. This strategy also enabled later comparative analyses to determine whether high-affinity candidates are proportional to overall phytochemical diversity or driven by specific molecular scaffolds. Collectively, the rational selection of these seven medicinal plants and the comprehensive compilation of their reported plant-derived compounds established a robust and biologically relevant foundation for subsequent primary virtual screening and secondary precision docking aimed at identifying novel natural GABA-AT inhibitor candidates.

### 2.2. Primary Virtual Screening of Plant-Derived Compounds Against GABA-AT

To identify potential GABA-AT inhibitors from the compiled plant-derived compound library, a structure-based primary virtual screening was conducted using GNINA. The three-dimensional structure of human GABA-AT was obtained from the Protein Data Bank, and PDB ID: 4Y0H was selected for all docking simulations. GNINA was employed as a high-throughput docking platform, integrating deep learning-based scoring functions to efficiently estimate protein-ligand binding affinities [31,32].

All 1006 plant-derived compounds were docked into the defined catalytic pocket of GABA-AT, and their predicted binding affinities were ranked according to GNINA docking scores. All predicted docking scores for the complete set of 1006 plant-derived compounds obtained from the primary virtual screening are provided in Supplementary Table S1. The top 30 compounds exhibiting the most favorable docking scores were subsequently selected as putative GABA-AT inhibitor candidates for further investigation, and their docking results are summarized in Table 1.

To facilitate intuitive interpretation of the relationship between medicinal plants and their high-ranking GABA-AT binding constituents, the top 30 phytochemicals were visualized using a Sankey diagram. In this diagram, each phytochemical is connected to GABA-AT, illustrating its predicted interaction with the target enzyme. Compounds are ordered according to their GNINA docking score ranking, with higher-ranked candidates positioned preferentially.

**Figure 2:**
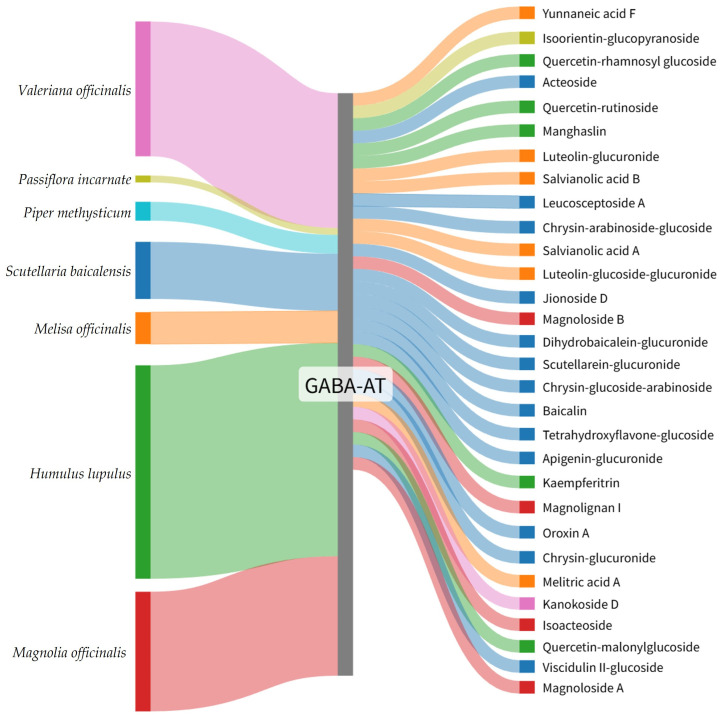
Sankey diagram illustrating the plant origin of the top 30 GABA-AT binding plant-derived compounds identified by GNINA-based molecular docking. Colors represent the source plants species, and compounds are arranged according to their GNINA docking score ranking.

The Sankey diagram further highlights the plant origin of each compound through color coding, where phytochemicals derived from the same medicinal plant are represented using identical colors (Figure 2). This visualization demonstrated that the distribution of top-ranked GABA-AT binding candidates did not scale proportionally with the total number of reported phytochemicals for each medicinal plant. Specifically, only 5 compounds from *Humulus lupulus* were included among the top 30 candidates despite the presence of 362 reported constituents. Similarly, *Magnolia officinalis* and *Valeriana officinalis* contributed 4 and 1 candidates out of 215 and 246 reported compounds, respectively. In contrast, plants with comparatively fewer reported constituents, such as *Scutellaria baicalensis* and *Melissa officinalis*, yielded a higher proportion of top-ranked candidates, with 13 out of 91 and 6 out of 51 compounds identified, respectively.

These findings indicate that GABA-AT binding affinity is primarily determined by the structural compatibility of individual plant-derived compounds with the enzyme’s active site rather than by the overall chemical diversity of their source plants. Structure-based virtual screening therefore represents an effective strategy for prioritizing functionally relevant natural compounds, enabling efficient selection of high-affinity candidates for subsequent precision docking, experimental validation, and molecular dynamics simulations.

### 2.3. Secondary Precision Docking and Binding Pose Validation

Secondary precision docking was performed to refine and validate the binding affinities of selected plant-derived compounds toward GABA-AT using the CDOCKER algorithm implemented in Discovery Studio. Based on the results of the primary virtual screening using GNINA, the top 30 plant-derived compounds were initially selected. However, to ensure structural consistency and biological relevance, glycosylated forms were excluded, as such moieties are frequently hydrolyzed during gastrointestinal absorption, yielding aglycone forms as the predominant bioactive species [33,34,35]. Accordingly, only aglycone structures were retained, resulting in a final set of 25 plant-derived aglycones subjected to secondary precision docking. The reference inhibitor vigabatrin was included for comparative evaluation.

Docking results revealed a broad distribution of binding affinities among the evaluated plant-derived aglycones, enabling effective stratification based on predicted interaction strength within the GABA-AT active site. Plant-derived aglycones were ranked primarily according to their CDOCKER Energy values, with more negative values indicating more favorable ligand-receptor interactions [36].

Among all tested plant-derived aglycones, salvianolic acid A exhibited the most favorable binding profile, displaying the lowest CDOCKER Energy (−61.1521 kcal/mol) along with a strong CDOCKER Interaction Energy (−69.1719 kcal/mol). This binding profile suggests excellent spatial complementarity and optimized noncovalent interactions within the active site. Other high-ranking plant-derived aglycones included quercetin (−57.4877 kcal/mol), melitric acid A (−55.7288 kcal/mol), and scutellarein (−54.8822 kcal/mol), all of which demonstrated consistently strong docking energies. These results highlight the contribution of polyphenolic aglycone scaffolds, which provide multiple hydrogen-bond donors and acceptors as well as extended aromatic systems favorable for stabilizing interactions within the enzyme pocket.

Several additional plant-derived aglycones, including tetrahydroxyflavone (−50.4093 kcal/mol), baicalin (−46.9267 kcal/mol), salvianolic acid B (−46.5809 kcal/mol), and apigenin (−45.8413 kcal/mol), showed moderate yet stable binding affinities (Table 2). While these plant-derived aglycones maintained effective engagement with the active site, their comparatively reduced docking scores may reflect differences in functional group orientation, molecular flexibility, or interaction geometry relative to the top-ranked ligands.

The reference compound vigabatrin exhibited a CDOCKER Energy of −42.0196 kcal/mol, placing it within the moderate-affinity range. Notably, several plant-derived aglycones-particularly salvianolic acid A, quercetin, melitric acid A, and scutellarein-demonstrated substantially more favorable docking energies than vigabatrin, indicating superior predicted binding affinity toward GABA-AT. In contrast, plant-derived aglycones such as dihydrobaicalein (−35.1200 kcal/mol) and luteolin (−35.0814 kcal/mol) displayed relatively weaker binding, although they retained the capacity to interact with the active site.

Collectively, these findings underscore the importance of excluding bulky glycosidic moieties and focusing on aglycone forms during secondary precision docking. The enhanced binding efficiency observed for plant-derived aglycones is likely attributable to improved steric fit and optimized interaction networks within the GABA-AT active site. The high-ranking plant-derived aglycones identified through this strategy were therefore selected as promising candidates for subsequent molecular dynamics simulations and experimental validation.

### 2.4. Molecular Docking Interaction Analysis

The comparative interaction analysis of the 4 top-ranked plant-derived aglycones provides important mechanistic insight into the structural determinants governing their superior docking performance relative to the reference compound, vigabatrin. A common and striking feature across all high-affinity ligands is their consistent salt bridge interaction with the binding pocket amino acid residue.

**Figure 3:**
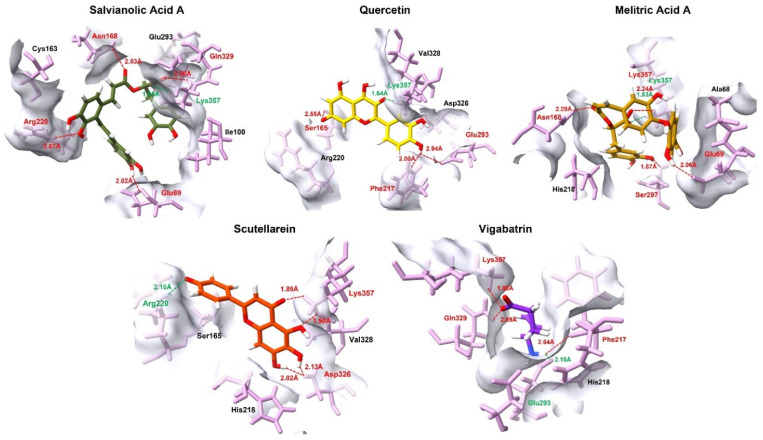
The 3D interaction profile of the top 4 plant-derived aglycones against the GABA-AT in comparison with the reference compound, vigabatrin. Ligands are distinguished by various colors, while interacting amino acid residues are highlighted in pink. Specific intermolecular forces are color-coded for clarity: red represents hydrogen bonds, and green denotes salt bridges.

Salvianolic acid A exhibited the most extensive interaction profile, engaging five residues (Lys357, Glu69, Asn168, Gln329, and Arg220) through a combination of salt bridges and hydrogen bonds. Simultaneous interaction with both positively charged (Lys357, Arg220) and negatively charged (Glu69) residues indicates optimal charge distribution and spatial adaptability of this ligand within the binding pocket. This multivalent interaction pattern likely accounts for its strongest docking affinity, as the ligand is stabilized by a cooperative network of short-range interactions that reduce conformational freedom and enhance residence time, rather than by a single dominant contract. Quercetin, following salvianolic acid A, also demonstrated a highly favorable binding mode characterized by the shortest salt bridge distance (1.64 Å) with Lys357 (Figure 3). Its additional contacts with Glu293, Ser165, and Phe217 suggest a balanced interaction scheme. Compared with salvianolic acid A, quercetin appears to rely more on precise orientation and aromatic complementarity rather than interaction multiplicity, which may explain its slightly reduced but still strong docking score.

Melitric acid A displayed a distinctive binding pattern through the formation of dual interactions with Lys357, (salt bridge and hydrogen bond). This dual electrostatic and hydrogen bond anchoring, coupled with other short hydrogen-bond distances to Glu69, Ser297, and Asn168, suggests highly strong local stabilization. The redundancy of key interactions implies a reduced likelihood of binding disruption due to minor conformational fluctuations, positioning melitric acid A as a particularly robust binder despite having fewer interacting residues than salvianolic acid A. Scutellarein showed a comparatively simpler but highly focused interaction profile, dominated by a salt bridge with Arg220 and additional stabilizing hydrogen bonding contacts with Lys357 and Asp326. This pattern indicates that scutellarein achieves effective binding primarily through focused interactions rather than through interaction diversity.

When contrasted with these plant-derived compounds, vigabatrin displayed a comparable interaction footprint involving Glu293, Phe217, Lys357, and Gln329. Notably, vigabatrin formed a salt bridge with Glu293, in contrast to the majority of screened compounds, which preferentially established salt bridge interactions with Lys357. Nevertheless, vigabatrin maintained a strong hydrogen bond with Lys357 at a short distance (1.88 Å), indicating partial engagement of this critical anchoring residue (Table 3). However, the absence of a dense or cooperative interaction network likely contributes to its comparatively lower docking affinity. In addition to electrostatic and hydrogen-bonding interactions, hydrophobic contributions further stabilized ligand binding within the pocket. Aromatic and aliphatic contacts with residues such as Phe217, Val328, Ala68, and Ile100 were observed across the top-ranked aglycones (Supplementary Figure S2), promoting favorable van der Walls packing and shape complementarity. These hydrophobic interactions likely reduce solvent exposure and contribute to binding persistence, complementing the dominant salt bridges and hydrogen bonds.

Collectively, these findings support the hypothesis that high-affinity binding within this active site is driven by a combination of strong electrostatic anchoring at Lys357 and complementary secondary interactions with surrounding polar and charged residues. Plant-derived aglycones such as salvianolic acid A and quercetin, which maximize both interaction multiplicity and electrostatic strength, appear particularly well suited for stable target engagement. This comparative interaction behavior provides a structural rationale for the superior docking performance of the top plant-derived compounds and supports their prioritization for further experimental validation.

### 2.5. Cell-Based Evaluation of GABA-AT Inhibitory Activity

The inhibitory effects of quercetin, salvianolic acid A, and scutellarein on GABA-AT activity were evaluated using a cell-based assay in HepG2 cells. The plant-derived aglycones were directly applied to cells to assess their effects on intracellular GABA-AT activity under physiologically relevant conditions. HepG2 cells, a human hepatocellular carcinoma cell line, have been widely used as an in vitro model for studies of GABA metabolism and enzyme modulation due to their hepatic origin and stable metabolic activity, and were therefore employed to evaluate intracellular GABA-AT inhibition following plant-derived aglycones treatment.

Prior to the GABA-AT inhibitory activity assay, cytotoxicity of the plant-derived aglycones was evaluated in HepG2 cells. All tested aglycones maintained cell viability above 97% at concentrations up to 100 μM after 2 days treatment, confirming the absence of cytotoxic effects under assay conditions (Supplementary Figure S1). Intracellular GABA-AT activity was quantified using a resazurin-based coupled assay, in which enzymatic activity is reflected by fluorescence intensity. Following plant-derived aglycones treatment, cell lysates were subjected to the coupled reaction, and changes in resazurin reduction were used as a surrogate measure of GABA-AT activity. Because compounds were removed by medium exchange and washout prior to cell lysis, the assay design minimized direct redox or optical interference from extracellular polyphenols in the coupled reaction system. Nevertheless, the observed effects are interpreted as intracellular GABA-AT-associated activity modulation, as indirect contributions arising from intracellular retention or treatment-induced metabolic alterations cannot be fully excluded. Because the cell-based assay reflects intracellular GABA-AT-associated activity rather than isolated enzyme kinetics, the present data were interpreted qualitatively, and precise IC_50_ values were not determined in this study. Vigabatrin, a clinically established irreversible GABA-AT inhibitor, was included as a functional reference to benchmark intracellular inhibitory efficacy in the cell-based assay.

**Figure 4:**
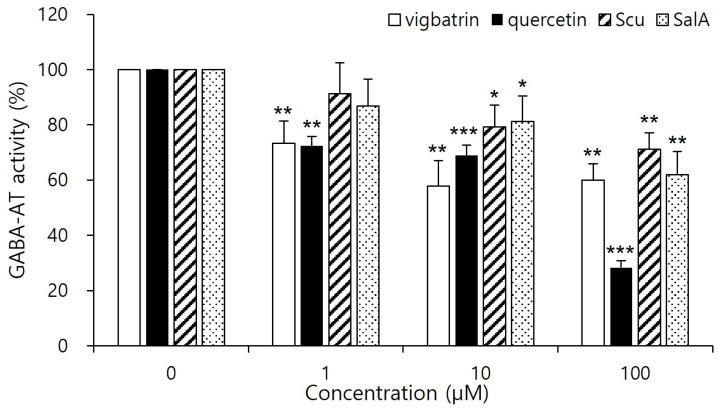
Cell-based inhibitory effects of plant-derived aglycones on GABA-AT activity in HepG2 cells. Cells were treated with quercetin, salvianolic acid A, scutellarein, or vigabatrin at the indicated concentration (1, 10, 100 μM) for 2 days, followed by cell lysis. Intracellular GABA-AT activity was quantified using a resazurin-based coupled assay. Vigabatrin was used as a reference inhibitor. Data are presented as mean ± SD of independent experiments (*n* = 4). Statistical significance was determined relative to the control group (* *p* < 0.05, ** *p* < 0.01, *** *p* < 0.001).

As shown in Figure 4, quercetin significantly reduced GABA-AT activity at a concentration of 100 μM, exhibiting a stronger inhibitory effect than vigabatrin at the same concentration. Salvianolic acid A also showed pronounced inhibition of GABA-AT activity, exceeding that of vigabatrin, whereas scutellarein demonstrated inhibitory activity that was comparable to or weaker than that of vigabatrin. These results demonstrate that quercetin and salvianolic acid A are capable of effectively inhibiting GABA-AT activity in a cellular context, thereby confirming that their inhibitory potential is retained under intracellular conditions rather than being limited to in silico predictions. The superior inhibitory efficacy of quercetin and salvianolic acid A is consistent with the molecular docking and interaction analyses, which revealed strong electrostatic anchoring and extensive hydrogen-bond networks with key active-site residues of GABA-AT. This concordance between computational binding affinity and cell-based functional inhibition supports the biological relevance of the predicted binding modes. In contrast, the comparatively weaker inhibition observed for scutellarein aligns with its more limited interaction profile identified in docking studies, suggesting that reduced interaction multiplicity may translate into lower intracellular inhibitory potency. Importantly, both quercetin and salvianolic acid A exhibited inhibitory effects that were comparable to or greater than those of vigabatrin at the same concentration, despite the latter being an irreversible inhibitor. Notably, no visible precipitation, turbidity, or cytotoxicity was observed at this concentration, suggesting that the observed inhibitory effects are unlikely to arise from compound aggregation or nonspecific physicochemical interference. Nevertheless, because the present evaluation was conducted using a cell-based assay coupled to a downstream enzymatic readout, the observed effects should be interpreted as reflecting intracellular GABA-AT–associated activity modulation rather than definitive evidence of direct enzyme inhibition at the molecular level [37]. This observation suggests that these plant-derived aglycones may inhibit GABA-AT through alternative, potentially reversible mechanisms, which could offer advantages in safety and tunability of enzyme modulation.

Overall, the cell-based inhibition data validate the virtual screening and docking-based prioritization strategy employed in this study and highlight quercetin and salvianolic acid A as promising GABA-AT inhibitor candidates for further mechanistic investigation and pharmacological evaluation. The cellular findings obtained using HepG2 cells should be interpreted as evidence of intracellular GABA-AT-associated activity modulation rather than as direct evidence of CNS-specific GABAergic effects.

### 2.6. Molecular Dynamics Simulation Analysis

To further evaluate the stability of the top three plant-derived aglycones against GABA-AT, 100 ns molecular dynamics (MD) simulations were performed in triplicate using GROMACS. This analysis provided a dynamic assessment to confirm the potential of these compounds as effective inhibitors of GABA-AT.

### 2.7. Root Mean Square Deviation

The comparative RMSD-based molecular dynamics analysis provides quantitative insight into the conformational stability of the top three plant-derived aglycones, quercetin, salvianolic acid A, and scutellarein, in comparison with the reference compound, vigabatrin. RMSD values were evaluated across three independent simulation replicates (R1–R3).

Quercetin demonstrated consistently low RMSD values across all replicates, with an overall average RMSD of 0.30. The mean RMSD values ranged from 0.25 ± 0.07 to 0.33 ± 0.05, with relatively narrow standard deviations, indicating limited structural fluctuations and stable binding throughout the simulations. Among the three replicates, R3 exhibited the highest stability score, while R2 showed only a marginally lower stability. The absence of large RMSD excursions (maximum ≤ 0.65) suggests that quercetin maintains a well-constrained orientation. Salvianolic acid A displayed slightly higher overall RMSD values than quercetin, with an overall average of 0.35; however, it exhibited the highest stability among the plant-derived aglycones. Replicates R1 and R3 showed particularly low RMSD fluctuations (0.30 ± 0.04 and 0.29 ± 0.04, respectively), accompanied by excellent stability scores. Although R2 displayed increased mobility (mean RMSD 0.47 ± 0.08), the consistently low minimum RMSD values and strong stabilization in R3 suggest that salvianolic acid A achieves highly stable binding once an optimal interaction geometry is established.

**Figure 5:**
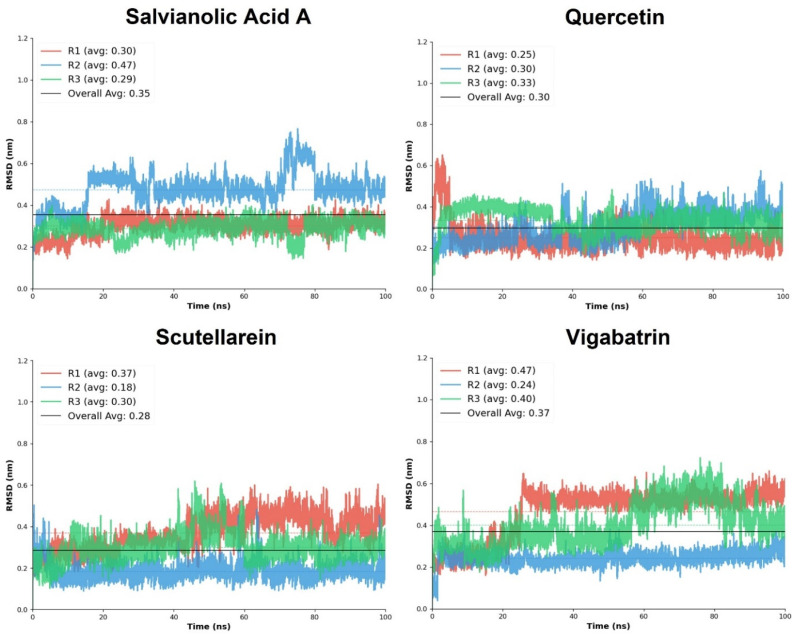
The RMSD replicates analysis of the plant-derived aglycones in comparison with the reference compound, vigabatrin.

Scutellarein exhibited the lowest overall average RMSD (0.28) among the tested plant-derived aglycones, indicating high structural stability during the simulations. Notably, replicate R2 showed exceptional stability, with a mean RMSD of 0.18 ± 0.04 and a high stability score. Although R1 showed comparatively higher fluctuations (0.37 ± 0.08), the overall RMSD distribution remained narrow, and the maximum RMSD values did not exceed 0.62 (Figure 5). These results suggest that scutellarein can adopt a tightly constrained binding mode, likely driven by its focused interaction pattern and strong electrostatic anchoring observed during docking. In contrast, the reference compound vigabatrin displayed 0.37 overall average RMSD, reflecting comparatively reduced conformational stability of the ligand-protein complex as compared to the top 3 plant-derived aglycones. While replicate R2 achieved high stability (mean RMSD 0.24 ± 0.04; stability score 0.966), the other replicates (R1 and R3) exhibited moderate fluctuations, which lowered stability scores. The wider RMSD range (maximum up to 0.72) indicates greater positional variability of vigabatrin within the binding pocket, likely due to its simpler molecular scaffold.

Overall, the comparative RMSD analysis clearly demonstrates that quercetin, salvianolic acid A, and scutellarein form dynamically stable complexes. Among the plant-derived aglycones, scutellarein showed the lowest average RMSD, quercetin exhibited the most consistent stability across replicates, and salvianolic acid A achieved the highest replicate-level stability scores. These findings complement the docking interaction analysis and support the hypothesis that enhanced electrostatic anchoring and cooperative hydrogen-bond networks contribute to improved dynamic stability.

### 2.8. Hydrogen Bond Plot Analysis

Hydrogen bond (H-bond) analysis was performed over the 100 ns molecular dynamics simulation to evaluate the stability and persistence of ligand-protein interactions, using vigabatrin as the reference compound. The Intermolecular hydrogen bonds with donor–acceptor distances ≤ 0.35 nm were classified as potential hydrogen bonds, whereas the detected hydrogen bonds during the 100 ns MD trajectory were kept as actual hydrogen bonds. This analysis provides insight into the dynamic binding behavior of the plant-derived aglycones and their ability to maintain key stabilizing interactions within the binding pocket under physiological simulation conditions.

**Figure 6:**
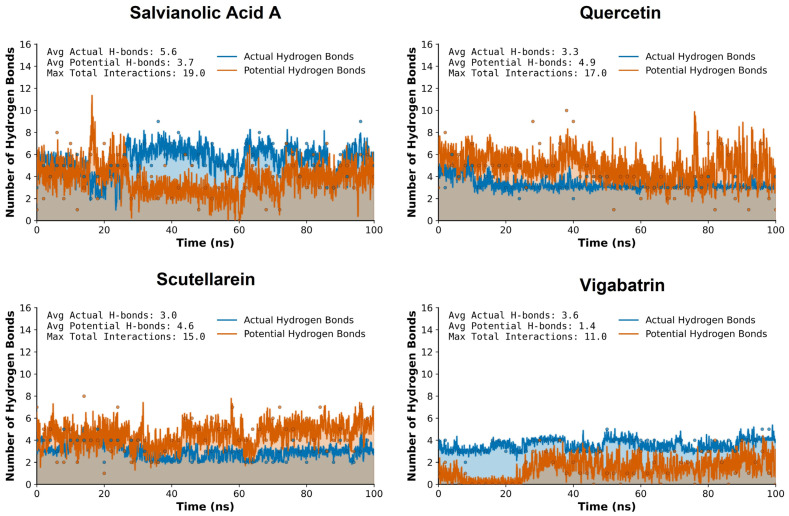
The hydrogen bond plot analysis of the plant-derived aglycones (salvianolic acid A, quercetin, and sctellarein) during the 100 ns MD trajectory of run 1.

Vigabatrin, the reference inhibitor, displayed a moderate and relatively stable hydrogen bonding pattern throughout the simulation trajectory. The H-bond profile indicates intermittent formation and disruption of hydrogen bonds. While vigabatrin maintained acceptable interaction stability, the frequency and persistence of hydrogen bonds below the 0.35 nm threshold were comparatively modest (Figure 6).

In comparison, quercetin showed improved hydrogen bonding characteristics relative to the reference compound. Its multiple hydroxyl functionalities enabled repeated formation of short-range hydrogen bonds, with several interactions persisting for prolonged simulation intervals. Although the overall hydrogen bond persistence was slightly lower than that observed for salvianolic acid A, quercetin still outperformed vigabatrin in terms of hydrogen bond stability and consistency within the ≤0.35 nm cutoff.

Scutellarein also exhibited a more consistent hydrogen bonding behavior across the simulation timeframe. The formation of short-distance hydrogen bonds, many of which remained within the ≤0.35 nm cutoff for extended periods, enhanced the stability of this compound. This suggests that scutellarein is capable of maintaining favorable polar contacts even under dynamic conformational fluctuations of the protein. Salvianolic acid A demonstrated the most pronounced hydrogen bonding network among all analyzed compounds. Due to its polyphenolic structure and high density of hydrogen bond donors and acceptors, this compound formed persistent hydrogen bonds throughout the 100 ns simulation. A substantial proportion of these interactions remained consistently below the 0.35 nm threshold, indicating strong and stable hydrogen bonding. This behavior suggests a highly favorable binding environment and implies superior anchoring of salvianolic acid A within the active site compared to vigabatrin.

Overall, comparative hydrogen bond analysis clearly demonstrates that the plant-derived aglycones, quercetin, salvianolic acid A, and scutellarein, exhibit enhanced hydrogen bonding stability relative to vigabatrin during the 100 ns molecular dynamics simulation. The increased persistence of hydrogen bonds within the accepted distance threshold suggests stronger and more reliable ligand-protein interactions.

### 2.9. MD Interaction Energy

The molecular dynamics (MD) interaction energy analysis was performed to further evaluate the binding strength and energetic stability of the top-ranked aglycones, salvianolic acid A, quercetin, and scutellarein, in comparison with the reference compound vigabatrin across three independent simulation replicates (R1–R3). The interaction energies provide a dynamic measure of ligand-protein affinity by accounting for the cumulative nonbonded interactions maintained throughout the simulation trajectory.

Salvianolic acid A exhibited the most favorable overall interaction energy among all tested aglycones, with a cumulative average interaction energy of −218.87 kcal/mol. Notably, replicate R3 showed a markedly strong interaction energy (−94.39 kcal/mol), substantially lower than those observed in R1 and R2, indicating the formation of an exceptionally stable binding state during this trajectory. Quercetin also demonstrated strong and favorable interaction energies, with a cumulative average of −199.51 kcal/mol. Replicates R1 and R2 showed particularly pronounced binding energies (−76.05 and −71.78 kcal/mol, respectively), whereas R3 displayed a comparatively weaker interaction (−51.69 kcal/mol). Although some variability was observed across replicates, the consistently strong negative interaction energies reflect persistent electrostatic and hydrogen-bond contributions, in agreement with the dense interaction network identified in the docking analysis.

Scutellarein exhibited more uniform interaction energies across all three replicates, with values ranging narrowly between −59.94 and −61.59 kcal/mol and a cumulative average of −182.90 kcal/mol (Table 4). This consistency suggests a stable and reproducible binding mode, characterized by persistent interactions throughout the simulation timeframe. Although its overall interaction energy was slightly less favorable than those of salvianolic acid A and quercetin, the low variability across replicates indicates reliable binding behavior with limited energetic fluctuations.

In comparison, the reference compound vigabatrin showed a cumulative average interaction energy of −188.70 kcal/mol, surpassing the scutellarein compound. Replicate R3 exhibited the strongest interaction energy (−74.37 kcal/mol), whereas replicates R1 and R2 showed slightly reduced binding energies (−58.18 and −56.15 kcal/mol, respectively), indicating potential and comparable binding strength to the plant-derived aglycones.

**Figure 7:**
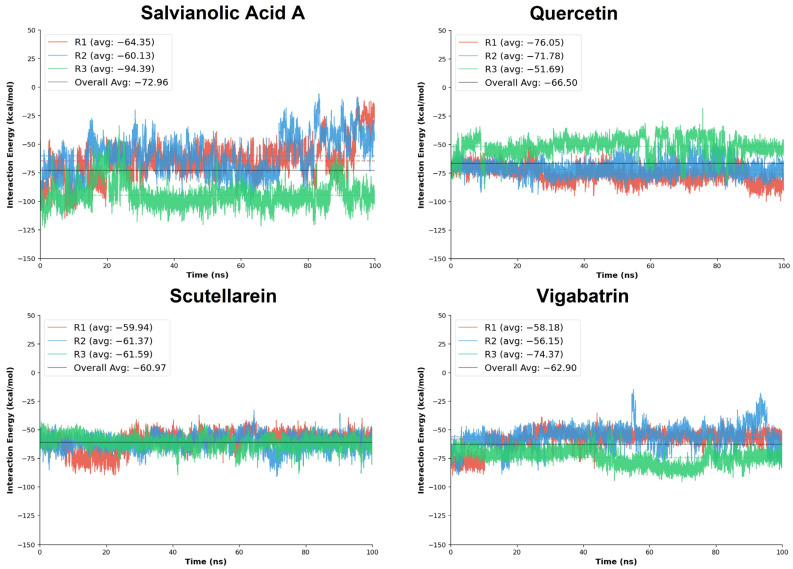
The MD interaction energy analysis was performed on the replicates of the plant-derived aglycones in comparison with the reference compound, vigabatrin.

Collectively, the MD interaction energy analysis demonstrates that the plant-derived aglycones, particularly salvianolic acid A and quercetin, exhibit more favorable and sustained interaction energies than the reference compound vigabatrin (Figure 7). Salvianolic acid A emerged as the strongest binder in energetic terms, while scutellarein displayed the most consistent interaction energy profile across replicates.

### 2.10. Free Energy Calculation

The binding free energies of the plant-derived aglycone ligands were further quantified using the Molecular Mechanics/Poisson–Boltzmann Surface Area (MM/PBSA) approach to evaluate the thermodynamic favorability of ligand-protein complex formation over the molecular dynamics trajectories [38]. The MM/PBSA calculations were performed on three independent simulation replicates (R1–R3), and the total binding free energy (ΔG_TOTAL_) was used as the primary criterion for comparative assessment, where more negative values indicate stronger binding affinity.

Among the evaluated ligands, salvianolic acid A exhibited a highly favorable binding free energy profile, with ΔG_TOTAL_ values ranging from −8.41 to −30.37 kcal/mol and an overall average of −18.01 kcal/mol. Notably, replicate R3 showed a substantially more negative free energy (−30.37 kcal/mol), suggesting the formation of a particularly stable binding conformation during this trajectory. Quercetin also demonstrated the favorable MM/PBSA results across all three replicates, with ΔG_TOTAL_ values of −19.24, −20.02, and −14.90 kcal/mol, yielding an average of −18.05 kcal/mol. Although some variability was observed across replicates, the strong average binding free energy supports the ability of the screened compounds to achieve thermodynamically favorable interactions, consistent with their extensive docking interaction network and RMSD trajectory. Scutellarein showed moderately favorable binding free energies, with ΔG_TOTAL_ values between −11.91 and −14.28 kcal/mol and an average of −13.24 kcal/mol. The relatively small variation across replicates suggests reproducible binding; however, the less negative average free energy compared to salvianolic acid A and quercetin indicates weaker thermodynamic stabilization. For comparison, the reference compound vigabatrin exhibited ΔG_TOTAL_ values ranging from −11.18 to −19.93 kcal/mol, with an average of −15.91 kcal/mol (Table 5). Vigabatrin achieved a favorable free energy in replicate R3 (−19.93 kcal/mol); moreover, its overall average binding free energy was comparable to the top tier of the aglycone ligands.

Moreover, the standard deviation analysis indicates differences in binding consistency among the screened compounds. Quercetin showed the lowest SD (±3.79), reflecting the most stable and reproducible binding across trajectories. Salvianolic acid A exhibited higher variability (±5.92); most of the SD is from run3, where the bar graphs manifested the lowest ΔG value with a varying overall trajectory. Scutellarein and vigabatrin displayed comparable moderate fluctuations (±5.82 and ±5.09, respectively), indicating reasonably consistent thermodynamic stabilization.

**Figure 8:**
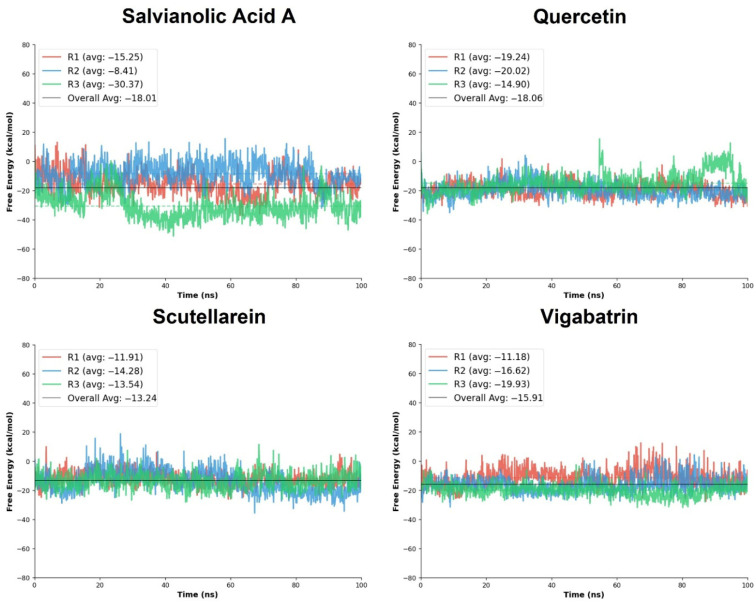
The free energy calculation graphs of the replicates of salvianolic acid A, quercetin, and scutellarein in comparison with vigabatrin.

Overall, the MM/PBSA free energy analysis indicates that salvianolic acid A and quercetin exhibit the most favorable binding free energies among the screened compounds, closely matching the reference compound vigabatrin. Quercetin showed the most consistent thermodynamic stability across simulations, whereas salvianolic acid A achieved the strongest binding in specific trajectories (Figure 8). These findings are in strong agreement with the biological analyses, collectively reinforcing the potential of these natural compounds as promising candidates for further exploration.

## 3. Materials and Methods

### 3.1. Mining of Plant-Derived Compounds in Medicinal Plants

Plant-derived compounds of valeriana officinalis, passiflora incarnate, piper methysticum, scutellaria baicalensis, melissa officinalis, humulus lupulus and magnolia officinalis were systematically collected using the KNApSAcK Family Database (http://kanaya.naist.jp/KNApSAcK_Family) (accessed on 10 September 2025). This database was utilized to systematically identify reported secondary metabolites associated with each medicinal plant species.

### 3.2. Primary Virtual Screening

The 3D structure of the GABA-AT was obtained from the Protein Data Bank (PDB; http://www.rcsb.org/, accessed on 8 September 2025, PDB ID: 4Y0H). Primary virtual screening was performed using GNINA (version 1.1), a structure-based molecular docking program that employs deep convolutional neural network-based scoring functions to predict protein-ligand binding likelihoods [31,39]. GNINA was employed in this study as a high-throughput pre-screening tool to rapidly reduce the chemical search space from a large library of plant-derived compounds.

Docking simulations were conducted by defining the active-site region of the selected GABA-AT structure as the docking grid. The docking grid was centered on the catalytically relevant active-site pocket based on the position of the co-crystallized ligand in the 4Y0H structure, with grid center coordinates set to x = 46.57, y = 21.01, z = 82.37, ensuring comprehensive coverage of key residues involved in substrate recognition and catalysis. Prior to docking, all crystallographic water molecules were removed to avoid spurious interactions, while the pyridoxal 5′-phosphate (PLP) cofactor essential for GABA-AT catalytic activity was retained to preserve the native active-site geometry. All ligands were subsequently docked into this defined binding pocket for affinity evaluation. At this stage, docking scores were used solely for coarse prioritization rather than as quantitative predictors of absolute binding affinity. Compounds were ranked to identify a meaningful subset for further refinement, and the top 30 candidates were selected for secondary analysis based on ranking consistency and predicted binding plausibility.

### 3.3. Precision-Lead Docking and Structural Validation of Binding Modes

Secondary precision docking was performed using Discovery Studio to refine and validate the binding poses and interaction patterns of the top-ranked compounds obtained from GNINA-based screening. This strategy was focused to improve pose quality and interaction realism rather than on independently validating docking scores.

In the primary screening stage, plant-derived compounds were docked in their reported chemical forms as retrieved from the phytochemical database. However, many plant-derived compounds naturally occur as glycosides, which are frequently hydrolyzed during gastrointestinal digestion and metabolism, yielding their corresponding aglycone forms as the likely biologically active species in vivo [40,41].

Accordingly, for secondary precision docking, glycosylated derivatives were excluded, and only the aglycone forms of the selected compounds were generated and retained, resulting in a refined set of 25 ligands. This step was implemented to enhance the physiological relevance of the docking analysis and to reduce structural redundancy. Prior to docking, ligand and receptor preparation procedures were carried out using Discovery Studio. Ligand preparation included energy minimization, generation of possible tautomers and stereoisomers, adjustment of ionization states, and correction of valence issues using the Ligand Preparation module. Receptor preparation involved energy minimization of the target protein, construction of missing loop regions, addition of hydrogen atoms, and protonation of the protein at pH 7.5 with an energy cutoff of 0.9, performed using the Receptor Preparation tools.

Docking simulations were subsequently conducted using the CDOCKER module in Discovery Studio, which applies a CHARMm-based molecular dynamics algorithm to explore ligand conformations and binding orientations within the GABA-AT active site. Docking results were evaluated based on CDOCKER energy and CDOCKER interaction energy values, with emphasis placed on pose consistency, interaction complementarity, and engagement of key catalytic residues, rather than on absolute score magnitudes. Protein–ligand complexes exhibiting the lowest CDOCKER energy scores (kcal/mol) were selected for subsequent biological validation, ensuring accurate assessment of binding poses and interaction stability [42].

### 3.4. Experimental Reagents and Cell Culture

Vigabatrin and quercetin dihydrate were purchased from Sigma-Aldrich (St. Louis, MO, USA). Salvianolic acid A (SalA) and scutellarein were purchased from MedChemExpress (Monmouth Junction, NJ, USA). HepG2 cells were purchased from the Korea Cell Line Bank (KCLB, #88065) and were maintained in Minimum Essential Medium (MEM; BYLABS #BY0311). The medium was supplemented with 10% heat-inactivated fetal bovine serum (FBS; GIBCO, Grand Island, NY, USA) and 100 U/mL penicillin-streptomycin (GIBCO) and cultured at 37 °C, 5% CO_2_. Cells were incubated with the reagents at the indicated concentrations for 2 days.

### 3.5. Cytotoxicity Assay

Cytotoxicity was determined by the Cellix viability assay kit (MEDiFAB, #B1007). HepG2 cells were seeded at 5 × 10^5^ cells per well in 96-well plates and cultured for 1 day. Cells were treated with the indicated concentrations of reagents and cultured for 2 days under culture conditions. After treatment, 10 μL of Cellix viability assay reagents was added to each well, and the plates were incubated for 4 h in the CO_2_ incubator. Cell viability was determined by measuring the absorbance at 450 nm with a microplate reader (SpectraMax M5, Molecular Device, San Jose, CA, USA).

### 3.6. Resazurin-Based Assay for GABA-AT Activity Determination

HepG2 Cells were plated in 6-well plates and maintained for 1 day. The cells were treated with reagents at concentrations of 1, 10, and 100 μM for 2 days without a medium change. After treatment, the cells were lysed in 200 µL of ice-cold lysis buffer composed of 100 mM sodium phosphate (pH 7.0), 20 mM pyridoxal phosphate, and 0.1% triton X-100). The lysates were freshly prepared immediately before use in the enzyme activity assay.

GABA-AT activity was quantified according to modified procedures described in previous reports [40,43,44]. Briefly, enzymatic activity was measured using a coupled reaction system employing succinic semialdehyde dehydrogenase. For each measurement, 10 µL of cell lysate was added to 190 µL of a freshly prepared reaction mixture consisting of 0.063 U/mL diaphorase, 6.25 μM resazurin, 1 mM nicotinamide adenine dinucleotide hydrate (NAD), 5 mM alpha-ketoglutarate, 3.5 mM mercaptoethanol, and 6 mM GABA in 100 mM potassium pyrophosphate buffer (pH 8.6). The lysate samples (10 µL each) were dispensed into a 96-well clear bottom black plate (Coster, Corning, NY, USA), and 190 µL of the reaction mixture was added to initiate the reaction. The plates were incubated at room temperature for 30 min while protected from light. The fluorescence signals were measured using a microplate reader at an excitation wavelength of 544 nm and an emission wavelength of 590 nm.

All resazurin-based assay data are presented as mean ± SD from four independent biological replicates (*n* = 4). For each biological replicate, fluorescence measurement was performed in technical triplicate and averaged prior to statistical analysis. Statistical significance was determined using Welch’s two-tailed t-test to compare each treatment group with the vehicle control group. This test was selected because the control group exhibited zero variance and Welch’s t-test does not assume equal variances. A *p*-value < 0.05 was considered statistically significant. Single (*), double (**), triple (***) asterisks indicate statistical significance at *p* < 0.05, *p* < 0.01, *p* < 0.001, respectively.

### 3.7. Molecular Dynamics Simulations

The three ligand-protein complexes that exhibited the lowest overall CDocker energy scores and promising to moderate biological activity were subjected to a series (Triplicates) of molecular dynamics (MD) simulations, each lasting 100 nanoseconds, to assess their dynamic stability and interactions with the target protein. The simulations were meticulously designed using parameters from the CHARMM36 force field [45,46], with input files generated through the CHARMM-GUI server [47], which significantly streamlined the setup process for GROMACS [48]. To create a suitable environment for the simulations, each system was solvated within a cubic box using the TIP3P water model. Additionally, the systems were neutralized by incorporating counter-ions and were configured with periodic boundary conditions to accurately mimic biological conditions.

For the analysis of key interactions, both electrostatic and van der Waals forces were calculated utilizing the Verlet method, with a cutoff radius of 10 Å. To enhance the precision of the electrostatic interactions, the Particle Mesh Ewald (PME) method was employed. Furthermore, the LINCS algorithm was used to constrain bond lengths, ensuring stability throughout the simulations. Prior to launching the production runs, the systems underwent a thorough energy minimization process using the steepest descent method, followed by equilibration under NVT (constant number of particles, volume, and temperature) and NPT (constant number of particles, pressure, and temperature) conditions.

The simulations were conducted using GROMACS version 2019.3, with a time step of 2 femtoseconds chosen to ensure accurate trajectory sampling. This comprehensive workflow was supported by CHARMM-GUI Python scripts (https://www.charmm-gui.org/?doc=input/solution) (accessed on 14 October 2025), which facilitated format conversion and data preparation. The outcome of this simulation process allowed for an in-depth structural and interaction analysis of the protein-ligand complexes, providing valuable insights into their dynamic stability and binding characteristics.

### 3.8. MMPBSA Binding Free Energy Calculation

The binding free energies of the protein–ligand complexes were estimated using the MM/PBSA (Molecular Mechanics/Poisson–Boltzmann Surface Area) method as implemented in gmx_MMPBSA v1.6.3 [49] for post-processing GROMACS MD trajectories. To ensure robust sampling. The complete 100 ns production trajectories from all replicas were included in the MM/PBSA calculations, avoiding truncated or selectively sampled frames. Therefore, three distinct components: the protein-ligand complex itself, the receptor, and the ligand, were examined within an explicit solvent environment.

To quantify the binding free energy (ΔG_binding_) of the lead compounds to the target protein, we utilized the following formula:

ΔG_TOTAL_ = G_complex_ − (G_protein_ + G_ligand_) (1)

In this equation, G_complex_ signifies the total energy of the protein-ligand complex, while G_protein_ and G_ligand_ denote the energies of the isolated protein and ligand, respectively, in a solvated state. This calculation accounts for various factors, including solvation effects and the inherent conformational flexibility of both components prior to their interaction. All calculations were performed using force field parameters and solvent models consistent with those applied during the MD simulations. Binding free energies were averaged over the full trajectory length for each replica, and mean ΔG values were predicted.

## 4. Conclusions

This study presents an integrated structure-based framework to identify plant-derived aglycones as potential inhibitors of GABA-AT. Through a large-scale virtual screening of 1006 phytochemicals followed by precision docking, molecular dynamics simulations, binding free energy calculations, and cell-based functional assays, we systematically evaluated both the binding feasibility and biological relevance of selected candidates. This multi-tiered strategy enabled robust prioritization of compounds with consistent performance across computational and experimental levels, thereby strengthening the reliability of the screening outcomes.

A key scientific distinction of this work lies in the aglycone-centered docking strategy adopted from the secondary precision docking stage onward. By focusing on aglycone forms—reflecting the predominant bioactive species generated after metabolic deglycosylation—this approach enhanced physiological relevance and improved predictive accuracy. Several plant-derived aglycones, notably salvianolic acid A and quercetin, exhibited stronger binding affinity, superior dynamic stability, and more favorable binding free energies than the reference inhibitor vigabatrin. These computational findings were further corroborated by cell-based assays, in which the same compounds demonstrated significant inhibition of intracellular GABA-AT activity.

Collectively, these results establish plant-derived aglycones as a promising and underexplored class of GABA-AT inhibitors, highlighting the value of integrating metabolically informed compound selection with comprehensive in silico and in vitro validation. The strategy presented here provides a rational bridge between natural product chemistry and molecular pharmacology and offers a scalable platform for future studies. Further investigation through in vivo evaluation, structure-activity relationship optimization, and central nervous system exposure assessment will be essential to fully realize the therapeutic potential of these compounds in GABA-related neurological disorders.

## Figures and Tables

**Table 1:** Top 30 GABA-AT binding plant-derived compounds ranked by GNINA docking scores.

Medicinal Plants	Plant-Derived Compounds	Docking Score (kcal/mol)
*Melissa officinalis*	Yunnaneic acid F	−9.18
*Passiflora incarnata*	Isoorientin 2″-O-glucopyranoside	−8.67
*Humulus lupulus*	Quercetin 3-rhamnosyl-(1->2)-rhamnosyl-(1->6)-glucoside	−8.64
*Scutellaria baicalensis*	Acteoside	−8.51
*Humulus lupulus*	Quercetin 3-rutinoside	−8.48
*Humulus lupulus*	Manghaslin	−8.47
*Melissa officinalis*	Luteolin 3′-O-beta-D-glucuronide	−8.42
*Melissa officinalis*	Salvianolic acid B	−8.38
*Scutellaria baicalensis*	Leucosceptoside A	−8.33
*Scutellaria baicalensis*	Chrysin 6-C-arabinoside 8-C-glucoside	−8.27
*Melissa officinalis*	Salvianolic acid A	−8.21
*Melissa officinalis*	Luteolin 7-glucoside-3′-glucuronide	−8.12
*Scutellaria baicalensis*	Jionoside D	−8.05
*Magnolia officinalis*	Magnoloside B	−8.05
*Scutellaria baicalensis*	Dihydrobaicalein 7-O-glucuronide	−8.03
*Scutellaria baicalensis*	Scutellarein 7-glucuronide	−8.02
*Scutellaria baicalensis*	Chrysin 6-C-glucoside 8-C-arabinoside	−8.01
*Scutellaria baicalensis*	Baicalin	−8.00
*Scutellaria baicalensis*	5,7,2′,6′-Tetrahydroxyflavone 2′-O-glucoside	−7.99
*Scutellaria baicalensis*	Apigenin 7-O-beta-D-glucuronide	−7.98
*Humulus lupulus*	Kaempferitrin	−7.98
*Magnolia officinalis*	Magnolignan I	−7.98
*Scutellaria baicalensis*	Oroxin A	−7.95
*Scutellaria baicalensis*	Chrysin 7-glucuronide	−7.94
*Melissa officinalis*	Melitric acid A	−7.91
*Valeriana officinalis*	Kanokoside D	−7.89
*Magnolia officinalis*	Isoacteoside	−7.89
*Humulus lupulus*	Quercetin 3-(6″-malonylglucoside)	−7.88
*Scutellaria baicalensis*	Viscidulin II 2′-O-glucoside	−7.87
*Magnolia officinalis*	Magnoloside A	−7.87

**Table 2:** Comparison of molecular docking energetics for the top ten plant-derived aglycones and vigabatrin as determined by the Discovery Studio CDocker module, ranked based on the lowest CDocker energy.

Plant-Derived Aglycones	CDocker Energy (kcal/mol)	CDocker Interaction Energy (kcal/mol)
Salvianolic acid A	−61.1521	−69.1719
Quercetin	−57.4877	−55.0657
Melitric acid A	−55.7288	−68.2939
Scutellarein	−54.8822	−58.5430
Tetrahydroxyflavone	−50.4093	−49.9079
Baicalin	−46.9267	−78.9246
Salvianolic acid B	−46.5809	−73.9209
Apigenin	−45.8413	−54.7887
Vigabatrin (Ref)	−42.0196	−44.5694
Dihydrobaicalein	−35.1200	−37.0547
Luteolin	−35.0814	−39.6503

**Table 3:** The interaction profile of the top 4 plant-derived aglycones in comparison with the reference compound (vigabatrin). The bolds in the table represent the salt bridges.

Plant-Derived Aglycones	Interacting Amino Acids	Binding Distance
Salvianolic Acid A	Lys357	1.95 Å
	Asn168	2.53 Å
	Gln329	2.56 Å
	Glu69	2.02 Å
	Arg220	2.67 Å
Quercetin	Lys357	1.64 Å
	Glu293	2.94 Å
	Phe217	2.00 Å
	Ser165	2.55 Å
Melitric Acid A	Lys357	1.83 Å, 2.24 Å
	Glu69	2.06 Å
	Ser297	1.87 Å
	Asn168	2.29 Å
Scutellarein	Arg220	2.15 Å
	Lys357	1.90 Å, 1.89 Å
	Asp326	2.13 Å, 2.02 Å
Vigabatrin	Glu293	2.15 Å
	Phe217	2.64 Å
	Lys357	1.88 Å
	Gln329	2.69 Å

**Table 4:** The MD interaction energy comparison of the plant-derived aglycones against the reference compound, vigabatrin.

Plant-Derived Aglycones	Interaction Energy (kcal/mol)			Average
	R1	R2	R3	
Salvianolic Acid A	−64.3461	−60.1324	−94.3879	−72.9554
Quercetin	−76.0486	−71.7777	−51.6866	−66.5043
Scutellarein	−59.9390	−61.3691	−61.5881	−60.9654
Vigabatrin	−58.1779	−56.1535	−74.3710	−62.9008

**Table 5:** Thermodynamic free energy (ΔG) of plant-derived aglycones compared to vigabatrin, including standard deviation (SD) across experimental runs.

Plant-Derived Aglycones	ΔG_TOTAL_ (kcal/mol)			Average ± SD
	R1	R2	R3	
Salvianolic Acid A	−15.25	−8.41	−30.37	−18.01 ± 5.92
Quercetin	−19.24	−20.02	−14.90	−18.05 ± 3.79
Scutellarein	−11.91	−14.28	−13.54	−13.24 ± 5.82
Vigabatrin	−11.18	−16.62	−19.93	−15.91 ± 5.09

## Data Availability

The original contributions presented in this study are included in the article. Further inquiries can be directed to the corresponding author.

## Supplementary Materials

The following supporting information can be downloaded at: https://www.mdpi.com/article/10.3390/ph19020307/s1, Figure S1. Cytotoxicity assessment of plant-derived aglycones in HepG2 cells; Figure S2: Two-dimensional interaction image of the top-ranked plant-derived aglycones and the reference compound vigabatrin within the binding pocket, highlighting key hydrogen bonds, salt bridges, and hydrophobic contacts with active-site residues; Table S1: Complete primary virtual screening results of plant-derived compounds against GABA-AT.
