# Supplementary material for: Discovery of Plant-Derived Natural Compounds as Novel GABA Aminotransferase Inhibitors: Structure-Based Discovery, Experimental Validation, and Molecular Dynamics Analysis
Source: Pharmaceuticals (Basel). 2026 Feb 12;19(2):307. doi: 10.3390/ph19020307 (PMC12944523; doi:10.3390/ph19020307)
Supplement: Supplementary file 1 [file pharmaceuticals-19-00307-s001.zip › pharmaceuticals-4139023-supplementary(1)/Supplementary information.docx]

Article

Discovery of Plant-Derived Natural Compounds as Novel GABA Aminotransferase Inhibitors:
Structure-Based Discovery, Experimental Validation, and Molecular Dynamics Analysis

Jinyoung Park ^1^, Muhammad Yasir ^1^, Eun-Taek Han ^2^, Won Sun Park ^3^, Jin-Hee Han ^2^, Jongseon Choe ^4^ and Wanjoo Chun ^1,^*

^1^ Department of Pharmacology, School of Medicine, Kangwon National University,
Chuncheon 24341, Republic of Korea; jinyoung0326@kangwon.ac.kr (J.P.);
yasir.khokhar1999@gmail.com (M.Y.)

^2^ Department of Medical Environmental Biology and Tropical Medicine, School of Medicine, Kangwon National University, Chuncheon 24341, Republic of Korea; ethan@kangwon.ac.kr (E.-T.H.); han.han@kangwon.ac.kr (J.-H.H.)

^3^ Department of Physiology, School of Medicine, Kangwon National University, Chuncheon 24341, Republic of Korea; parkws@kangwon.ac.kr

^4^ Department of Microbiology and Immunology, School of Medicine, Kangwon National University, Chuncheon 24341, Republic of Korea; jchoe@kangwon.ac.kr

***** Correspondence: wchun@kangwon.ac.kr; Tel.: +82-33-250-8853


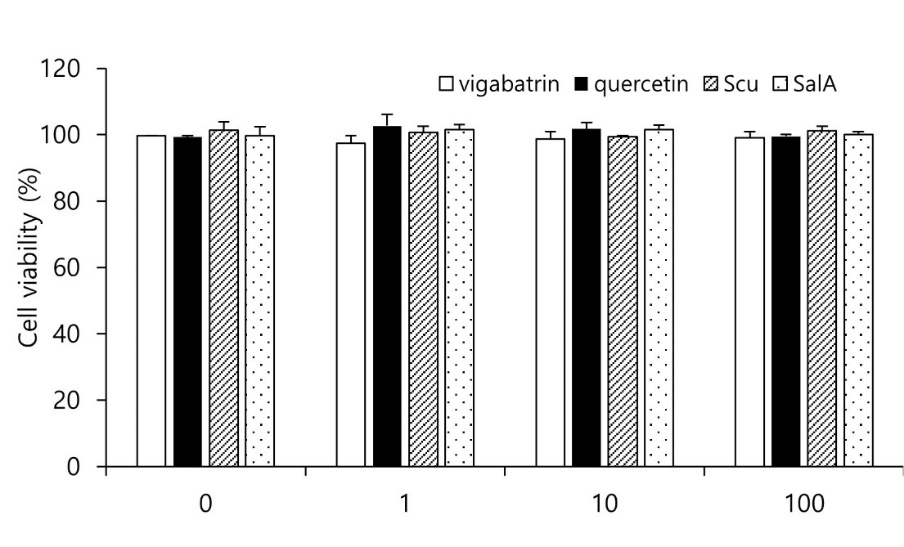


**Scheme S1.** Cytotoxicity assessment of plant-derived aglycones in HepG2 cells. Cells were treated with the vigabatrin, quercetin, scutellarein, or salvianolic acid A at indicated concentration (1, 10, 100 μM) for 2 days, and cell viability was determined by Cellix viability assay kit.


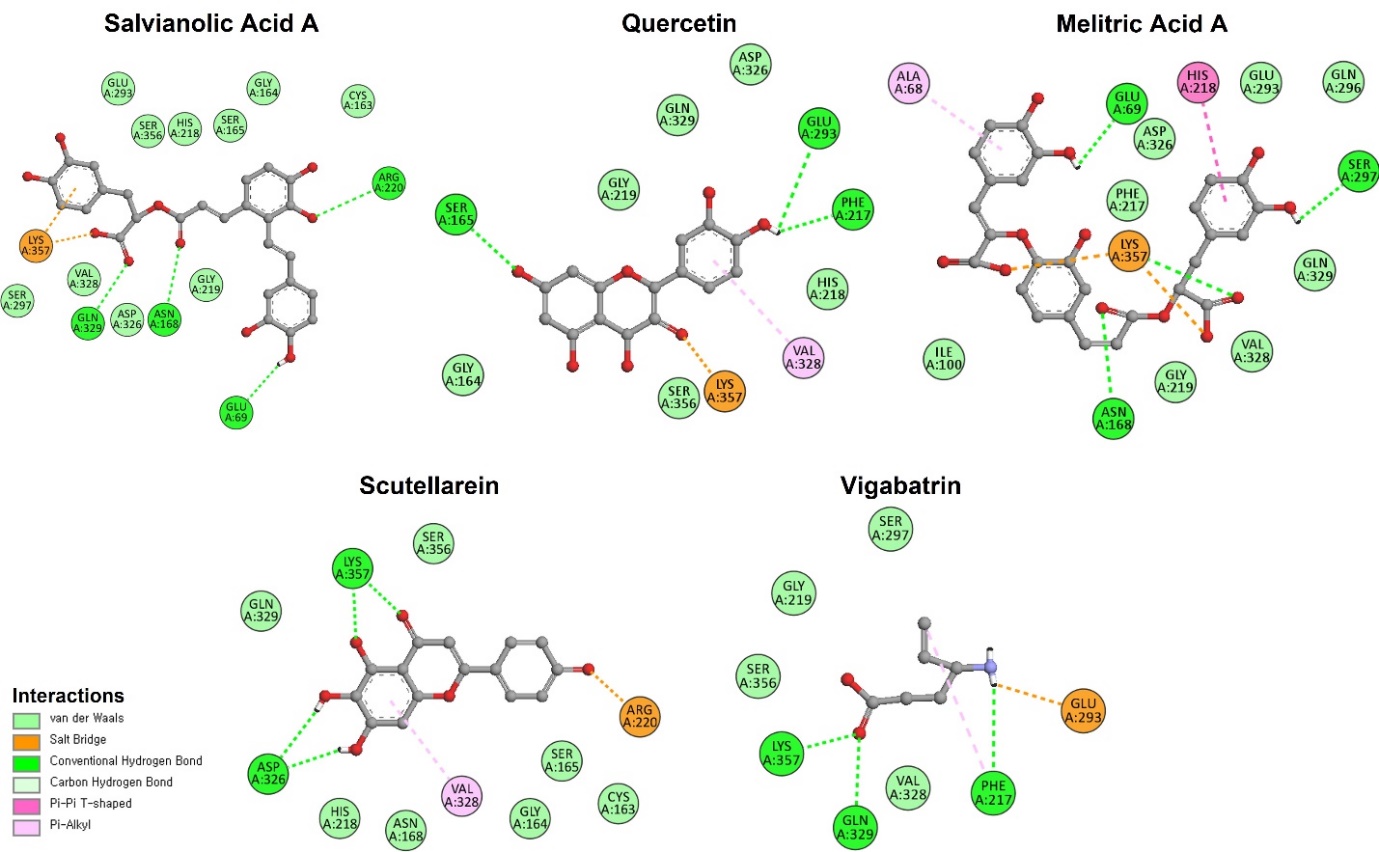


**Scheme S2.** Two-dimensional interaction image of the top-ranked plant-derived aglycones and the reference compound vigabatrin within the binding pocket, highlighting key hydrogen bonds, salt bridges, and hydrophobic contacts with active-site residues.
