# Supplementary material for: Discovery of Plant-Derived Natural Compounds as Novel GABA Aminotransferase Inhibitors: Structure-Based Discovery, Experimental Validation, and Molecular Dynamics Analysis
Source: Pharmaceuticals (Basel). 2026 Feb 12;19(2):307. doi: 10.3390/ph19020307 (PMC12944523; doi:10.3390/ph19020307)
Supplement: Supplementary file 1 [file pharmaceuticals-19-00307-s001.zip › pharmaceuticals-4139023-supplementary(1)/Supplementary Table S1:Complete primary virtual screening results of plant-derived compounds against GABA-AT .pdf]

Organism	SMILES	Docking score (kcal/mol)
Melissa officinalis	O=C(/C=C/C1=C[C@@H]2[C@H](c3ccc(O)c(O)c3)[C@@H](C(=O)O)[C@H]1[C@]1(O)OC(=O)C[C@]21O)O[C@H](Cc1ccc(O)c(O)c1)C(=O)O	-9.18
Passiflora incarnata	O=c1cc(-c2ccc(O)c(O)c2)oc2cc(O)c([C@@H]3OC(CO)[C@@H](O)[C@H](O)C3O[C@@H]3OC(CO)[C@@H](O)[C@H](O)C3O)c(O)c12	-8.67
Humulus lupulus	CC1O[C@@H](OC2[C@H](OCC3O[C@@H](Oc4c(-c5ccc(O)c(O)c5)oc5cc(O)cc(O)c5c4=O)C(O)C(O)[C@@H]3O)OC(C)[C@H](O)[C@@H]2O)C(O)[C@@H](O)[C@H]1O	-8.64
Scutellaria baicalensis	CC1O[C@@H](O[C@@H]2C(O)[C@H](OCCc3ccc(O)c(O)c3)OC(CO)[C@H]2OC(=O)/C=C/c2ccc(O)c(O)c2)C(O)[C@@H](O)[C@H]1O	-8.51
Humulus lupulus	CC1O[C@@H](OCC2O[C@@H](Oc3c(-c4ccc(O)c(O)c4)oc4cc(O)cc(O)c4c3=O)C(O)C(O)[C@@H]2O)C(O)[C@@H](O)[C@H]1O	-8.48
Humulus lupulus	CC1O[C@@H](OC2C(O)[C@H](O)C(CO[C@@H]3OC(C)[C@H](O)[C@H](O)C3O)O[C@H]2Oc2c(-c3ccc(O)c(O)c3)oc3cc(O)cc(O)c3c2=O)[C@@H](O)C(O)[C@H]1O	-8.47
Melissa officinalis	O=C(O)C1O[C@@H](Oc2cc(-c3cc(=O)c4c(O)cc(O)cc4o3)ccc2O)C(O)[C@@H](O)[C@@H]1O	-8.42
Melissa officinalis	O=C(/C=C/c1ccc(O)c2c1[C@H](C(=O)O[C@H](Cc1ccc(O)c(O)c1)C(=O)O)[C@@H](c1ccc(O)c(O)c1)O2)O[C@H](Cc1ccc(O)c(O)c1)C(=O)O	-8.38
Scutellaria baicalensis	COc1cc(/C=C/C(=O)O[C@H]2[C@H](O[C@@H]3O[C@@H](C)[C@H](O)[C@@H](O)[C@H]3O)[C@@H](O)[C@H](OCCc3ccc(O)c(O)c3)O[C@@H]2CO)ccc1O	-8.33
Scutellaria baicalensis	O=c1cc(-c2ccccc2)oc2c(C3OC(CO)C(O)C(O)C3O)c(O)c(C3OCC(O)C(O)C3O)c(O)c12	-8.27
Melissa officinalis	O=C(/C=C/c1ccc(O)c(O)c1/C=C/c1ccc(O)c(O)c1)O[C@H](Cc1ccc(O)c(O)c1)C(=O)O	-8.21
Melissa officinalis	O=C(O)C1O[C@@H](Oc2cc(-c3cc(=O)c4c(O)cc(O[C@@H]5OC(CO)[C@@H](O)[C@H](O)C5O)cc4o3)ccc2O)C(O)[C@@H](O)[C@@H]1O	-8.12
Scutellaria baicalensis	COc1ccc(CCO[C@@H]2O[C@H](CO)[C@@H](OC(=O)/C=C/c3ccc(O)c(O)c3)[C@H](O[C@@H]3O[C@@H](C)[C@H](O)[C@@H](O)[C@H]3O)[C@H]2O)cc1O	-8.05
Magnolia officinalis	CC1OC(OC2C(OCCc3ccc(O)c(O)c3)OC(COC3OC(CO)C(O)C(O)C3O)C(O)C2OC(=O)C=Cc2ccc(O)c(O)c2)C(O)C(O)C1O	-8.05
Scutellaria baicalensis	O=C1CC(c2ccccc2)Oc2cc(O[C@@H]3OC(C(=O)O)[C@@H](O)[C@H](O)C3O)c(O)c(O)c21	-8.03
Scutellaria baicalensis	O=C(O)C1O[C@@H](Oc2cc3oc(-c4ccc(O)cc4)cc(=O)c3c(O)c2O)C(O)[C@@H](O)[C@@H]1O	-8.02
Scutellaria baicalensis	O=c1cc(-c2ccccc2)oc2c(C3OCC(O)C(O)C3O)c(O)c(C3OC(CO)C(O)C(O)C3O)c(O)c12	-8.01
Scutellaria baicalensis	O=C(O)C1O[C@@H](Oc2cc3oc(-c4ccccc4)cc(=O)c3c(O)c2O)C(O)[C@@H](O)[C@@H]1O	-8
Scutellaria baicalensis	O=c1cc(-c2c(O)cccc2O[C@@H]2OC(CO)[C@@H](O)[C@H](O)C2O)oc2cc(O)cc(O)c12	-7.99
Scutellaria baicalensis	O=C(O)C1O[C@@H](Oc2cc(O)c3c(=O)cc(-c4ccc(O)cc4)oc3c2)C(O)[C@@H](O)[C@@H]1O	-7.98
Humulus lupulus	CC1O[C@@H](Oc2c(-c3ccc(O)cc3)oc3cc(O[C@@H]4OC(C)[C@H](O)[C@H](O)C4O)cc(O)c3c2=O)[C@@H](O)C(O)[C@H]1O	-7.98
Magnolia officinalis	C=CCc1ccc(O)c(-c2cc([C@H]3Oc4c(-c5cc(CC=C)ccc5O)cc(O)cc4[C@@H]3CO)ccc2O)c1	-7.98
Scutellaria baicalensis	O=c1cc(-c2ccccc2)oc2cc(OC3OC(CO)C(O)C(O)C3O)c(O)c(O)c12	-7.95
Scutellaria baicalensis	O=C(O)C1O[C@@H](Oc2cc(O)c3c(=O)cc(-c4ccccc4)oc3c2)C(O)[C@@H](O)[C@@H]1O	-7.94
Melissa officinalis	O=C(/C=C/c1ccc(O/C(=Cc2ccc(O)c(O)c2)C(=O)O)c(O)c1)O[C@H](Cc1ccc(O)c(O)c1)C(=O)O	-7.91
Valeriana officinalis	CC(C)CC(=O)O[C@@H]1OC=C(CO[C@H]2OC(CO[C@@H]3OC(CO)[C@@H](O)C(O)[C@@H]3O)[C@@H](O)C(O)[C@@H]2O)[C@H]2C[C@H](O)[C@@H](CO)[C@@H]12	-7.89
Magnolia officinalis	C[C@@H]1O[C@@H](O[C@@H]2[C@@H](O)[C@H](OCCc3ccc(O)c(O)c3)O[C@H](COC(=O)/C=C/c3ccc(O)c(O)c3)[C@H]2O)[C@H](O)[C@H](O)[C@H]1O	-7.89
Humulus lupulus	O=C(O)CC(=O)OC[C@H]1O[C@@H](Oc2c(-c3ccc(O)c(O)c3)oc3cc(O)cc(O)c3c2=O)C(O)C(O)[C@@H]1O	-7.88
Scutellaria baicalensis	COc1cc(O)c2c(=O)cc(-c3c(O)cccc3OC3OC(CO)C(O)C(O)C3O)oc2c1OC	-7.87
Magnolia officinalis	CC1OC(OC2C(OCCc3ccc(O)c(O)c3)OC(CO)C(O)C2OC(=O)C=Cc2ccc(O)c(O)c2)C(O)C(O)C1O	-7.87
Scutellaria baicalensis	O=c1cc(-c2ccccc2)oc2c([C@@H]3OC(CO)[C@@H](O)[C@H](O)C3O)c(O)cc(O)c12	-7.82
Humulus lupulus	CC1O[C@@H](OCC2O[C@@H](Oc3c(-c4ccc(O)cc4)oc4cc(O)cc(O)c4c3=O)C(O)C(O)[C@@H]2O)C(O)[C@@H](O)[C@H]1O	-7.82
Melissa officinalis	O=C(O[C@@H](Cc1ccc(O)c(O)c1)C(=O)O)[C@H]1[C@H](C(=O)O[C@H](Cc2ccc(O)c(O)c2)C(=O)O)[C@H](c2ccc(O)c(O)c2)[C@H]1c1ccc(O)c(O)c1	-7.8
Humulus lupulus	O=C(O)CC(=O)OC[C@H]1O[C@@H](Oc2c(-c3ccc(O)cc3)oc3cc(O)cc(O)c3c2=O)C(O)C(O)[C@@H]1O	-7.8
Magnolia officinalis	CC1O[C@@H](O[C@@H]2C(O)[C@H](OCCc3ccc(O)c(O)c3)OC(CO)[C@H]2OC(=O)/C=C/c2ccc(O)c(O)c2)C(O)[C@@H](O)[C@H]1O	-7.78
Scutellaria baicalensis	COC(=O)C1OC(Oc2cc3oc(-c4ccccc4)cc(=O)c3c(O)c2O)C(O)C(O)C1O	-7.77
Scutellaria baicalensis	O=c1cc(-c2ccccc2)oc2c([C@@H]3OC(CO)[C@@H](O)[C@H](O)C3O)c(O)c([C@@H]3OC[C@H](O)[C@H](O)C3O)c(O)c12	-7.7
Melissa officinalis	O=C(/C=C/c1ccc(O)c2c1C(C(=O)O)C(c1ccc(O)c(O)c1)O2)O[C@H](Cc1ccc(O)c(O)c1)C(=O)O	-7.7
Magnolia officinalis	C=CCc1ccc(Oc2cc(C(O)C(O)Cc3c(CC=C)cc(O)c(O)c3Oc3ccc(CC=C)cc3)cc(O)c2O)cc1	-7.7
Scutellaria baicalensis	COc1ccc(CCO[C@@H]2O[C@H](CO)[C@@H](OC(=O)/C=C/c3ccc(O)c(OC)c3)[C@H](O[C@@H]3O[C@@H](C)[C@H](O)[C@@H](O)[C@H]3O)[C@H]2O)cc1O	-7.69
Humulus lupulus	CC1O[C@@H](OC2[C@H](OCC3O[C@@H](Oc4c(-c5ccc(O)cc5)oc5cc(O)cc(O)c5c4=O)C(O)C(O)[C@@H]3O)OC(C)[C@H](O)[C@@H]2O)C(O)[C@@H](O)[C@H]1O	-7.68
Magnolia officinalis	CC1OC(OC2C(OCCc3ccc(O)c(O)c3)OC(CO)C(OC(=O)C=Cc3ccc(O)c(O)c3)C2O)C(O)C(O)C1O	-7.68
Passiflora incarnata	O=c1cc(-c2ccc(O)cc2)oc2cc(O)c([C@@H]3OC(CO)[C@@H](O)[C@H](O)C3O[C@@H]3OC(CO)[C@@H](O)[C@H](O)C3O)c(O)c12	-7.66
Humulus lupulus	CC1O[C@@H](Oc2c(-c3ccc(O)c(O)c3)oc3cc(O)cc(O)c3c2=O)[C@@H](O)C(O)[C@H]1O	-7.65
Magnolia officinalis	C=CCc1ccc(Oc2cc(CC=C)c3c(c2O)O[C@@H](CO)[C@H]3c2ccc(O)c(-c3cc(CC=C)ccc3O)c2)cc1	-7.65
Valeriana officinalis	COc1cc([C@H]2OC[C@H]3[C@@H]2CO[C@@H]3c2ccc(O[C@@H]3O[C@H](CO)[C@@H](O)[C@H](O)[C@H]3O)c(OC)c2)ccc1O[C@@H]1O[C@H](CO)[C@@H](O)[C@H](O)[C@H]1O	-7.59
Humulus lupulus	CC(C)=CCC/C(C)=C/Cc1c(O)cc(O)c2c1OC(c1ccc(O)cc1)CC2=O	-7.46
Humulus lupulus	CC(C)=CCC/C(C)=C/Cc1c(O)cc2c(c1O)C(=O)CC(c1ccc(O)cc1)O2	-7.46
Passiflora incarnata	O=c1cc(-c2ccc(O)cc2)oc2cc(O[C@@H]3OC(CO)[C@@H](O)[C@H](O)C3O)c([C@@H]3OC(CO)[C@@H](O)[C@H](O)C3O)c(O)c12	-7.45
Valeriana officinalis	COc1cc([C@H]2OC[C@H]3[C@@H]2CO[C@@H]3c2ccc(O[C@@H]3O[C@H](CO)[C@@H](O)[C@H](O)[C@H]3O)c(OC)c2)ccc1O	-7.42
Scutellaria baicalensis	O=c1c(-c2ccc(O)cc2)coc2c([C@@H]3OC(CO)[C@@H](O)[C@H](O)C3O)c(O)ccc12	-7.42
Valeriana officinalis	COc1cc([C@H]2OC[C@]3(O[C@@H]4O[C@H](CO)[C@@H](O)[C@H](O)[C@H]4O)[C@@H](c4ccc(O)c(OC)c4)OC[C@H]23)ccc1O	-7.4
Scutellaria baicalensis	O=C1CC(c2ccc(O)cc2)Oc2cc(O)c(O)c(O)c21	-7.39
Melissa officinalis	O=C(C=Cc1ccc2c(c1)OC(=O)C(=Cc1ccc(O)c(O)c1)O2)OC(Cc1ccc(O)c(O)c1)C(=O)O	-7.36
Scutellaria baicalensis	COc1c(OC)c(O)c2c(=O)cc(-c3ccccc3O)oc2c1O	-7.34
Humulus lupulus	O=C(O)CC(=O)OC[C@H]1O[C@@H](Oc2c(-c3ccc(O)c(O)c3)oc3cc(O)cc(O)c3c2=O)C(O)C(O)[C@H]1O	-7.34
Scutellaria baicalensis	COc1cc(-c2coc3cc(O[C@@H]4OC(CO)[C@@H](O)[C@H](O)C4O)c(OC)c(O)c3c2=O)cc(O)c1OC	-7.33
Humulus lupulus	O=C(O)CC(=O)OCC1OC(Oc2c(-c3ccc(O)cc3)oc3cc(O)cc(O)c3c2=O)C(O)C(O)C1O	-7.33
Humulus lupulus	COc1cc2c(c(O)c1C(=O)/C=C/c1ccc(O)cc1)CC(O)C(C)(C)O2	-7.33
Magnolia officinalis	C=CCc1ccc(O)c(-c2cc(C(O)C(O)COc3ccc(CC=C)cc3-c3cc(CC=C)ccc3O)ccc2O)c1	-7.31
Scutellaria baicalensis	O=c1c(O)c(-c2ccccc2)oc2cc(O)cc(O)c12	-7.26
Scutellaria baicalensis	O=c1cc(-c2ccc(O)cc2)oc2c([C@@H]3OC(CO)[C@@H](O)[C@H](O)C3O)c(O)c([C@@H]3OC[C@H](O)[C@H](O)C3O)c(O)c12	-7.26
Humulus lupulus	CC(C)=CCC/C(C)=C/Cc1c(O)cc(O)c(C(=O)/C=C/c2ccc(O)cc2)c1O	-7.26
Valeriana officinalis	COc1cc(C2c3cc(OC4OC(CO)C(O)C(O)C4O)c(OC)cc3CC(O)(CO)C2CO)ccc1O	-7.22
Passiflora incarnata	O=c1cc(-c2ccc(O)c(O)c2)oc2c([C@@H]3OC(CO)[C@@H](O)[C@H](O)C3O)c(O)cc(O)c12	-7.22
Humulus lupulus	O=c1c(O[C@@H]2O[C@H](CO)[C@H](O)C(O)C2O)c(-c2ccc(O)c(O)c2)oc2cc(O)cc(O)c12	-7.21
Scutellaria baicalensis	COc1cc(O)c2c(=O)cc(-c3ccccc3O)oc2c1OC	-7.2
Scutellaria baicalensis	O=c1cc(-c2ccccc2)oc2c([C@@H]3OC[C@H](O)[C@H](O)C3O)c(O)c([C@@H]3OC(CO)[C@@H](O)[C@H](O)C3O)c(O)c12	-7.17
Humulus lupulus	CC1O[C@@H](OC2[C@H](Oc3c(-c4ccc(O)cc4)oc4cc(O)cc(O)c4c3=O)OC(COC(=O)CC(=O)O)[C@@H](O)[C@@H]2O)[C@@H](O)C(O)[C@H]1O	-7.16
Humulus lupulus	CC1O[C@@H](OC2C(O)[C@H](O)C(CO)O[C@H]2Oc2c(-c3ccc(O)cc3)oc3cc(O)cc(O)c3c2=O)[C@@H](O)C(O)[C@H]1O	-7.15
Scutellaria baicalensis	COc1c(O)ccc(O[C@@H]2O[C@@H](CO)[C@@H](O)C(O)C2O)c1-c1cc(=O)c2c(O)cc(O)c(OC)c2o1	-7.13
Scutellaria baicalensis	O=C1CC(c2cc(O)ccc2O)Oc2cc(O)cc(O)c21	-7.12
Magnolia officinalis	O=C(C=Cc1ccc(O)c(O)c1)OCC1OC(OCCc2ccc(O)c(O)c2)C(OC2OCC(O)(CO)C2O)C(O)C1O	-7.12
Humulus lupulus	CC(C)=CCC(=O)C1(O)C(O)=C(C(=O)C(C)C)C(=O)C1(O)CC=C(C)C	-7.11
Magnolia officinalis	COc1ccc(C[C@H]2c3cc(O)c(OC)cc3CCN2C)cc1O	-7.11
Melissa officinalis	O=C(/C=C/c1ccc(O)c(O)c1)O/C=Cc1cc(O)cc(O)c1	-7.1
Scutellaria baicalensis	COc1c(O)cc2c(c1O)C(=O)CC(c1ccc(O)cc1)O2	-7.04
Scutellaria baicalensis	O=C1CC(c2ccc(O)c(O)c2)Oc2cc(O)cc(O)c21	-7.04
Scutellaria baicalensis	COc1c(O[C@@H]2OC(C(=O)O)[C@@H](O)[C@H](O)C2O)cc(O)c2c(=O)cc(-c3ccccc3)oc12	-7.03

Valeriana officinalis	CC(C)CC(=O)O[C@@H]1OC=C(CO[C@@H]2OC(CO)[C@@H](O)C(O)[C@@H]2O)[C@H]2C[C@H](O)[C@@](C)(O)[C@@H]12	-6.97
Valeriana officinalis	CC(C)CC(=O)O[C@@H]1OC=C(CO[C@H]2OC(CO[C@@H]3OC(CO)[C@@H](O)C(O)[C@@H]3O)[C@@H](O)C(O)[C@@H]2O)[C@H]2[C@@H]3O[C@@H]3[C@@](O)(CO)[C@@H]12	-6.94
Melissa officinalis	O=C(O)/C=C/c1ccc(O)c(O)c1/C=C/c1ccc(O)c(O)c1	-6.94
Humulus lupulus	CC(C)C(=O)c1c(O)cc(OC2OC(CO)C(O)C(O)C2O)cc1O	-6.93
Humulus lupulus	COc1cc(O)c2c(c1C(=O)C=Cc1ccc(O)cc1)OC(C(C)(C)O)C2	-6.9
Melissa officinalis	O=C(/C=C/c1ccc(O)c(O)c1)O/C=Cc1ccc(O)c(O)c1	-6.89
Valeriana officinalis	COc1cc([C@H]2OC[C@]3(O)[C@@H](c4ccc(O[C@@H]5O[C@H](CO)[C@@H](O)[C@H](O)[C@H]5O)c(OC)c4)OC[C@]23O)ccc1O	-6.88
Humulus lupulus	COc1cc(O)c(CC=C(C)C)c(O)c1C(=O)/C=C/c1ccc(O)cc1	-6.87
Humulus lupulus	CC(C)=CCc1c(O)cc2c(c1O)C(=O)C[C@@H](c1ccc(O)cc1)O2	-6.87
Scutellaria baicalensis	O=c1cc(-c2ccccc2)oc2cc(O)c([C@@H]3OC(CO)[C@@H](O)[C@H](O)C3O)c(O)c12	-6.82
Humulus lupulus	CC(=CCNc1ncnc2nc[nH]c12)COC1OC(CO)C(O)C(O)C1O	-6.82
Valeriana officinalis	CC1(C)C2CC(OC3OC(COC4OCC(O)(CO)C4O)C(O)C(O)C3O)C1(C)CC2O	-6.81
Humulus lupulus	COc1cc(O)c(CC=C(C)C)c(O)c1C(=O)CCc1ccc(O)cc1	-6.78
Humulus lupulus	O=C1C[C@@H](c2ccc(O)cc2)Oc2cc(O)cc(O)c21	-6.78
Humulus lupulus	COc1cc(O)c(C(=O)/C=C/c2ccccc2)c(O)c1CC=C(C)C	-6.77
Valeriana officinalis	C=C(C)[C@H]1Cc2c(ccc3c2O[C@@H]2COc4cc(OC)c(OC)cc4[C@@H]2C3=O)O1	-6.76
Magnolia officinalis	C=CCc1cc(-c2cc(CC=C)cc(C3C=C(C)CCC3C(C)C)c2O)c(O)c(C2C=C(C)CCC2C(C)C)c1	-6.75
Melissa officinalis	O=C(/C=C/c1ccc(O)c(O)c1)O[C@H](Cc1ccc(O)c(O)c1)C(=O)O	-6.74
Magnolia officinalis	C=CCc1cc(-c2ccc(O)c(CC=C)c2)c(O)c(C2C=C(C)CCC2C(C)C)c1	-6.74
Humulus lupulus	C=C(C)C(O)Cc1c(O)cc(OC)c(C(=O)/C=C/c2ccc(O)cc2)c1O	-6.73
Humulus lupulus	O=c1c(O[C@@H]2O[C@H](CO)[C@@H](O)C(O)C2O)c(-c2ccc(O)c(O)c2)oc2cc(O)cc(O)c12	-6.73
Valeriana officinalis	COc1cc(C2OCC3(O)C(c4ccc(O)c(OC)c4)OCC23)ccc1O	-6.72
Scutellaria baicalensis	O=c1cc(-c2ccccc2)oc2cc(O)cc(O)c12	-6.72
Valeriana officinalis	COc1cc(C2OC[C@@H]3[C@@H](c4ccc(O)c(OC)c4)OC[C@H]23)ccc1O	-6.7
Valeriana officinalis	CC(C)CC(=O)OC[C@@]1(O)[C@H]2[C@H](OC(=O)CC(C)C)OC=C(CO[C@@H]3O[C@H](CO[C@@H]4O[C@H](CO)[C@@H](O)[C@H](O)[C@H]4O)[C@@H](O)[C@H](O)[C@H]3O)[C@H]2[C@@H]2O[C@@H]21	-6.7
Valeriana officinalis	CC1CCC=C2CCC(C(C)(C)OC(=O)c3cc([N+](=O)[O-])cc([N+](=O)[O-])c3)CC21C	-6.68
Magnolia officinalis	COc1ccc2c(c1O)-c1c(O)c(OC)cc3c1[C@H](C2)[N+](C)(C)CC3	-6.67
Melissa officinalis	O=C(/C=C/c1ccc(O)cc1)O[C@H](Cc1ccc(O)c(O)c1)C(=O)O	-6.66
Humulus lupulus	CC(C)=CC1OC(C)(C)C2CC3(O)C(=O)C(C(=O)CC(C)C)=C(O)C3(O)C12O	-6.66
Humulus lupulus	C=C(C)[C@H]1C[C@]23C(=O)C(C(=O)CC(C)C)=C(O)[C@@]2(O)C(=O)C[C@@H]3C1(C)C	-6.66
Piper methysticum	CN1C(=O)C(=O)c2cc3c(c4c2c1cc1ccccc14)OCO3	-6.65
Humulus lupulus	CC(C)=CCC1=C(O)C(C(=O)CCC(C)C)=C(O)C(O)(CC=C(C)C)C1=O	-6.64
Scutellaria baicalensis	COc1c(OC)c(O)c2c(=O)cc(-c3c(O)cccc3O[C@@H]3OC(CO)[C@@H](O)[C@H](O)C3O)oc2c1OC	-6.62
Humulus lupulus	CC(C)=CCc1c(O)c(CC=C(C)C)c2c(c1O)C(=O)C[C@@H](c1ccc(O)cc1)O2	-6.62
Humulus lupulus	Nc1ncnc2c1ncn2[C@@H]1O[C@H](COP(=O)(O)OP(=O)(O)OP(=O)(O)O)[C@H](O)C1O	-6.61
Humulus lupulus	COc1cc(OC)c2c(c1)OC(c1ccc(O)cc1)CC2=O	-6.6
Humulus lupulus	CC(C)=CCNc1ncnc2c1ncn2[C@@H]1O[C@H](COP(=O)(O)O)[C@@H](O)[C@H]1O	-6.6
Magnolia officinalis	O[C@@H]1c2ccccc2-c2c3c(cc4c2[C@@H]1NCC4)OCO3	-6.6
Scutellaria baicalensis	COc1c(O)cc2c(c1O)C(=O)CC(c1ccccc1)O2	-6.59
Humulus lupulus	CC(C)=CCc1c(O)cc(O)c(C(=O)/C=C/c2ccc(O)cc2)c1O	-6.59
Humulus lupulus	CC1=C2C[C@H](C(C)(C)O)CC[C@@]2(C)CCC1	-6.59
Scutellaria baicalensis	O=C1CC(c2ccc(O)cc2)Oc2c(O)c(O)cc(O)c21	-6.58
Humulus lupulus	Oc1cc(O)c2c(c1)OC(c1ccc(O)c(O)c1)C(O)C2O	-6.58
Humulus lupulus	COc1cc(O)c(CC=C(C)C)c(O)c1C(=O)/C=C/c1ccc(O)cc1	-6.57
Magnolia officinalis	CC1=C2C[C@H](C(C)(C)O)CC[C@@]2(C)CCC1	-6.56
Valeriana officinalis	COc1cc(C2OCC(C(O)c3ccc(OC4OC(CO)C(O)C(O)C4O)c(OC)c3)C2CO)ccc1O	-6.55
Melissa officinalis	CC(C)(COP(=O)(O)OP(=O)(O)OCC1OC(n2cnc3c(N)ncnc32)C(O)C1OP(=O)(O)O)C(O)C(=O)NCCC(=O)NCCSC(=O)/C=C/c1ccc(O)cc1	-6.52
Humulus lupulus	CC(C)=CCc1c(O)ccc2c1OC(c1ccc(O)cc1)CC2=O	-6.5
Scutellaria baicalensis	COc1c(O[C@@H]2OC(CO)[C@@H](O)[C@H](O)C2O)cc(O)c2c(=O)cc(-c3ccccc3)oc12	-6.48
Humulus lupulus	C/C(=CCNc1ncnc2c1ncn2[C@@H]1O[C@H](CO)[C@H](O)C1O)CO[C@H]1OC(CO)[C@@H](O)C(O)C1O	-6.48
Humulus lupulus	CC(C)CC(=O)C1=C(O)C2(O)C(=O)CC3C(C)(C)C(C(C)(C)O)CC32C1=O	-6.47
Magnolia officinalis	COc1ccc2c(c1O)-c1c(O)c(OC)cc3c1[C@H](C2)N(C)CC3	-6.47
Magnolia officinalis	COc1ccc(C[C@@H]2c3cc(O)c(OC)cc3CCN2C)cc1	-6.46
Magnolia officinalis	COc1ccc2c(c1O)-c1c3c(cc4c1C(C2)[N+](C)(C)CC4)OCO3	-6.46
Scutellaria baicalensis	COc1cccc(O[C@@H]2O[C@@H](C(=O)O)[C@@H](O)C(O)C2O)c1-c1cc(=O)c2c(O)cc(OC)c(OC)c2o1	-6.45
Humulus lupulus	O=C(/C=C/c1ccc(O)c(O)c1)O[C@@H]1C[C@](O)(C(=O)O)C[C@@H](O)[C@H]1O	-6.44
Humulus lupulus	Oc1cc(O)c2c(c1)O[C@H](c1ccc(O)c(O)c1)[C@H](O)C2	-6.44
Magnolia officinalis	O=CC=Cc1ccc(O)c(-c2cc(CC(O)CO)ccc2O)c1	-6.44
Magnolia officinalis	C=CCc1ccc(O)c(-c2cc(CC=C)cc(C3C=C(C)CCC3C(C)C)c2O)c1	-6.44
Magnolia officinalis	COc1cc2c(cc1O)C(Cc1ccc(O)cc1)[N+](C)(C)C=C2	-6.44
Scutellaria baicalensis	COc1c(O)cc(O[C@@H]2OC(CO)[C@@H](O)[C@H](O)C2O)c2c(=O)cc(-c3ccccc3)oc12	-6.43
Scutellaria baicalensis	O=C1CC(c2c(O)cccc2O)Oc2cc(O)cc(O)c21	-6.43
Humulus lupulus	CC(C)CC(=O)C1=C(O)C2(O)C(=O)CC3C(C)(C)C(C(C)(C)O)CC32C1=O	-6.43
Humulus lupulus	OCC1O[C@H](O[C@@H]2C(CO)O[C@@H](O)C(O)[C@H]2O)C(O)[C@@H](O)[C@@H]1O	-6.42
Magnolia officinalis	COc1cc2c(cc1O)[C@@H](Cc1ccc(O)cc1)[N+](C)(C)CC2	-6.42
Magnolia officinalis	COc1cc(C2OCC3C(c4cc(OC)c(OC5OC(CO)C(O)C(O)C5O)c(OC)c4)OCC23)cc(OC)c1O	-6.42
Humulus lupulus	CC(=O)SCCNC(=O)CCNC(=O)C(O)C(C)(C)COP(=O)(O)OP(=O)(O)OCC1OC(n2cnc3c(N)ncnc32)C(O)C1OP(=O)(O)O	-6.41
Valeriana officinalis	COc1cc(C2OCC3(O)C(c4ccc(OC5OC(CO)C(O)C(O)C5O)c(OC)c4)OCC23)ccc1O	-6.4
Humulus lupulus	CC(C)=CC1C(=O)C2(O)C(O)=C(C(=O)C(C)C)C(=O)C2(O)CC1C(C)(C)O	-6.4
Humulus lupulus	CC(C)=CC1OC(C)(C)C2CC3(O)C(=O)C(C(=O)C(C)C)=C(O)C3(O)C12O	-6.38
Magnolia officinalis	Oc1ccc(C[C@@H]2NCCc3cc(O)c(O)cc32)cc1	-6.38
Magnolia officinalis	C=CCc1cc(-c2cc([C@@H](O)[C@@H](O)CO)ccc2O)ccc1O	-6.37
Scutellaria baicalensis	O=c1cc(-c2c(O)cccc2O)oc2cc(O)cc(O)c12	-6.36
Humulus lupulus	C[C@](CO)(OP(=O)(O)O)[C@H](O)COP(=O)(O)OP(=O)(O)OC[C@H]1O[C@@H](n2ccc(N)nc2=O)[C@H](O)[C@@H]1O	-6.36
Magnolia officinalis	C=CCc1cc(-c2cc(/C=C/C=O)ccc2O)ccc1O	-6.36
Magnolia officinalis	C=CCc1ccc(Oc2cc(/C=C/C=O)cc(O)c2O)cc1	-6.35
Magnolia officinalis	COc1cc2c3c(c1O)-c1ccccc1C[C@@H]3N(C)CC2	-6.35
Humulus lupulus	CC(C)=CCC1=C2OC(C)(C)C=CC2(CC=C(C)C)C(=O)C(C(=O)C(C)C)=C1O	-6.34
Scutellaria baicalensis	COc1c(O)cc2oc(-c3ccccc3O)cc(=O)c2c1O	-6.33
Scutellaria baicalensis	O=C1C[C@@H](c2ccc(O)c(O)c2)Oc2cc(O)cc(O)c21	-6.32
Humulus lupulus	CC(C)C(=O)C1=C(O)C2(O)C(=O)CC3C(C)(C)C(C(C)(C)O)CC32C1=O	-6.32
Valeriana officinalis	CC(C)CC(=O)O[C@@H]1OC=C(CO[C@@H]2OC(CO)[C@@H](O)C(O)[C@@H]2O)[C@H]2C[C@H](O)[C@H](CO)[C@@H]12	-6.31
Humulus lupulus	CC(C)=CCC1(CC=C(C)C)C(=O)C(O)=C(O)C1=O	-6.31
Humulus lupulus	CC(C)=CCc1c(O)cc(O)c2c1OC(c1ccc(O)cc1)CC2=O	-6.31
Magnolia officinalis	C=CCc1cc(-c2cc([C@H](O)[C@@H](O)CO)ccc2O)ccc1O	-6.31
Humulus lupulus	CC(C)=CC1OC(C)(C)C2CC3(O)C(=O)C(C(=O)CC(C)C)=C(O)C3(O)C12O	-6.3
Magnolia officinalis	COc1cc2c(cc1O)C[C@H]1c3c(cc(OC)c(OC)c3-2)CC[N+]1(C)C	-6.3
Valeriana officinalis	COc1cc(C2OCC(O)(Cc3ccc(OC4OC(CO)C(O)C(O)C4O)c(OC)c3)C2CO)ccc1O	-6.29
Magnolia officinalis	C=CCc1ccc(O)c(-c2cc(C[C@@H](O)CO)ccc2O)c1	-6.29

Humulus lupulus	CCC(C)C(=O)C1=C(O)C2(O)C(=O)CC3C(C)(C)C(C(C)(C)O)CC32C1=O	-6.28
Humulus lupulus	CCC(C)C(=O)C1=C(O)C(CC=C(C)C)(CC=C(C)C)C(=O)C(CC=C(C)C)(CC=C(C)C)C1=O	-6.28
Humulus lupulus	CCC(C)C(=O)C1=C(O)C(O)C(O)(CC=C(C)C)C1=O	-6.28
Magnolia officinalis	C=CCc1cc(-c2cc(O)ccc2O)ccc1O	-6.28
Valeriana officinalis	CC(C)CC(=O)O[C@@H]1OC=C(CO)[C@H]2[C@@H]3O[C@@H]3[C@@](O)(CO)[C@@H]12	-6.27
Humulus lupulus	CC(C)=CCC(=O)[C@]1(O)C(O)=C(C(=O)CC(C)C)C(=O)[C@H]1CC=C(C)C	-6.27
Magnolia officinalis	COc1cccc2c1-c1c3c(cc4c1C(C2)NCC4)OCO3	-6.27
Valeriana officinalis	O=c1c(O)c(-c2ccc(O)c(O)c2)oc2cc(O)cc(O)c12	-6.26
Scutellaria baicalensis	COc1cc(O)c2c(=O)cc(-c3ccccc3O)oc2c1O	-6.26
Humulus lupulus	O=c1c(O[C@@H]2O[C@H](CO)[C@@H](O)C(O)C2O)c(-c2ccc(O)cc2)oc2cc(O)cc(O)c12	-6.26
Humulus lupulus	O=c1c(O)c(-c2ccc(O)c(O)c2)oc2cc(O)cc(O)c12	-6.26
Magnolia officinalis	CCCCCCC(C)(C)c1ccc([C@@H]2C[C@H](O)CC[C@H]2CCCO)c(O)c1	-6.26
Humulus lupulus	CC(C)CC(=O)c1c(O)cc(O)cc1O	-6.25
Humulus lupulus	O=C1CC(c2ccccc2)Oc2ccccc21	-6.25
Magnolia officinalis	C=CCc1ccc(O)c(-c2ccc3c(c2)[C@H](O)[C@@H](CO)O3)c1	-6.25
Scutellaria baicalensis	COc1c(O)cc2oc(-c3ccccc3)cc(=O)c2c1O	-6.24
Melissa officinalis	C[C@H]1O[C@@H](OC(=O)[C@]23CC[C@@](C)(CO)C[C@H]2C2=CC[C@@H]4[C@@]5(C)C[C@@H](O)[C@H](O)[C@@](C)(CO)[C@@H]5CC[C@@]4(C)[C@]2(C)C[C@H]3O)[C@H](O)[C@@H](O)[C@H]1O	-6.24
Piper methysticum	COc1cc(O)c(C(=O)/C=C/c2ccc(O)cc2)c(OC)c1	-6.23
Humulus lupulus	CC(C)=CCC1=C2OC(C(C)(C)O)CC2(CC=C(C)C)C(=O)C(C(=O)C(C)C)=C1O	-6.22
Scutellaria baicalensis	COc1c(O)ccc(O)c1-c1cc(=O)c2c(O)cc(O)c(OC)c2o1	-6.2
Humulus lupulus	CCC(C)C(=O)SCCNC(=O)CCNC(=O)C(O)C(C)(C)COP(=O)(O)OP(=O)(O)OCC1OC(n2cnc3c(N)ncnc32)C(O)C1OP(=O)(O)O	-6.2
Magnolia officinalis	C=CCc1ccc(O)c(-c2cc(/C=C/CO)ccc2O)c1	-6.2
Melissa officinalis	O=c1cc(-c2ccc(O)c(O)c2)oc2cc(O)cc(O)c12	-6.19
Humulus lupulus	CCC(C)C(=O)C1=C(O)C2(O)C(=O)CC3C(C)(C)C(C(C)(C)O)CC32C1=O	-6.19
Humulus lupulus	C=C(C)[C@@H]1CC[C@]2(C)CC[C@]3(C)[C@H](CC[C@@H]4[C@@]5(C)CC[C@H](O)C(C)(C)[C@@H]5CC[C@]43C)[C@@H]12	-6.19
Scutellaria baicalensis	COc1ccc(O)c(O)c1-c1cc(=O)c2c(O)cc(O)c(OC)c2o1	-6.18
Humulus lupulus	CC(C)=CCC1(O)C(=O)C(C(=O)C(C)C)=C(O)C1(O)C(=O)C=CC(C)(C)O	-6.18
Humulus lupulus	COc1cc(O)c(C(=O)/C=C/c2ccc(O)cc2)c(O)c1CC=C(C)C	-6.18
Piper methysticum	COc1cc(O)c(C(=O)CCc2ccc(O)cc2)c(OC)c1	-6.17
Scutellaria baicalensis	COc1cc2oc(-c3c(O)cccc3O[C@@H]3OC(CO)[C@@H](O)[C@H](O)C3O)cc(=O)c2c(O)c1OC	-6.17
Humulus lupulus	CC1(C)CC[C@]2(C)CC[C@]3(C)C(C=C[C@@H]4[C@@]5(C)CC[C@H](O)C(C)(C)[C@@H]5CC[C@]43C)[C@@H]2C1	-6.17
Piper methysticum	COc1cc(O)c(C(=O)/C=C/c2ccccc2)c(OC)c1	-6.16
Melissa officinalis	CC1=C[C@@H]2[C@@H](CC1)[C@](C)(O)CC[C@H]2C(C)C	-6.16
Humulus lupulus	CC(C)=CCC1(O)C(=O)C(C(=O)CC(C)C)=C(O)C1O	-6.16
Humulus lupulus	CC(C)=CCC(=O)[C@@]1(O)C(O)=C(C(=O)CC(C)C)C(=O)[C@H]1CC=C(C)C	-6.16
Magnolia officinalis	c1ccc2c(c1)C[C@H]1NCCc3cc4c(c-2c31)OCO4	-6.16
Magnolia officinalis	C=CCc1ccc(O)c(-c2cc(C(O)C(O)CO)ccc2O)c1	-6.16
Magnolia officinalis	C=CCc1ccc(O)c(-c2cc(CC(O)CO)ccc2O)c1	-6.16
Scutellaria baicalensis	O=c1cc(-c2ccccc2)oc2c(O)c(O)cc(O)c12	-6.15
Scutellaria baicalensis	O=c1cc(-c2ccccc2O)oc2cc(O)cc(O)c12	-6.15
Scutellaria baicalensis	COc1c(O)cc(O)c2c(=O)cc(-c3ccccc3O)oc12	-6.15
Humulus lupulus	CC(C)=CC1OC(C)(C)C2CC3(O)C(=O)C(C(=O)C(C)C)=C(O)C3(O)C12O	-6.15
Magnolia officinalis	C=CCc1cc(-c2cc(C(OC)C(O)CO)ccc2O)ccc1O	-6.15
Humulus lupulus	CC1CCC2(C)CCC3(C)C(=CC=C4C5(C)CCC(O)C(C)(C)C5CCC43C)C2C1C	-6.14
Humulus lupulus	Oc1cc(O)c2c(c1)O[C@H](c1ccc(O)c(O)c1)[C@@H](O)C2	-6.14
Magnolia officinalis	C=CCc1ccc(O)c(-c2cc(O)ccc2O)c1	-6.14
Valeriana officinalis	C=C(C)[C@@H]1CC[C@@]2(C)CCC[C@@](C)(O)[C@@H]2C1	-6.13
Scutellaria baicalensis	COc1cccc(O)c1-c1cc(=O)c2c(O)cc(O)c(OC)c2o1	-6.13
Humulus lupulus	CC(C)=CCC1=C2OC(C(C)(C)O)CC2(CC=C(C)C)C(=O)C(C(=O)CC(C)C)=C1O	-6.13
Valeriana officinalis	O=c1oc2c(O)c(O)cc3c(=O)oc4c(O)c(O)cc1c4c23	-6.12
Piper methysticum	COc1cc(O)c(C(=O)/C=C/c2ccccc2)c(OC)c1	-6.12
Humulus lupulus	CC(C)=CCC1=C(O)C(O)(CC=C(C)C)C(=O)C(C(=O)CC(C)C)=C1O	-6.12
Humulus lupulus	CC(C)=CCC1(O)C(=O)C(C(=O)C(C)C)=C(O)C1O	-6.12
Humulus lupulus	CC(C)=CCC1(O)C(=O)C(C(=O)C(C)C)=C(O)C1(O)C(=O)C=CC(C)(C)O	-6.12
Humulus lupulus	CC(C)=CCC1(CC=C(C)C)C(=O)C2=C(OC(C(C)(C)O)C2)C(C(=O)CC(C)C)=C1O	-6.12
Magnolia officinalis	COc1ccc(CC2c3cc(OC)c(OC)cc3CC[N+]2(C)C)cc1O	-6.12
Valeriana officinalis	CC1=CCC[C@]2(C)CC[C@@H](C(C)(C)O)CC12	-6.11
Scutellaria baicalensis	O=c1c(O)c(-c2c(O)cccc2O)oc2cc(O)cc(O)c12	-6.11
Humulus lupulus	CC(C)=CCC1(CC=C(C)C)C(=O)C2=C(OC(C(C)(C)O)C2)C(C(=O)C(C)C)=C1O	-6.11
Humulus lupulus	CC(C)(COP(=O)(O)OP(=O)(O)OCC1OC(n2cnc3c(N)ncnc32)C(O)C1OP(=O)(O)O)C(O)C(=O)NCCC(=O)NCCSC(=O)CC(=O)O	-6.11
Humulus lupulus	CC1=CCC[C@]2(C)CC[C@@H](C(C)(C)O)CC12	-6.11
Magnolia officinalis	CC1=CCC[C@]2(C)CC[C@@H](C(C)(C)O)CC12	-6.11
Magnolia officinalis	C=CCc1ccc(O)c(-c2cc(C(O)C(O)CO)ccc2O)c1	-6.1
Valeriana officinalis	CC1=CC(=O)[C@H]2[C@H]3[C@@H]1[C@]2(C)CCCC3(C)C	-6.09
Magnolia officinalis	C=CCc1ccc(O)c(-c2cc(C=O)ccc2O)c1	-6.09
Scutellaria baicalensis	O=c1cc(-c2ccccc2)oc2cc(O)c(O)c(O)c12	-6.08
Scutellaria baicalensis	O=C1c2c(O)cc(O)cc2OC(c2c(O)cccc2O)C1O	-6.08
Humulus lupulus	CC(C)=CCC1=C(O)C(CC=C(C)C)(CC=C(C)C)C(=O)C(C(=O)CCC(C)C)=C1O	-6.08
Humulus lupulus	CCC(C)C(=O)c1c(O)cc(O)cc1O[C@@H]1O[C@H](CO)[C@@H](O)[C@H](O)[C@H]1O	-6.08
Humulus lupulus	COc1cc(O)c2c(c1CC=C(C)C)O[C@H](c1ccc(O)cc1)CC2=O	-6.08
Magnolia officinalis	C=C1CCC[C@]2(C)CC[C@@H](C(C)(C)O)C[C@@H]12	-6.08
Scutellaria baicalensis	OCC1O[C@H](O[C@]2(CO)O[C@H](CO)C(O)[C@H]2O)C(O)[C@@H](O)[C@@H]1O	-6.07
Humulus lupulus	CC(C)C(=O)c1c(O)cc(O)cc1O	-6.07
Valeriana officinalis	C=C1CCC[C@]2(C)CC[C@@H](C(C)(C)O)C[C@@H]12	-6.06
Piper methysticum	COc1ccc(/C=C/C(=O)c2c(O)cc(OC)cc2OC)cc1	-6.06
Piper methysticum	CC(=O)OC1C=CC(=O)N(C(=O)CCc2ccccc2)C1	-6.06
Scutellaria baicalensis	O=c1c(O)c(-c2cc(O)ccc2O)oc2cc(O)cc(O)c12	-6.06
Scutellaria baicalensis	OC[C@H]1O[C@@H](OCCc2ccc(O)cc2)[C@H](O)[C@@H](O)[C@@H]1O	-6.06
Humulus lupulus	CCC(C)C(=O)C1=C(O)C(O)(C(=O)C=CC(C)(C)O)C(O)(CC=C(C)C)C1=O	-6.06
Humulus lupulus	CCC(C)CC(=O)C1=C(O)C(CC=C(C)C)=C(O)[C@](O)(CC=C(C)C)C1=O	-6.06
Piper methysticum	CC[C@H](/C=C/[C@@H](C)[C@H]1CC[C@H]2[C@@H]3CC=C4C[C@@H](O)CC[C@]4(C)[C@H]3CC[C@]12C)C(C)C	-6.05
Piper methysticum	COC1=CC(=O)O[C@@H](CCc2ccc(OC)c(O)c2)C1	-6.05
Humulus lupulus	CC(C)=CCC1(CC=C(C)C)C(=O)C(=O)C(C(=O)C(C)C)C1=O	-6.05
Scutellaria baicalensis	COc1c(O)cc2oc(-c3ccc(O)cc3)cc(=O)c2c1O	-6.04
Magnolia officinalis	COc1cc2c(cc1O)CC[N+](C)(C)[C@@H]2Cc1ccc(O)cc1	-6.04
Scutellaria baicalensis	COc1cccc(O)c1-c1cc(=O)c2c(O)cc(OC)c(OC)c2o1	-6.03
Humulus lupulus	CC(C)=CCC1(O)C(=O)C(C(=O)CC(C)C)=C(O)C1(O)C(=O)C=CC(C)(C)O	-6.03
Humulus lupulus	CC(C)C(=O)SCCNC(=O)CCNC(=O)[C@H](O)C(C)(C)COP(=O)(O)OP(=O)(O)OC[C@H]1O[C@@H](n2cnc3c(N)ncnc32)[C@H](O)[C@@H]1OP(=O)(O)O	-6.03
Humulus lupulus	CCC(C)C(=O)C1=C(O)C(O)(C(=O)CC=C(C)C)C(CC=C(C)C)C1=O	-6.02

Humulus lupulus	Nc1ncnc2c1ncn2[C@@H]1O[C@H](COP(=O)(O)O)C(O)[C@@H]1O	-6.02
Magnolia officinalis	COc1cc2c3c(c1O)-c1cc(O)c(O)cc1CC3[N+](C)(C)CC2	-6.02
Humulus lupulus	CC(C)=CCC(=O)C1(O)C(O)=C(C(=O)C(C)C)C(=O)C1CC=C(C)C	-6.01
Magnolia officinalis	C=CCc1cc(-c2cc(C=O)ccc2O)ccc1O	-6
Valeriana officinalis	CC1=CC[C@@]23C[C@@H]1[C@@](C)(CO)[C@@H]2CC[C@H]3C	-5.99
Valeriana officinalis	CC1(O)CCC2C(C3C1CCC3(O)CO)C2(C)C	-5.99
Piper methysticum	COC1=CC(=O)O[C@@H](/C=C/c2ccccc2)C1O	-5.99
Humulus lupulus	CC(C)=CCC(=O)C1(O)C(O)=C(C(=O)C(C)C)C(=O)C1CC=C(C)C	-5.99
Humulus lupulus	CC(C)=CCC1=C(O)C(C(=O)C(C)C)=C(O)C(CC=C(C)C)(CC=C(C)C)C1=O	-5.99
Valeriana officinalis	CC(C)CC(=O)O[C@@H]1OC=C(CO[C@@H]2OC(CO)[C@@H](O)C(O)[C@@H]2O)[C@H]2[C@@H]3O[C@@H]3[C@@](O)(CO)[C@@H]12	-5.98
Piper methysticum	COc1ccc(/C=C/C(=O)c2c(O)cc(OC)cc2OC)cc1	-5.98
Scutellaria baicalensis	COc1cccc(O)c1-c1cc(=O)c2c(O)cc(O)cc2o1	-5.98
Magnolia officinalis	COc1cc(C(O)C(CO)Oc2ccc(CCCO)cc2OC)ccc1O	-5.98
Valeriana officinalis	CC(=O)O[C@H]1C[C@@]2(C)CCCC(=O)[C@@]2(C)C[C@H]1C(C)C	-5.97
Scutellaria baicalensis	O=c1cc(-c2ccc(O)cc2)oc2cc(O)cc(O)c12	-5.97
Humulus lupulus	COc1cc(O)c(CC=C(C)C)c2c1C(=O)CC(c1ccc(O)cc1)O2	-5.97
Valeriana officinalis	COc1cc([C[C@@]2(O)CO[C@H](c3ccc(O)c(OC)c3)[C@H]2CO)ccc1O	-5.96
Valeriana officinalis	CC(=CC1CCC(C)C2CCC(C)=C12)COC(=O)CC(C)C	-5.96
Scutellaria baicalensis	COc1c(OC)c(O)c2c(=O)cc(-c3ccccc3)oc2c1O	-5.95
Humulus lupulus	CCCC(=O)c1c(O)cc(O)c(C)c1O	-5.95
Humulus lupulus	CC(C)=CCC1=C(O)[C@](O)(CC=C(C)C)C(=O)C(C(=O)CC(C)C)=C1O	-5.95
Humulus lupulus	CSc1nc(NC/C=C(C)CO)c2ncn([C@@H]3O[C@H](CO)C(O)[C@@H]3O)c2n1	-5.95
Scutellaria baicalensis	O=C1C[C@@H](c2ccccc2)Oc2cc(O)cc(O)c21	-5.94
Humulus lupulus	CC(C)=CCC(=O)C1(O)C(=O)C(C(=O)CC(C)C)=C(O)C1(O)CC=C(C)C	-5.94
Humulus lupulus	CC(C)=CCC1=C(O)C(C(=O)CC(C)C)=C(O)C(CC=C(C)C)(CC=C(C)C)C1=O	-5.94
Humulus lupulus	CC(C)CC(=O)c1c(O)cc(O)cc1O[C@@H]1O[C@H](CO)[C@@H](O)[C@H](O)[C@H]1O	-5.93
Valeriana officinalis	C[C@H]1CCc2c(CO)c[n+](CCc3ccc(O)cc3)cc21	-5.92
Humulus lupulus	CC(C)C(=O)c1c(O)ccc2c1CC(C(C)(C)O)O2	-5.92
Valeriana officinalis	CC(C)=CCC/C(C)=C/CC/C(C)=C/CC/C(C)=C/COP(=O)(O)OP(=O)(O)O	-5.91
Valeriana officinalis	Cc1cccc(C(C)c2ccccc2)c1O	-5.91
Scutellaria baicalensis	COc1c(O)cc(O)c2c(=O)cc(-c3ccc(O)cc3)oc12	-5.91
Scutellaria baicalensis	COc1c(OC)c(O)c2c(=O)cc(-c3cc(O)ccc3O)oc2c1OC	-5.91
Humulus lupulus	CCC(C)C(=O)C1=C(O)C(CC=C(C)C)=C(O)[C@](O)(CC=C(C)C)C1=O	-5.91
Humulus lupulus	COc1cc(OC)c(C(=O)/C=C/c2ccc(O)cc2)c(O)c1CC=C(C)C	-5.91
Magnolia officinalis	C=CCc1ccc(Oc2cc(C=O)cc(O)c2O)cc1	-5.91
Magnolia officinalis	C=CCc1ccc(Oc2cc(CC=C)ccc2O)cc1	-5.9
Valeriana officinalis	CC1=CC[C@@H](C(C)C)c2cc(C)ccc21	-5.89
Scutellaria baicalensis	CC(C)[C@H](C)CC[C@@H](C)[C@H]1CC[C@H]2[C@@H]3CC=C4C[C@@H](O)CC[C@]4(C)[C@H]3CC[C@]12C	-5.89
Scutellaria baicalensis	COc1ccc(-c2cc(=O)c3c(O)cc(O)c(O)c3o2)cc1	-5.89
Valeriana officinalis	COc1cc(C2OC[C@]3(O)[C@@H](c4ccc(O)c(OC)c4)OC[C@H]23)ccc1O	-5.88
Humulus lupulus	C=C(C)[C@@H]1CC[C@@]2(C)CCC[C@]3(CO3)[C@@H]2C1	-5.88
Magnolia officinalis	C=CCc1ccc(O)c(-c2cc(C(O)C(O)CO)ccc2O)c1	-5.88
Valeriana officinalis	Cc1c[n+](CCc2ccc(O)cc2)cc2c1CC[C@@H]2C	-5.87
Valeriana officinalis	CC(C)=CCC/C(C)=C/CC/C(C)=C/COP(=O)(O)OP(=O)(O)O	-5.87
Valeriana officinalis	C=C(C)[C@@H]1CC[C@@]2(C)CCC[C@]3(CO3)[C@@H]2C1	-5.87
Valeriana officinalis	CC(=O)OCC(C)=CC1CCC(C)C2CCC3(C)OC123	-5.87
Humulus lupulus	CC(C)=CCC1=C2OC(C)(C)C=CC2(CC=C(C)C)C(=O)C(C(=O)CC(C)C)=C1O	-5.87
Humulus lupulus	OCC1O[C@H](O[C@]2(CO)O[C@H](CO)C(O)[C@H]2O)C(O)[C@@H](O)[C@@H]1O	-5.87
Magnolia officinalis	COc1cc2c(cc1O)[C@H](Cc1ccc(O)cc1)N(C)CC2	-5.87
Valeriana officinalis	CC(C)C1CCC2C(=O)CCC(C)C2(C)C1	-5.86
Scutellaria baicalensis	O=c1cc(-c2ccc(O)cc2)oc2c(O)c(O)cc(O)c12	-5.86
Scutellaria baicalensis	O=c1cc(-c2c(O)cccc2O)oc2cc(O)cc(O)c12	-5.86
Humulus lupulus	O=C(O)/C=C/c1ccccc1O	-5.86
Magnolia officinalis	C=CCc1ccc(O)c(-c2ccc(O)c(CC=C)c2)c1	-5.86
Humulus lupulus	CC(C)=CC1C(=O)C2(O)C(O)=C(C(=O)C(C)C)C(=O)C2(O)CC1C(C)(C)O	-5.85
Magnolia officinalis	COc1cc(C=CC=O)cc(OC)c1OC1OC(CO)C(O)C(O)C1O	-5.85
Magnolia officinalis	C=CCc1ccc(O)c(-c2cc(CC=C)ccc2OC2C3CCCC2(C)C3(C)C)c1	-5.85
Piper methysticum	COC1=CC(=O)O[C@@H](CCc2ccc3c(c2)OCO3)C1	-5.84
Scutellaria baicalensis	COc1cc(O)c2c(=O)cc(-c3ccccc3)oc2c1OC	-5.83
Scutellaria baicalensis	O=c1cc(-c2cc(O)ccc2O)oc2cc(O)cc(O)c12	-5.83
Humulus lupulus	Cc1ccc2c(c1)[C@H](C(C)C)CC[C@@H]2C	-5.83
Humulus lupulus	CSc1nc(NCC=C(C)C)c2ncn(C3OC(CO)C(O)C3O)c2n1	-5.83
Valeriana officinalis	CC(=O)OC[C@@]1(O)[C@H]2[C@H](OC(=O)CC(C)C)OC=C(COC(=O)C(OC(=O)CC(C)C)CC(C)C)[C@@]2(O)C[C@@H]1O	-5.82
Humulus lupulus	CC(C)=CCC1(O)C(=O)C(C(=O)CC(C)C)=C(O)C1(O)C(=O)C=CC(C)(C)O	-5.82
Magnolia officinalis	C=C1CC[C@H]2[C@@H]1[C@H]1[C@@H](CC[C@H]2C)C1(C)C	-5.82
Magnolia officinalis	C=CCc1ccc(O)c(-c2ccc(O)c(CC=C)c2)c1	-5.82
Humulus lupulus	CCC(C)C(=O)C1=C(O)C(O)(C(=O)CC=C(C)C)C(CC=C(C)C)C1=O	-5.81
Magnolia officinalis	C=CCc1cc(-c2cc(CC(O)CO)ccc2O)ccc1O	-5.81
Magnolia officinalis	CC1=CCC[C@@H](C)[C@]12CC[C@@H](C(C)(C)O)C2	-5.81
Scutellaria baicalensis	COc1c(O)cc(O)c2c(=O)cc(-c3ccccc3)oc12	-5.8
Scutellaria baicalensis	O=c1cc(-c2ccc(O)cc2)oc2cc(O)c(O)c(O)c12	-5.8
Magnolia officinalis	COc1ccc2c(c1O)C(Cc1ccc(O)cc1)[N+](C)(C)CC2	-5.8
Humulus lupulus	CC(C)=CCC1=C(O)C(O)(CC=C(C)C)C(=O)C(C(=O)C(C)C)=C1O	-5.79
Humulus lupulus	CCC(C)C(=O)C1=C(O)C(CC=C(C)C)=C(O)C(CC=C(C)C)(CC=C(C)C)C1=O	-5.79
Magnolia officinalis	COc1ccc2c(c1O)C(Cc1ccc(O)cc1)[N+](C)(C)CC2	-5.79
Magnolia officinalis	CC1CCC2C(C1)C(C(C)C)CCC2(C)O	-5.79
Humulus lupulus	O=C(O)Cc1cccc2ccccc12	-5.78
Valeriana officinalis	CC1CCC(O)C2(O)CCC(C(C)(C)O)CC12C	-5.77
Valeriana officinalis	C[C@@H]1C[C@@H](O)[C@@H]2[C@@H]1C[C@H]1CC[C@]2(C)OC1(C)C	-5.76
Valeriana officinalis	CC(=O)OC1CC2C(CC3CCC2(C)OC3(C)C)C1C	-5.76
Magnolia officinalis	C=CC1CCC(=C)C2CCC(CO)CC12	-5.76
Humulus lupulus	CC(C)C(=O)C1=C(O)C2(O)C(=O)CC3C(C)(C)C(C(C)(C)O)CC32C1=O	-5.75
Valeriana officinalis	CC1CCC2C1CC1CCC2(C)OC1(C)CO	-5.74
Humulus lupulus	COc1cc(OC)c2c(c1CC=C(C)C)O[C@H](c1ccc(O)cc1)CC2=O	-5.74
Magnolia officinalis	COc1cc(C2OCC3C(c4cc(OC)c(O)c(OC)c4)OCC23)cc(OC)c1O	-5.74
Magnolia officinalis	COc1ccc2c(c1O)-c1c(O)c(OC)cc3c1C(C2)[N+](C)(C)CC3=O	-5.74
Valeriana officinalis	C=C1CCC(C(C)C)=C[C@@H]2[C@@H]1CC[C@@]2(C)O	-5.73
Valeriana officinalis	CC(=O)OC1CC2(C)OC(C)(C)C1CC1C(C)CC(OC(C)=O)C12	-5.72
Valeriana officinalis	COc1cc(C2OCC3(O)C(c4ccc(O)c(OC)c4)OCC23O)ccc1O	-5.72
Piper methysticum	COc1ccc(CCC(=O)c2c(O)cc(OC)cc2OC)cc1	-5.72
Humulus lupulus	CC(C)=CCc1c(O)c2c(c(C(=O)/C=C/c3ccc(O)cc3)c1O)OC(C)(C)C=C2	-5.71

Scutellaria baicalensis	O=C1c2c(O)cc(O)cc2O[C@H](c2c(O)cccc2O)[C@H]1O	-5.7
Humulus lupulus	C[C@@H]1CC[C@H]2[C@@H]1[C@H]1[C@@H](CC[C@@]2(C)O)C1(C)C	-5.7
Valeriana officinalis	C[C@@H]1C[C@@H](O)[C@@H]2[C@@H]1CC1[C@@H](O)C[C@]2(C)OC1(C)C	-5.69
Humulus lupulus	CC(C)=CCNc1ncnc2c1ncn2[C@@H]1O[C@H](CO)C(O)[C@@H]1O	-5.69
Valeriana officinalis	CC(C)=CCC[C@@](C)(O)[C@@H]1CC=C(C)CC1	-5.68
Humulus lupulus	CCC(=O)C1=C(O)C(O)(CC=C(C)C)C(=O)C(CC=C(C)C)=C1O	-5.68
Humulus lupulus	Cc1ccc2c(C)ccc(C(C)C)c2c1	-5.68
Humulus lupulus	CC(C)=CCC/C(C)=C/CC/C(C)=C/COP(=O)(O)OP(=O)(O)O	-5.68
Humulus lupulus	C=C(C)[C@@H]1CC[C@@]2(C)CCCC(=C)[C@@H]2C1	-5.67
Passiflora incarnata	Cc1nccc2c1[nH]c1cc(O)ccc12	-5.66
Humulus lupulus	C=C(C)[C@@H]1CC[C@@]2(C)CCC=C(C)[C@@H]2C1	-5.66
Magnolia officinalis	COc1cc(C(O)C(CO)Oc2ccc(CCCO)cc2OC)ccc1O	-5.66
Humulus lupulus	CC(C)=CCc1c(O)c2c(c(C(=O)CC(C)C)c1O)OC(C(C)(C)O)C2	-5.65
Valeriana officinalis	CC1CCC(=O)C2CCC(C(C)(C)O)CC12C	-5.64
Scutellaria baicalensis	CC[C@H](CC[C@@H](C)[C@H]1CC[C@H]2[C@@H]3CC=C4C[C@@H](O)CC[C@]4(C)[C@H]3CC[C@]12C)C(C)C	-5.64
Humulus lupulus	COc1cc2c(c(O)c1CC=C(C)C)C(=O)C[C@@H](c1ccc(O)cc1)O2	-5.64
Humulus lupulus	O=c1c(O)c(-c2ccc(O)cc2)oc2cc(O)cc(O)c12	-5.64
Magnolia officinalis	COc1cc(/C=C/CO)cc(OC)c1O[C@@H]1OC(CO)[C@@H](O)[C@H](O)C1O	-5.63
Humulus lupulus	CC(C)=CC1C(=O)C2(O)C(O)=C(C(=O)CC(C)C)C(=O)C2(O)CC1C(C)(C)O	-5.62
Humulus lupulus	CC1(C)CC[C@]2(C)CC[C@]3(C)C(=C2C1)CC[C@@H]1[C@@]2(C)CC[C@H](O)C(C)(C)[C@@H]2CC[C@]13C	-5.62
Humulus lupulus	Cc1ccc2c(c1)C(C(C)C)=CC[C@H]2C	-5.62
Magnolia officinalis	COc1cc2c(cc1O)[C@H](Cc1ccc(O)cc1)NCC2	-5.62
Valeriana officinalis	C=C(C)[C@@H]1CC[C@@]2(C)CCCC(=C)[C@@H]2C1	-5.61
Piper methysticum	COc1ccccc1/C=C/C(=O)OC1CC2CCC1(C)C2(C)C	-5.61
Scutellaria baicalensis	COc1ccc(-c2oc3cc(O)cc(O)c3c(=O)c2O)c(OC)c1O	-5.61
Humulus lupulus	CC1=C[C@@H]2[C@@H](C(C)C)CC=C(C)[C@@H]2CC1	-5.61
Humulus lupulus	COc1cc(O)cc(OC)c1C(=O)/C=C/c1ccc(O)cc1	-5.61
Humulus lupulus	O=C(O)c1cc(O)c(O)c(O)c1	-5.61
Humulus lupulus	COc1cc2c(c(OC)c1OC)-c1ccc(OC)c(=O)cc1[C@@H](NC(C)=O)CC2	-5.61
Humulus lupulus	C/C(=CCNc1ncnc2c1ncn2C1OC(CO)C(O)C1O)CO	-5.61
Valeriana officinalis	O=C(O)c1cc(O)c(O)c(O)c1	-5.6
Humulus lupulus	C/C(=C/CNc1ncnc2c1ncn2C1OC(CO)C(O)C1O)CO	-5.6
Magnolia officinalis	C/C1=CCC/C(C)=C/CC(C)(C)/C=C/C1	-5.6
Magnolia officinalis	COc1c(O)ccc2c1-c1c3c(cc4c1[C@H](C2)NCC4)OCO3	-5.6
Valeriana officinalis	C[C@@H]1CCC2=C3[C@](C)(CC[C@]31O)O[C@@](C)(O)C2=O	-5.59
Valeriana officinalis	CC1CCCC2CCC(C(C)(C)O)CC12C	-5.59
Valeriana officinalis	CCCCCC(=O)OCC(C)=CC1CCC(C)C2CCC(C)=C12	-5.59
Piper methysticum	COc1cc(/C=C/c2ccc3c(c2)OCO3)oc(=O)c1	-5.58
Scutellaria baicalensis	COc1cccc(O)c1-c1cc(=O)c2c(O)c(OC)c(OC)c(OC)c2o1	-5.58
Valeriana officinalis	CC1(C)C2CC(OC3OC(CO)C(O)C(O)C3O)C1(C)CC2O	-5.57
Humulus lupulus	COc1cc(O)c(C(=O)/C=C/c2ccc(O)cc2)c(OC)c1	-5.57
Valeriana officinalis	C[C@@H]1CCC=C2CC[C@@H](C(C)(C)O)C[C@]21C	-5.56
Valeriana officinalis	C=C1CCC2(C(C)C)OC2C2C1CCC2(C)O	-5.56
Humulus lupulus	CCC(C)C(=O)c1c(O)c(CC=C(C)C)c(O)c(CC=C(C)C)c1O	-5.56
Humulus lupulus	C=C1CC[C@@]23[C@@H]([C@@H]12)[C@H](C(C)C)CC[C@H]3C	-5.56
Magnolia officinalis	C=C1CCC[C@]2(C)CC[C@H]3C(C)(C)C[C@]132	-5.55
Scutellaria baicalensis	CC[C@H](/C=C/[C@@H](C)[C@H]1CC[C@H]2[C@@H]3CC=C4C[C@@H](O)CC[C@]4(C)[C@H]3CC[C@]12C)C(C)C	-5.54
Melissa officinalis	O=C(O)C(O)Cc1ccc(O)c(O)c1	-5.54
Magnolia officinalis	CC(=O)N1CCc2cc3c(c4c2[C@H]1Cc1ccccc1-4)OCO3	-5.54
Valeriana officinalis	C[C@@H]1CC[C@H]2[C@@H]1[C@H]1[C@@H](CC[C@@]2(C)O)C1(C)C	-5.53
Valeriana officinalis	CC1=C[C@@H]2[C@@H](C(C)C)CC=C(CO)[C@@H]2CC1	-5.53
Humulus lupulus	C=C(C)C1CCC2(C)CCCC3(CO3)C2C1	-5.53
Magnolia officinalis	C=CCc1ccc(Oc2cc(C=CCOC)cc(O)c2O)cc1	-5.53
Valeriana officinalis	C=C(C)[C@@H]1CCC2=CC(=O)C[C@@H](C)[C@]2(C)C1	-5.52
Valeriana officinalis	CC(=O)[C@@H]1[C@H](C(C)C)CC[C@@]2(C)CCC[C@@H]12	-5.52
Valeriana officinalis	C=C(C)[C@@H]1[C@H]2[C@@H](C)CC[C@@H]3[C@@H](C)CCC231	-5.52
Humulus lupulus	C/C1=CCC[C@H]2O[C@]2(C)C/C=C/C(C)(C)C1	-5.52
Valeriana officinalis	CC1=CCC[C@H](C)[C@@]12CC[C@@H](C(C)(C)O)C2	-5.51
Valeriana officinalis	CC(C)=CCCC(C)c1ccc(C)cc1	-5.51
Humulus lupulus	COc1c(CC=C(C)C)c(O)c(CC=C(C)C)c(O)c1C(=O)/C=C/c1ccc(O)cc1	-5.51
Magnolia officinalis	CC1=CCC[C@H](C)[C@@]12CC[C@@H](C(C)(C)O)C2	-5.51
Piper methysticum	COc1cc(/C=C/c2ccccc2)oc(=O)c1	-5.5
Piper methysticum	COc1cc(/C=C/c2ccccc2)oc(=O)c1	-5.5
Magnolia officinalis	C=C(CCC=C(C)C)[C@@H]1CC=C(C)CC1	-5.5
Valeriana officinalis	Cc1ccc(C(C)C)c(O)c1	-5.49
Valeriana officinalis	O=C(O)/C=C/c1ccc(O)c(O)c1	-5.49
Valeriana officinalis	CC1=C2[C@H](/C=C(C)CO)CC[C@@H](C)[C@H]2CC1	-5.48
Melissa officinalis	O=C(O)[C@H](O)Cc1ccc(O)c(O)c1	-5.48
Humulus lupulus	CC(C)=CCc1c(O)c(CC=C(C)C)c(O)c(C(=O)C=Cc2ccc(O)cc2)c1O	-5.48
Humulus lupulus	Nc1ncnc2c1ncn2[C@@H]1O[C@H](COP(=O)(O)OP(=O)(O)O)C(O)[C@@H]1O	-5.48
Humulus lupulus	CC(C)=CCc1c(O)c(CC=C(C)C)c(O)c(C(=O)C(C)C)c1O	-5.47
Valeriana officinalis	CC(C)=CCC/C(C)=C/CC/C(C)=C/COP(=O)(O)OP(=O)(O)O	-5.46
Valeriana officinalis	COc1cc(/C=C/C(=O)O)ccc1O	-5.46
Valeriana officinalis	CC(=O)O[C@H]1C[C@]2(O)C(COC(=O)C(OC(=O)CC(C)C)C(C)C)=CO[C@@H](OC(=O)CC(C)C)[C@@H]2[C@@]1(O)COC(=O)CC(C)C	-5.46
Valeriana officinalis	C/C(C=O)=C[C@@H]1CC[C@@H](C)[C@H]2CC[C@]3(C)O[C@]123	-5.46
Piper methysticum	COC1=CC(=O)O[C@@H](CCc2ccc(OC)c(OC)c2)C1	-5.46
Humulus lupulus	CC(C)C(=O)c1c(O)cc(O)cc1OC1OC(CO)C(O)C(O)C1O	-5.46
Humulus lupulus	COc1cc2c(c(O)c1C(=O)/C=C/c1ccc(O)cc1)C=CC(C)(C)O2	-5.46
Humulus lupulus	CCCC(CCO)SCC(NC(=O)CCC(N)C(=O)O)C(=O)NCC(=O)O	-5.45
Humulus lupulus	COc1cc2c(c(O)c1C(=O)/C=C/c1ccc(O)cc1)CCC(C)(C)O2	-5.45
Valeriana officinalis	CC[C@H](CC[C@@H](C)[C@H]1CC[C@H]2[C@@H]3CC=C4C[C@@H](O)CC[C@]4(C)[C@H]3CC[C@]12C)C(C)C	-5.44
Humulus lupulus	C=C(C)[C@@H]1CC[C@H](C)C2=C(C1)[C@@H](C)CC2	-5.44
Humulus lupulus	CC(C)CCC[C@@H](C)[C@H]1CC[C@H]2[C@@H]3CC=C4C[C@@H](O)CC[C@]4(C)[C@H]3CC[C@]12C	-5.44
Humulus lupulus	COc1cc(/C=C/C(=O)O)ccc1O	-5.44
Magnolia officinalis	C=C(C)[C@@H]1CC[C@H](C)C2=C(C1)[C@@H](C)CC2	-5.44
Valeriana officinalis	C[C@@H]1CC[C@@H]2[C@@H]1[C@H]1[C@@H](CC[C@@]2(C)O)C1(C)C	-5.43
Valeriana officinalis	CC(=O)OCC(C)=CC1CCC(C)C2CCC(C)=C12	-5.43
Valeriana officinalis	CC1=C[C@@H]2[C@@H](CC1)[C@@](C)(O)CC[C@H]2C(C)C	-5.42
Piper methysticum	COC1=CC(=O)O[C@@H](CCc2ccc(OC)cc2)C1	-5.42
Humulus lupulus	C[C@@]1(CO)OP(=O)(O)OP(=O)(O)OC[C@H]1O	-5.42
Humulus lupulus	O=C(O)/C=C/c1ccc(O)c(O)c1	-5.42

Magnolia officinalis	COc1cc(/C=C/C=O)cc(OC)c1O	-5.42
Magnolia officinalis	Cc1c(C(=O)NN2CCCCC2)nn(-c2ccc(Cl)cc2Cl)c1-c1ccc(Cl)cc1	-5.42
Melissa officinalis	O=C(O)/C=C/c1ccc(O)c(O)c1	-5.41
Humulus lupulus	CC(C)(CCO)SCC(NC(=O)CCC(N)C(=O)O)C(=O)NCC(=O)O	-5.41
Humulus lupulus	CC1(C)C[C@@H]2[C@@H]1CC[C@]1(C)CCC[C@@]2(O)C1	-5.41
Humulus lupulus	COc1cc(C(=O)O)cc(OC)c1O	-5.41
Valeriana officinalis	C=C1C2OC(O)C3C1(O)CC(OC(C)=O)C3(CCl)O2	-5.4
Piper methysticum	COc1ccc2c(c1OC)Oc1c(OC)ccc3c1C(=C2)N(C)C(=O)C3=O	-5.4
Humulus lupulus	CC=C(CCC(C)C1CCC2C3=CCC4C(C)C(O)CCC4(C)C3CCC21C)C(C)C	-5.4
Humulus lupulus	C=C1CCC2C3C(C(C)C)CC[C@@]2(C)[C@H]13	-5.4
Piper methysticum	COC1=CC(=O)O[C@@H](/C=C/c2ccc3c(c2)OCO3)C1	-5.39
Scutellaria baicalensis	COc1cc([C@H]2OC[C@H]3[C@@H]2CO[C@@H]3c2cc(OC)c(O)c(OC)c2)cc(OC)c1O	-5.39
Humulus lupulus	CC(C)CC(=O)SCCNC(=O)CCNC(=O)C(O)C(C)(C)CO[P@SP2](=O)([O-])O[P@SP3](=O)([O-])OC[C@@H]1O[C@H](n2cnc3c(N)ncnc32)[C@@H](O)[C@H]1O[P@SP3](=O)([O-])[O-]	-5.39
Humulus lupulus	OCC1O[C@H](O)C(O)[C@@H](O)[C@@H]1O	-5.39
Humulus lupulus	C/C(=CCNc1ncnc2c1ncn2[C@@H]1O[C@H](COP(=O)(O)O)[C@@H](O)[C@H]1O)CO	-5.37
Scutellaria baicalensis	COc1cc(O)c2c(=O)cc(-c3c(O)cccc3O)oc2c1OC	-5.36
Humulus lupulus	CC(C)=CCCC(C)c1ccc(C)cc1	-5.36
Magnolia officinalis	COc1cc([C@H]2OC[C@H]3[C@@H]2CO[C@@H]3c2cc(OC)c(O)c(OC)c2)cc(OC)c1O	-5.36
Valeriana officinalis	CC(=O)O[C@H]1C[C@]2(O)C(COC(=O)C(OC(=O)CC(C)C)C(C)C)=CO[C@@H](OC(=O)CC(C)C)[C@@H]2[C@]1(O)CCl	-5.35
Piper methysticum	CC(=O)/C=C/C=C/c1ccccc1	-5.35
Melissa officinalis	O=C(O)C(=O)Cc1ccc(O)cc1	-5.35
Piper methysticum	COc1ccc(/C=C/c2cc(OC)cc(=O)o2)cc1	-5.34
Valeriana officinalis	CC1=C[C@H]2O[C@@H]3C[C@@H](O)C(C)(C34CO4)[C@@]2(CO)CC1	-5.33
Valeriana officinalis	CC(=O)O[C@@H]1C[C@@H](C)[C@H]2C[C@H]3CC[C@](C)(OC3(C)C)[C@@H]21	-5.33
Valeriana officinalis	CC(=O)OCC(C)=CC1CCC(C)C2CCC(C)=C12	-5.33
Magnolia officinalis	C=C(C)C1CCC(=C)C2CCC(C)C2C1	-5.33
Valeriana officinalis	CC(=O)OC/C(C)=CCC[C@]1(C)[C@H]2CC=C(C)[C@@H]1C2	-5.31
Valeriana officinalis	CC1CCC(C2(C1CC(CC2)C(C)C)C)O	-5.31
Piper methysticum	COc1ccc(/C=C/c2cc(OC)cc(=O)o2)c(OC)c1	-5.31
Melissa officinalis	O=C(O)/C=C/c1ccccc1	-5.31
Melissa officinalis	N[C@@H](Cc1ccc(O)cc1)C(=O)O	-5.3
Humulus lupulus	CCC(C)C(=O)C1=C(O)C(O)(C(=O)CC=C(C)C)C(O)(CC=C(C)C)C1=O	-5.3
Humulus lupulus	CC(C)=CCc1c(O)c(CC=C(C)C)c(O)c(C(=O)CC(C)C)c1O	-5.29
Humulus lupulus	c1ccc(CNc2ncnc3nc[nH]c23)cc1	-5.29
Magnolia officinalis	O=C(O)C(=O)Cc1ccc(O)cc1	-5.29
Valeriana officinalis	CC1=CCC2C3C(C(C)C)CC[C@]2(C)C13	-5.28
Scutellaria baicalensis	COc1cc(O)cc2c1C(=O)CC(c1c(O)cccc1O)O2	-5.28
Humulus lupulus	CC1=CCC2C3C(C(C)C)CC[C@]2(C)C13	-5.28
Valeriana officinalis	COc1cc(/C=C/CO)ccc1O[C@@H]1OC(CO)[C@@H](O)[C@H](O)C1O	-5.27
Humulus lupulus	O=C(O)/C=C/c1ccccc1	-5.27
Valeriana officinalis	C=C(C)[C@@H]1CCC2=CCC[C@@H](C)[C@]2(C)C1	-5.26
Piper methysticum	COC1=CC(=O)O[C@@H](CCc2ccccc2)C1	-5.26
Humulus lupulus	C=C(C)[C@@H]1CCC2=CCC[C@@H](C)[C@]2(C)C1	-5.26
Humulus lupulus	COc1cc(C(=O)O)ccc1O	-5.26
Magnolia officinalis	C=CC(C)(O)CC/C=C(C)CCC=C(C)C	-5.26
Magnolia officinalis	COc1cc(C=CC(=O)NCCCCN)ccc1O	-5.26
Humulus lupulus	O=C(O)Cc1c[nH]c2ccccc12	-5.25
Humulus lupulus	O=C(O)/C=C/c1ccc(O)cc1	-5.24
Humulus lupulus	C/C(=CCNc1ncnc2nc[nH]c12)CO	-5.24
Magnolia officinalis	COc1cc(C=CC(=O)NCCCCN)ccc1O	-5.24
Valeriana officinalis	CC(=O)OCC1=CO[C@@H](OC(=O)CC(C)C)[C@H]2C1=C[C@H](OC(=O)CC(C)C)[C@]21CO1	-5.23
Valeriana officinalis	CC1=C2[C@H](/C=C(C)C=O)CC[C@@H](C)[C@H]2CC1	-5.23
Valeriana officinalis	CC(C)=CC1C2=CC[C@H](C)[C@H]2CC[C@@H]1C	-5.23
Humulus lupulus	CC(C)=CCNc1ncnc2nc[nH]c12	-5.23
Magnolia officinalis	COc1cc(/C=C/C=O)ccc1O	-5.23
Magnolia officinalis	C=CCc1ccc(Oc2cc(CC=C)cc(O)c2O)cc1	-5.23
Valeriana officinalis	C=C(C)C1CCC2(C)C(O)CCC(C)C2C1	-5.22
Valeriana officinalis	CC1=CCC(C(C)(C)O)CC1	-5.21
Valeriana officinalis	CC(=O)OCC1=CO[C@@H](OC(=O)CC(C)C)[C@H]2[C@@H]1CC[C@@H]2C	-5.21
Humulus lupulus	CC(C)=CC1C(=O)C2(O)C(O)=C(C(=O)CC(C)C)C(=O)C2(O)CC1C(C)(C)O	-5.21
Humulus lupulus	C[C@H]1[C@H](C)CC[C@]2(C)CC[C@]3(C)C(C=C[C@@H]4[C@@]5(C)CC[C@H](O)C(C)(C)[C@@H]5CC[C@]43C)[C@H]12	-5.21
Humulus lupulus	C=C1C[C@]23C[C@@]1(O)CC[C@H]2[C@]1(C=O)CCC[C@@](C)(C(=O)O)[C@H]1[C@@H]3C(=O)O	-5.21
Magnolia officinalis	CC1=CCC(C(C)(C)O)CC1	-5.21
Magnolia officinalis	COC1=CC(=O)C=C(OC)C1(C/C=C/c1ccc(O)c(OC)c1)OC	-5.21
Magnolia officinalis	CC1=CCC2C(CC2(C)C)C(C)CCC1O	-5.21
Valeriana officinalis	C[C@H]1CC[C@@H](C(C)(C)O)CC2=C1CC[C@@H]2C	-5.2
Humulus lupulus	CC1=C[C@H]2[C@H](C(C)C)CC[C@@H](C)[C@]2(O)CC1	-5.2
Humulus lupulus	C[C@H]1CC[C@@H](C(C)(C)O)CC2=C1CC[C@@H]2C	-5.2
Scutellaria baicalensis	COc1c(OC)c(O)c2c(=O)cc(-c3ccccc3O)oc2c1OC	-5.19
Humulus lupulus	CCC(C)C(=O)C1=C(O)C(O)(C(=O)C=CC(C)(C)O)C(O)(CC=C(C)C)C1=O	-5.19
Magnolia officinalis	Cc1ccc(C(C)C)cc2c(C)ccc1-2	-5.19
Magnolia officinalis	COc1cc(O)c2c(c1)CC1c3c(c(O)c(OC)c(O)c3-2)CC[N+]1(C)C	-5.19
Valeriana officinalis	CC/C=CC[C@H]1C(=O)CC[C@@H]1CC(=O)O	-5.18
Valeriana officinalis	C=C[C@@](C)(O)CC/C=C(C)CCC=C(C)C	-5.18
Valeriana officinalis	CC1=C2[C@H](/C=C(/C)C(=O)O)CC[C@@H](C)[C@H]2CC1	-5.17
Valeriana officinalis	CC(=O)C[C@@H]1CC[C@@H](C)[C@H]2CC[C@]3(C)O[C@]123	-5.17
Valeriana officinalis	CC(C)CC(=O)O[C@H]1C[C@@H]2CC[C@@]1(C)C2(C)C	-5.17
Humulus lupulus	C=C(C)[C@@H]1CCC2=CCC[C@H](C)[C@@]2(C)C1	-5.17
Valeriana officinalis	O=C(O)/C=C/c1ccc(O)cc1	-5.16
Valeriana officinalis	CC(C)=C[C@]1(O)[C@@H]2CC[C@H](C)[C@H]2CC[C@@H]1C	-5.16
Valeriana officinalis	O=C(O)/C=Cc1ccc(O)cc1	-5.16
Melissa officinalis	O=C(O)/C=C/c1ccc(O)cc1	-5.16
Valeriana officinalis	CC(C)C1=CCC(C)(O)CC1	-5.15
Humulus lupulus	COc1cc(O)c(CC(O)C(C)(C)OC)c(O)c1C(=O)C=Cc1ccc(O)cc1	-5.15
Valeriana officinalis	CC(=O)OCC1=CO[C@@H](OC(=O)CC(C)C)[C@H]2C1=C[C@H](OC(=O)CC(C)C)[C@]2(O)CCl	-5.13
Valeriana officinalis	C=C(CC/C=C(/C)CO)[C@@H]1CC=C(C)CC1	-5.12
Valeriana officinalis	CC(C)CC(=O)OCC1=CO[C@@H](OC(=O)CC(C)C)[C@H]2C1=C[C@@H](O)[C@@]21CO1	-5.11
Scutellaria baicalensis	COc1cc(OC)c2c(c1)OC(c1c(OC)cccc1OC)C(OC)C2=O	-5.11
Magnolia officinalis	NCCc1ccc(O)c(O)c1	-5.11
Magnolia officinalis	CC1CC(O)C2C(C1)C2(C)C	-5.1

Humulus lupulus	CC1CCC2C1C1C(CCC23CO3)C1(C)C	-5.09
Magnolia officinalis	C=CCc1ccc(O)c(-c2cc(CC=C)ccc2O)c1	-5.09
Scutellaria baicalensis	O=C(O)/C=C/c1ccc(O)cc1	-5.08
Humulus lupulus	COc1cc(O)c2c(c1C(=O)C=Cc1ccc(O)cc1)OC(C)(C)C(O)C2	-5.07
Valeriana officinalis	CC(=O)OC1CC2(C)OC(C)(C)C1CC1C(C)CCC12	-5.06
Valeriana officinalis	CC(C)=CC1=C(C)CCC2C(C)CCC12	-5.06
Magnolia officinalis	CC1(C)C(=O)[C@]2(C)CC[C@H]1C2	-5.06
Magnolia officinalis	COc1cc(/C=C/CO)ccc1O	-5.05
Valeriana officinalis	C/C1=CCC/C(C(=O)O)=C/[C@@H]2[C@H](CC1)C2(C)C	-5.04
Scutellaria baicalensis	O=c1cc(-c2ccccc2)oc2ccc(O)cc12	-5.04
Valeriana officinalis	CC(=O)c1cc2ccncc2cn1	-5.03
Humulus lupulus	OCC1OC(O)C(O)C(O)C1O	-5.03
Humulus lupulus	C=C(CCC=C(C)C)C1CCC=C(CCC=C(C)C)C1	-5.03
Humulus lupulus	CC1=CCC[C@@H]2CCC(=C(C)C)C[C@@]12C	-5.03
Magnolia officinalis	C=CCc1ccc(O)c(-c2ccc(CC=C)c(OC)c2)c1	-5.03
Humulus lupulus	C=C(CCC=C(C)C)C1CC=C(CCC=C(C)C)CC1	-5.02
Magnolia officinalis	CCCCCCCCCCCCCCCC(=O)OCC(OC(=O)CCCCCCCCCCCCCCC)C1OC(=O)C(O)=C1O	-5.02
Valeriana officinalis	CC(C)=CC1C2=CCC(C)C2CCC1C	-5.01
Humulus lupulus	CC1=C2C=C(C(C)C)CCC2(C)CCC1	-5.01
Magnolia officinalis	COc1cc(C=O)cc(OC)c1O	-5.01
Valeriana officinalis	COc1ccc(/C=C/C(=O)O)cc1O	-5
Humulus lupulus	CC(C)=CCC/C(C)=C/CC/C(C)=C/CO	-5
Magnolia officinalis	C=CCc1ccc(O)c(OC)c1	-5
Valeriana officinalis	C=CCc1ccc(O)c(OC)c1	-4.99
Valeriana officinalis	CC(=O)OCC1=COC(OC(=O)C(OC(C)=O)C(C)C)C2C1=CC(OC(=O)CC(C)C)C21CO1	-4.99
Valeriana officinalis	CC(C)=CC1=C2CCC(C)C2CCC1C	-4.99
Magnolia officinalis	CCCc1ccc(O)c(-c2cc(CCC)ccc2O)c1	-4.99
Humulus lupulus	c1coc(CNc2ncnc3[nH]cnc23)c1	-4.98
Magnolia officinalis	C=CCc1ccc(O)c(-c2cc(C=CC=O)ccc2O)c1	-4.98
Humulus lupulus	C[C@@H]1CC[C@@H]2[C@@H]1[C@H]1[C@@H](CC[C@@]23CO3)C1(C)C	-4.97
Humulus lupulus	CC12C=CCC(O)(C1)C1CC(C)(C)C1CC2	-4.97
Humulus lupulus	C[C@](O)(CO)[C@H](O)COP(=O)(O)O	-4.97
Humulus lupulus	CC(C)=CCC/C(C)=C/COP(=O)(O)OP(=O)(O)O	-4.97
Magnolia officinalis	CC1(C)C2CCC(C2)C1(C)O	-4.97
Valeriana officinalis	CC(=O)O[C@H]1C=C2C(COC(=O)CC(C)C)=CO[C@@H](OC(=O)CC(C)C)[C@@H]2[C@@]12CO2	-4.96
Valeriana officinalis	COc1cc(C(C)C)cc(OC)c1C	-4.96
Valeriana officinalis	CCC(C)C(=O)OC1OC=C(COC(C)=O)C2=CC(OC(C)=O)C3(CO3)C21	-4.96
Humulus lupulus	CC1=Cc2c(C(C)C)ccc(C)c2CC1	-4.96
Magnolia officinalis	C=C1[C@H](O)C[C@@H]2C[C@H]1C2(C)C	-4.96
Valeriana officinalis	CC(C)=C[C@H]1[C@@H](C)CC[C@@H]2[C@@H](C)CC[C@]21O	-4.93
Valeriana officinalis	C=C1[C@@H]2OC[C@]3(O)[C@@H](OC(C)=O)C[C@@]1(O)[C@@H]3[C@H](OC(=O)CC(C)C)O2	-4.93
Valeriana officinalis	C=C1C(OC(C)=O)CC2(C(C)C)CC12	-4.93
Humulus lupulus	CC1=CC[C@@]23C[C@@H]1[C@@](C)(CO)[C@@H]2CC[C@H]3C	-4.93
Humulus lupulus	C=C1C/C=C/C(C)(C)C/C=C(C)CCC1	-4.93
Magnolia officinalis	C=Cc1ccc(O)c(OC)c1	-4.93
Humulus lupulus	C=CC1(C)CCC(=C(C)C)CC1C(=C)C	-4.92
Passiflora incarnata	COc1ccc2c3c([nH]c2c1)C(C)=NCC3	-4.91
Humulus lupulus	O=C(O)c1ccc(O)cc1	-4.91
Valeriana officinalis	CC(=O)OC[C@@]1(O)[C@@H](OC(C)=O)C[C@]2(O)C(COC(=O)C(OC(=O)CC(C)C)C(C)C)=CO[C@@H](OC(=O)CC(C)C)[C@H]12	-4.9
Valeriana officinalis	C=C1COC(=O)C2C1CC(O)C2CO	-4.9
Magnolia officinalis	C=C[C@](C)(O)CCC=C(C)C	-4.9
Humulus lupulus	CC(C)C[C@H](N)C(=O)O	-4.89
Valeriana officinalis	C=C[C@](C)(O)CCC=C(C)C	-4.88
Valeriana officinalis	CC(C)=C[C@H]1[C@@H](C)CC[C@@H]2[C@@H](C)CC[C@]21O	-4.88
Valeriana officinalis	CC1(CO)C2CCC1(C)C(OC1OC(CO)C(O)C(O)C1O)C2	-4.88
Melissa officinalis	C=C[C@](C)(O)CCC=C(C)C	-4.88
Humulus lupulus	C=C[C@](C)(O)CCC=C(C)C	-4.88
Valeriana officinalis	CC(C=O)=CCC1=C(C)CCCC1(C)C	-4.86
Valeriana officinalis	CC1(C)[C@@H]2CC[C@@]1(C)C(=O)C2	-4.85
Humulus lupulus	CC/C=CC/C=CC/C=CCCCCCCCC(=O)O	-4.85
Magnolia officinalis	CC1(C)[C@@H]2CC[C@@]1(C)C(=O)C2	-4.85
Valeriana officinalis	CC/C=CC[C@H]1C(=O)CC[C@@H]1CC(=O)OC	-4.84
Valeriana officinalis	CC(C)[C@@H]1CC[C@]2(C)CCCC(=O)[C@]2(C)C1	-4.84
Valeriana officinalis	CC(=O)OCC1=COC(OC(=O)CC(C)(C)OC(C)=O)C2C1=CC(OC(=O)CC(C)C)C21CO1	-4.84
Melissa officinalis	O=C(O)/C=C/c1ccccc1	-4.84
Humulus lupulus	COc1cc(C=O)ccc1O	-4.84
Valeriana officinalis	CC(=O)O[C@@H]1CC(C)=C2[C@H]1[C@H](C)CC[C@H]2/C=C(C)C(=O)O	-4.83
Magnolia officinalis	C[C@H]1C(=O)C[C@H]2C[C@@H]1C2(C)C	-4.83
Scutellaria baicalensis	O=C(O)/C=C/c1ccccc1	-4.82
Humulus lupulus	CC(C)=CCCC1(C)C2CC=C(C)C1C2	-4.82
Magnolia officinalis	COc1cc2c(c(O)c1OC)CC1c3c(c(OC)c4c(c3-2)OCO4)CC[N+]1(C)C	-4.82
Valeriana officinalis	CC(C)=C[C@H]1C2=C(CC[C@H]1C)[C@H](C)CC2	-4.81
Valeriana officinalis	CC(C)C12CC=C(C=O)C1C2	-4.81
Magnolia officinalis	CCCCC/C=CC/C=C/CCCCCCCC(=O)O	-4.81
Magnolia officinalis	CN1CCc2cc3c(c4c2[C@H]1Cc1ccccc1-4)OCO3	-4.81
Magnolia officinalis	CC(C)COC(=O)c1ccccc1C(=O)OCC(C)C	-4.81
Valeriana officinalis	COc1ccc(C=O)cc1O	-4.8
Magnolia officinalis	C=C1[C@H](O)C[C@@]2(C(C)C)C[C@@H]12	-4.8
Magnolia officinalis	C=C(C)[C@@H]1CC[C@@H](C)C(=O)C1	-4.8
Valeriana officinalis	CC(=O)OC(C)(C)C1CC=C(C)CC1	-4.79
Valeriana officinalis	CC(=O)OCC1(C)OC2(C)CCC1CC1C(C)CCC12	-4.79
Piper methysticum	COC1=CC(=O)O[C@@H](/C=C/c2ccccc2)C1	-4.79
Humulus lupulus	C=CC(C)(CCC=C(C)C)OC(=O)CC(C)C	-4.79
Valeriana officinalis	CC(C)=C1C/C=C(C)CC/C=C(C)CC1	-4.78
Humulus lupulus	CC(C)C(CCO)SCC(N)C(=O)O	-4.78
Valeriana officinalis	CCCCCCCC/C=CCCCCCCCC(=O)O	-4.77
Valeriana officinalis	CC1=CCC(O)(C(C)C)CC1	-4.77
Melissa officinalis	N[C@@H](Cc1ccccc1)C(=O)O	-4.76
Valeriana officinalis	C=C[C@]1(C)CC[C@@H](C(C)(C)O)C[C@H]1C(=C)C	-4.74
Valeriana officinalis	C=C1CCC2=C1CCC(C)C2C(C)CC	-4.74
Valeriana officinalis	C=C1[C@H](O)C[C@]2(C(C)C)C[C@H]12	-4.73

Valeriana officinalis	C/C1=C[C@H]2[C@@H](CC/C(C)=C/CC1)C2(C)C	-4.73
Melissa officinalis	CC(C)=C1CC[C@@H](C)CC1=O	-4.73
Humulus lupulus	Cc1cc(C=O)cc(C)c1C	-4.71
Humulus lupulus	C=C(C)[C@@H]1CCC(C)=C2CC[C@H](C)[C@@H]2C1	-4.71
Magnolia officinalis	CC1=CC(O)C2CC1C2(C)C	-4.71
Valeriana officinalis	CC(=O)OCC1=CO[C@@H](OC(=O)CC(C)C)[C@H]2C1=C[C@H](OC(=O)CC(C)(C)OC(C)=O)[C@]21CO1	-4.7
Valeriana officinalis	C[C@@H]1CC[C@@H]2[C@@H]1C[C@H]1CC[C@]2(C)OC1(C)C	-4.7
Humulus lupulus	O=c1c(O)c(-c2ccccc2)oc2ccccc12	-4.7
Valeriana officinalis	CC(=O)OCC1=CO[C@@H](OC(=O)C=C(C)C)[C@H]2C1=C[C@H](OC(=O)CC(C)C)[C@]21CO1	-4.69
Magnolia officinalis	CC1(C)C2CC3C(C2)C31C	-4.69
Valeriana officinalis	CC1(C)C2CC3C(C2)C31C	-4.68
Piper methysticum	COc1ccc(/C=C/C(=O)O)cc1OC	-4.68
Piper methysticum	CC(=O)/C=C/C=C/c1ccc2c(c1)OCO2	-4.67
Humulus lupulus	C=CC(=C)CC/C=C(/C)CCC=C(C)C	-4.67
Humulus lupulus	CC(=O)[C@@H](O)[C@H](O)COP(=O)(O)O	-4.67
Magnolia officinalis	COc1c(O)cc2c3c1-c1ccccc1C[C@H]3NCC2	-4.67
Magnolia officinalis	CC1=CC(=O)[C@H](C(C)C)CC1	-4.66
Valeriana officinalis	C=C(CCC=C(C)C)[C@@H]1CC=C(C)CC1	-4.65
Humulus lupulus	C=C/C(C)=C/C/C=C(C)CCC=C(C)C	-4.65
Magnolia officinalis	CC(=O)OC/C=C(/C)CCC=C(C)C	-4.65
Valeriana officinalis	CC(=O)OC1CC2CCC1(C)C2(C)C	-4.64
Valeriana officinalis	CCCCC(=O)O	-4.64
Valeriana officinalis	C=C(C)[C@H]1C2[C@H](C)CC[C@H]3[C@H](C)CCC213	-4.64
Valeriana officinalis	CC(=O)OC1C=C(C)C2CC1C2(C)C	-4.64
Humulus lupulus	CC1CCC2C1C1C(CCC2(C)O)C1(C)C	-4.64
Valeriana officinalis	CC(C)=C1CC[C@H](C)C2=C(C1)[C@@H](C)CC2	-4.63
Humulus lupulus	C=C1CC[C@@H]2[C@H]([C@@H]3[C@H](C)CC[C@H]13)C2(C)C	-4.63
Magnolia officinalis	C=C[C@@](C)(CCC=C(C)C)OC(C)=O	-4.63
Valeriana officinalis	C=C(C)C1CC=C(C)CC1	-4.6
Valeriana officinalis	C=C(C)[C@@H]1CC=C(C=O)CC1	-4.6
Humulus lupulus	C=C(C)C1CC=C(C)CC1	-4.6
Magnolia officinalis	C=C(C)C1CC=C(C)CC1	-4.6
Magnolia officinalis	C=C(C)[C@H]1C=C2[C@H](C)CC[C@@H]2[C@H](C)CC1	-4.6
Humulus lupulus	CC1=CCC(C(C)C)CC2C(C)=CCC12	-4.59
Humulus lupulus	CC(C)=CCCC(C)=CCOC(=O)C(C)C	-4.59
Valeriana officinalis	CC1(C)C[C@@H]2[C@H](C1)[C@@]1(C)CCC[C@]2(C)C1O	-4.58
Valeriana officinalis	C=C(C)[C@H]1C/C=C(C)CC/C=C(C)CC1	-4.58
Humulus lupulus	C/C1=CCC/C(C)=C/CC(C)(C)/C=C/C1	-4.58
Humulus lupulus	CCCCC/C=CC/C=C/CCCCCCCC(=O)O	-4.58
Melissa officinalis	C=C(C)[C@H](CO)CC=C(C)C	-4.57
Humulus lupulus	C=CC(=C)CC/C=C(C)CCC=C(C)C	-4.57
Passiflora incarnata	COc1ccc2c(c1)[nH]c1c(C)nccc12	-4.56
Melissa officinalis	CC(C)=CCC/C(C)=CCO	-4.56
Humulus lupulus	C/C1=C[C@H]2[C@@H](CC/C(C)=C/CC1)C2(C)C	-4.56
Valeriana officinalis	CC(=O)O[C@H]1C[C@]2(O)C(COC(=O)C(OC(=O)CC(C)C)C(C)C)=COC(OC(=O)CC(C)C)[C@@H]2[C@@]12CO2	-4.55
Melissa officinalis	C=C1CC[C@@H]2[C@H]([C@H]3[C@H]1CC[C@]3(C)O)C2(C)C	-4.55
Magnolia officinalis	CC1=C2C(CC1)C(C)CCC1C2C1(C)C	-4.55
Magnolia officinalis	Cc1cccc(C(C)C)c1	-4.54
Magnolia officinalis	CCCCC=CC(C)OC(=O)C1CCC1	-4.54
Valeriana officinalis	Cc1ccc(C(C)C)cc1	-4.53
Valeriana officinalis	CC(C)=CC1CC[C@@H](C)[C@H]2CCC(C)=C12	-4.53
Magnolia officinalis	CC1=CCC2C3C(C(C)C)CC[C@@]2(C)C13	-4.53
Valeriana officinalis	CC(=O)O[C@@H]1C[C@@H]2CC[C@@]1(C)C2(C)C	-4.52
Scutellaria baicalensis	COc1ccc2[nH]cc(CCN)c2c1	-4.52
Melissa officinalis	CC(C)=CCCC(C)CCO	-4.52
Valeriana officinalis	C=C[C@]1(C)CCC(=C(C)C)C=C1C(C)C	-4.51
Valeriana officinalis	C=C(C)[C@@H]1CC[C@H](C)C2=C(C1)[C@@H](C)CC2	-4.51
Passiflora incarnata	c1ccc2c(c1)[nH]c1cnccc12	-4.51
Piper methysticum	O=C(CCc1ccccc1)N1C=CC(O)CC1	-4.51
Humulus lupulus	CCCC(CCO)SCC(N)C(=O)O	-4.51
Magnolia officinalis	COc1cc2c3c(c1OC)-c1ccccc1C[C@H]3NCC2	-4.51
Magnolia officinalis	O=CCc1ccc(O)cc1	-4.51
Magnolia officinalis	CC(=O)O[C@@H]1C[C@@H]2CC[C@@]1(C)C2(C)C	-4.5
Magnolia officinalis	CC1=CC=CC(C)(C)C=C1	-4.5
Humulus lupulus	CC1=CCC(C)(C)C=CCC(C)(O)CCC1	-4.49
Magnolia officinalis	C=CCc1ccc(O)cc1	-4.49
Valeriana officinalis	C=C1C=CC(C(C)C)CC1	-4.48
Humulus lupulus	Cc1ccc2c(c1)[C@H](C(C)C)CC[C@@H]2C	-4.48
Magnolia officinalis	CC1=CC[C@@]23CC1C(C)(C)[C@@H]2CC[C@H]3C	-4.48
Valeriana officinalis	CC1=C[C@@H]2C(=C(C)CC[C@H]2C(C)C)CC1	-4.47
Passiflora incarnata	COc1ccc2c3c4n(c2c1)CC(C)(C)C4=NCC3	-4.47
Humulus lupulus	C=C1C=CC(C(C)C)CC1	-4.47
Humulus lupulus	O=C(O)CCC(=O)C(=O)O	-4.47
Valeriana officinalis	CCCCCC=CCC=CCC=CCC=CCCCC(=O)OCC	-4.46
Humulus lupulus	C=C1CC[C@@H](C(C)C)[C@@H]2C=C(C)CC[C@H]12	-4.46
Valeriana officinalis	C=C1CC[C@@H]2[C@H]([C@H]3[C@H]1CC[C@]3(C)O)C2(C)C	-4.45
Humulus lupulus	C[C@@H]1CC[C@H]2[C@@H]1[C@H]1[C@@H](CC[C@]2(C)O)C1(C)C	-4.45
Humulus lupulus	CC(C)=CCCc1ccoc1	-4.45
Magnolia officinalis	CC(C)=C1CC[C@H](C)C2=C(C1)[C@@H](C)CC2	-4.45
Magnolia officinalis	C=C1CC[C@@H]2[C@H]([C@@H]3[C@H](C)CC[C@H]13)C2(C)C	-4.45
Valeriana officinalis	CC1=C2[C@@H]([C@H](C)CC[C@H]2/C=C(C)C(=O)O)[C@H](O)C1	-4.44
Valeriana officinalis	CC1CC(O)CC2=CC(=O)C3C(C3(C)C)C21C	-4.44
Humulus lupulus	CC(C)=CCOP(=O)(O)OP(=O)(O)O	-4.44
Magnolia officinalis	CC1(C)C2CC=C(C=O)C1C2	-4.44
Magnolia officinalis	Cc1ccc(C(C)C)cc1	-4.43
Magnolia officinalis	CC1=C[C@@H]2C(=C(C)CC[C@H]2C(C)C)CC1	-4.43
Humulus lupulus	CC1=C[C@@H]2[C@H](CC1)C(C)=CC[C@H]2C(C)C	-4.42
Humulus lupulus	C[C@H]1CC[C@@]2(O)[C@@H]1[C@H]1[C@@H](CC[C@@H]2C)C1(C)C	-4.42
Humulus lupulus	C=C1CC[C@@H]2[C@H]([C@H]3[C@H]1CC[C@]3(C)O)C2(C)C	-4.42
Passiflora incarnata	CC1=NCCc2c1[nH]c1ccccc21	-4.41
Humulus lupulus	CC(C)=C1C/C=C(C)CC/C=C(C)CC1	-4.41

Magnolia officinalis	C=CCc1cc(-c2cc(OC)ccc2OC)ccc1OC	-4.41
Valeriana officinalis	CC(=O)OCC1=CC[C@@H]2C[C@H]1C2(C)C	-4.4
Piper methysticum	CC(=O)/C=C/CCc1ccccc1	-4.4
Humulus lupulus	C[C@@H]1CC[C@H]2[C@@H]1[C@H]1[C@@H](CC[C@@]23CO3)C1(C)C	-4.4
Humulus lupulus	CC1=CCC(C(C)C)=CC1	-4.4
Magnolia officinalis	C/C(=C/CCC1(C)C2CC3C(C2)C31C)CO	-4.4
Valeriana officinalis	C=C1CC[C@H]2O[C@]2(C)CC[C@@H]2[C@@H]1CC2(C)C	-4.39
Valeriana officinalis	CC(=O)/C=C/C1=C(C)CCCC1(C)C	-4.39
Humulus lupulus	CCCCCCCCCCCCCCCC(=O)O	-4.39
Valeriana officinalis	CC(C)C1CCC2C3(CCC(C)C2(C)C1)SCCS3	-4.38
Melissa officinalis	CC(C)=CCC/C(C)=CC=O	-4.38
Magnolia officinalis	CCCCCCCC/C=CCCCCCCCC(=O)O	-4.38
Valeriana officinalis	CC(C)=CCCC(C)CCO	-4.36
Valeriana officinalis	CCCCCCCCCCCCCCCCCCCCCC(=O)O	-4.36
Valeriana officinalis	C=C1CC=C(C(C)C)CC1	-4.36
Humulus lupulus	C[C@@H]1CC[C@@H]2[C@@H]1[C@H]1[C@@H](CC[C@@]2(C)O)C1(C)C	-4.36
Humulus lupulus	C/C(=CCOP(=O)(O)OP(=O)(O)O)CO	-4.36
Magnolia officinalis	CC12CCC(CC1)C(C)(C)O2	-4.36
Magnolia officinalis	O=Cc1ccc(O)cc1	-4.36
Melissa officinalis	CC1=CCC2C(C1)C2(C)C	-4.35
Humulus lupulus	CC1CCC2C1C1C(CCC23CO3)C1(C)C	-4.35
Humulus lupulus	O=Cc1ccccc1	-4.35
Humulus lupulus	C=C1CC/C=C(C)CC[C@@H]2[C@@H]1CC2(C)C	-4.35
Magnolia officinalis	CCCCCCCCCCCC(C)(CC)CO	-4.35
Valeriana officinalis	CC12CCC(CC1)C(C)(C)O2	-4.34
Scutellaria baicalensis	NCCc1c[nH]c2ccc(O)cc12	-4.34
Magnolia officinalis	CC1(C)C2=CC=CC(C)(C)C23CCC1C3	-4.33
Magnolia officinalis	CC1CCC2C1=CC(C1(C)CO1)CCC2C	-4.33
Valeriana officinalis	CCCCCCCCCCCCCCCC(=O)O	-4.32
Valeriana officinalis	C[C@@H]1CC[C@@H]2[C@@H]1[C@H]1[C@@H](CC[C@]2(C)O)C1(C)C	-4.32
Humulus lupulus	C=C(C)C1CCC2(C)CCCC(C)(O)C2C1	-4.32
Magnolia officinalis	C=C1CC[C@H]2O[C@]2(C)CC[C@@H]2[C@@H]1CC2(C)C	-4.32
Magnolia officinalis	CCCCCC(=O)c1ccco1	-4.32
Valeriana officinalis	C=C1C2CCC(C2)C1(C)C	-4.31
Valeriana officinalis	CC(C)CCOC(=O)CC(C)C	-4.31
Melissa officinalis	CC(C)=CCC[C@@H](C)CC=O	-4.31
Humulus lupulus	CCC(=O)OC/C=C(C)CCC=C(C)C	-4.31
Humulus lupulus	CCCCCCOC(=O)C(C)CC	-4.31
Valeriana officinalis	CC(C)CC(=O)OCC1=CCC2CC1C2(C)C	-4.3
Humulus lupulus	C=C1CC[C@@H]2[C@H]([C@@H]3[C@H](C)CC[C@@H]13)C2(C)C	-4.3
Humulus lupulus	C=C1/C=C/[C@H](C(C)C)CC/C(C)=C/CC1	-4.3
Valeriana officinalis	CC(C)=CCOP(=O)(O)OP(=O)(O)O	-4.29
Melissa officinalis	CC(C)=CCC/C(C)=C/CO	-4.29
Humulus lupulus	CCC(C)OC(=O)C(C)C	-4.29
Humulus lupulus	C=C1CC[C@H]2O[C@]2(C)CC[C@@H]2[C@@H]1CC2(C)C	-4.29
Magnolia officinalis	CC1=C[C@H]2[C@H](C(C)C)CC[C@@](C)(O)[C@H]2CC1	-4.29
Valeriana officinalis	CC(C)=C1CC[C@@]2(C)CCC[C@@](C)(O)[C@@H]2C1	-4.28
Melissa officinalis	CC(C)=CCCC(C)CC=O	-4.28
Magnolia officinalis	C[C@@H]1CC[C@@H]2[C@@H]1[C@H]1[C@@H](CC[C@@]2(C)O)C1(C)C	-4.28
Magnolia officinalis	CC1=CCC(O)(C(C)C)CC1	-4.28
Valeriana officinalis	C[C@H]1CCC2(O)C(C)(C)C3CC[C@@]2(C)C1C3	-4.27
Valeriana officinalis	C=C1/C=C/[C@H](C(C)C)CC/C(C)=C/CC1	-4.27
Valeriana officinalis	CC(C)=C1CCC2=CCCC(C)C2(C)C1	-4.27
Valeriana officinalis	C[C@@H]1CC[C@@H]2[C@@H]1[C@H]1[C@@H](CC[C@@]2(C)O)C1(C)C	-4.25
Valeriana officinalis	CC1=C2[C@H](CC1)[C@H](C)CC[C@@H]1[C@H]2C1(C)C	-4.25
Scutellaria baicalensis	COc1ccc2[nH]cc(CCNC(C)=O)c2c1	-4.25
Humulus lupulus	CCCCCCOC(=O)C(C)C	-4.25
Humulus lupulus	CC1=C2[C@H](CC1)[C@H](C)CC[C@@H]1[C@H]2C1(C)C	-4.25
Magnolia officinalis	CCC(C)CCC(C)CC(C)C(C)C	-4.25
Magnolia officinalis	NCCc1ccc(O)cc1	-4.24
Valeriana officinalis	C/C1=CC=C(C(C)C)CC/C(C)=C/CC1	-4.23
Valeriana officinalis	CC1(C)C2[C@H]1CC[C@]1(C)CCC[C@](C)(O)[C@@H]21	-4.23
Valeriana officinalis	CC12CCC3C(C4C1CCC4(C)O2)C3(C)C	-4.23
Humulus lupulus	COC(=O)/C=C(C)CCC=C(C)C	-4.23
Magnolia officinalis	C=C[C@]1(C)CC[C@@H](C(C)(C)O)C[C@H]1C(=C)C	-4.23
Magnolia officinalis	CC/C=CC/C=CC/C=CCCCCCC=O	-4.23
Magnolia officinalis	CCCC=C1CCCCC1	-4.23
Valeriana officinalis	CC1(C)C2CCC3(C2)C1C(O)CCC3(C)C	-4.22
Valeriana officinalis	C=C(C)C=C1C(C)CCC2C(C)CCC12	-4.22
Humulus lupulus	CCCCC(C)CCC(=O)OC	-4.22
Humulus lupulus	CC(=O)CCC=C(C)C	-4.22
Humulus lupulus	CC1=C2[C@H]3[C@@H](CC[C@]2(C)CCC1)C3(C)C	-4.21
Humulus lupulus	C=C1C/C=C/C(C)(C)C/C=C(/C)CCC1O	-4.21
Magnolia officinalis	C=C(C)[C@H]1CC=C(C)C(=O)C1	-4.21
Magnolia officinalis	C[C@@H]1CCCC2=CC[C@@H]3[C@@H](C3(C)C)[C@]21C	-4.21
Humulus lupulus	CCC(C)COC(=O)C(C)CC	-4.2
Valeriana officinalis	C=C1CCCC(C)(C)[C@@]12CC=C(C)CC2	-4.19
Valeriana officinalis	C=C1CC[C@@H]2[C@H]([C@@H]3[C@H](C)CC[C@H]13)C2(C)C	-4.19
Humulus lupulus	CC(=O)OC/C=C(/C)CCC=C(C)C	-4.19
Valeriana officinalis	CC(C)[C@H]1CC[C@@]2(C)CCCC(=O)[C@@]2(C)C1	-4.18
Valeriana officinalis	CCCCCC(=O)c1ccco1	-4.18
Valeriana officinalis	C=CC(=C)CCC=C(C)C	-4.17
Valeriana officinalis	COC(/C=C(C)CCC=C(C)C)OC	-4.17
Valeriana officinalis	C=CC1(C)CCC(COC(C)=O)C(C)(C)C1C(=C)C	-4.17
Scutellaria baicalensis	COc1cc(OC)c2c(=O)c(OC)c(-c3c(OC)cccc3OC)oc2c1	-4.17
Melissa officinalis	C=C1CC/C=C(C)CC[C@@H]2[C@@H]1CC2(C)C	-4.17
Melissa officinalis	C=CC(=C)CCC=C(C)C	-4.17
Humulus lupulus	CC1=CCC(C)(C)C=CCC(C)C(=O)CC1	-4.17
Humulus lupulus	CC1=C2CC[C@@H](C)C2[C@H]2[C@@H](CC1)C2(C)C	-4.17
Humulus lupulus	CC1=CCC2C3C(C(C)C)CC[C@@]2(C)C13	-4.17
Magnolia officinalis	C=C1CC/C=C(C)CC[C@@H]2[C@@H]1CC2(C)C	-4.17

Valeriana officinalis	C=C[C@]1(C)CCC(C(=C)C)C[C@H]1C(=C)C	-4.16
Humulus lupulus	CC(C)=CCC/C(C)=C/CO	-4.16
Valeriana officinalis	C[C@@H]1CC[C@@H]2[C@@H]1[C@H]1[C@@H](CC[C@@]23CO3)C1(C)C	-4.15
Valeriana officinalis	C=C1CC/C=C(/C)CC[C@@H]2[C@H]1CC2(C)C	-4.14
Valeriana officinalis	CCCCC(=O)OCC(C)C	-4.14
Humulus lupulus	CCC(=O)OCCC(C)C	-4.14
Valeriana officinalis	CCCCCCCC=CCCCCCCCCC(=O)OC	-4.13
Melissa officinalis	CCC(=O)C1CCCCN1	-4.13
Melissa officinalis	CCC(=O)C1=NCCCC1	-4.13
Humulus lupulus	CC(C)C(S)CCO	-4.13
Humulus lupulus	C=CC(=C)CCC=C(C)C	-4.13
Magnolia officinalis	C[C@H]1CC[C@@H](C(C)(C)O)CC2=C1CC[C@@H]2C	-4.13
Valeriana officinalis	COc1ccc(OC)c(C(C)(C)C)c1	-4.12
Melissa officinalis	CC(=O)OC/C=C(C)CCC=C(C)C	-4.12
Valeriana officinalis	CC1=CCC2C3C(C(C)C)CC[C@@]2(C)C13	-4.1
Magnolia officinalis	CCCCCCCC/C=CCCCCCCCC(=O)OC	-4.1
Valeriana officinalis	COCc1cncc2c1CC[C@@H]2C	-4.09
Valeriana officinalis	C=C[C@@]1(C)CCC(=C(C)C)C[C@@H]1C(=C)C	-4.09
Valeriana officinalis	C=C1CC[C@H]2[C@@H]1[C@H]1[C@@H](CC[C@H]2C)C1(C)C	-4.09
Melissa officinalis	C=C1/C=C/[C@H](C(C)C)CC/C(C)=C/CC1	-4.09
Humulus lupulus	CC1=C[C@@H]2[C@@H](CC1)C(C)=CC[C@H]2C(C)C	-4.09
Magnolia officinalis	C=C(C)[C@@H]1CC=C(C)[C@@H](O)C1	-4.09
Magnolia officinalis	CC(C)=CCCC(C)C=O	-4.09
Magnolia officinalis	CC=CC1C2CCC(=O)C12	-4.09
Melissa officinalis	CCCC(=O)CCCC(=O)O	-4.08
Humulus lupulus	CCCCCC(=O)O	-4.08
Magnolia officinalis	CCCCCCCCCCCCCCCCCCCCCC	-4.08
Valeriana officinalis	C=C(C)CCOP(=O)(O)OP(=O)(O)O	-4.07
Valeriana officinalis	C=C1CC/C=C(C)CC[C@@H]2[C@@H]1CC2(C)C	-4.07
Melissa officinalis	C=CC(O)CCCCC	-4.07
Magnolia officinalis	CC1=CCC(CC=O)C1(C)C	-4.07
Magnolia officinalis	C=C1CC[C@@H]2[C@H]([C@@H]3[C@H](C)CC[C@@H]13)C2(C)C	-4.07
Valeriana officinalis	CC(=O)OCCC(C)CCC=C(C)C	-4.06
Humulus lupulus	CCCC(=O)O	-4.06
Valeriana officinalis	C=C(C)[C@@H]1CCC(C)=C[C@H]1O	-4.05
Scutellaria baicalensis	NCCc1c[nH]c2ccccc12	-4.05
Humulus lupulus	C=C1CC[C@H](C(C)C)[C@H]2C=C(C)CC[C@H]12	-4.05
Magnolia officinalis	C=CC1(C)CCCC(=C)CC1C=C(C)C	-4.05
Valeriana officinalis	C/C1=CCC/C(C)=C/CC(C)(C)/C=C/C1	-4.04
Magnolia officinalis	CCCCCCC/C=C/CCCCCCCC	-4.04
Valeriana officinalis	C=C1CC[C@H](C(C)C)[C@H]2C=C(C)CC[C@H]12	-4.03
Magnolia officinalis	C=CCCCCCCC=CCCCCCCCC=O	-4.03
Valeriana officinalis	CC1(C)CCC[C@@]2(C)CC[C@](C)(O)CC=C12	-4.02
Humulus lupulus	CCCCCCCCCCCCCCCCCCCCC(C)C	-4.02
Humulus lupulus	CC(C)CCOC(=O)C(C)C	-4.02
Humulus lupulus	CC(C)=CCOP(=O)(O)OP(=O)(O)O	-4.02
Humulus lupulus	C[C@@H]1CC[C@@H]2[C@H](C3=C1CC[C@H]3C)C2(C)C	-4.02
Humulus lupulus	C=C(C)[C@H]1C=C2[C@H](C)CC[C@@H]2[C@H](C)CC1	-4.02
Magnolia officinalis	CC(C)=CCCC(C)(O)C1CO1	-4.02
Valeriana officinalis	C=C[C@@]1(C)CCC(C(C)C)=C[C@@H]1C(=C)C	-4.01
Valeriana officinalis	CC1=C2C[C@H](C(C)(C)O)CC[C@]2(C)CCC1	-4.01
Humulus lupulus	CCC(C)CCC(=O)OC	-4.01
Humulus lupulus	CC(=O)CC(C)(C)S	-4.01
Magnolia officinalis	CCCCCC=CCC=CCCCCCCCCOCCO	-4.01
Valeriana officinalis	CC1=CCC2(C(C)C)CC12	-4
Humulus lupulus	C=C1CCC[C@]2(C)CCC(=C(C)C)C[C@@H]12	-4
Valeriana officinalis	C=C1CC[C@@H](C(C)C)[C@@H]2C=C(C)CC[C@H]12	-3.99
Humulus lupulus	Cc1ccc2c(c1)C(C(C)C)=CCC2C	-3.99
Humulus lupulus	CCCCCCCOC(=O)C(C)C	-3.99
Humulus lupulus	CCCCCCCCCCCCCCCCCCCC(C)CC	-3.99
Valeriana officinalis	CCCCCCCCCCCCCCCCOC(=O)CC(C)C	-3.98
Magnolia officinalis	CCCCCc1ccco1	-3.98
Humulus lupulus	CCCCCCCCC(CCC)CCCC	-3.97
Humulus lupulus	CCCCCCCCCC(C)O	-3.97
Humulus lupulus	CCCCCCCCCCCC(C)=O	-3.97
Humulus lupulus	CCCCCCCCCCCCCC(C)=O	-3.97
Humulus lupulus	CCC(=O)OCC(C)CC	-3.96
Humulus lupulus	CCCCC(C)CCCC(C)CCC(C)CC	-3.96
Passiflora incarnata	Cc1nccc2c1[nH]c1ccccc12	-3.95
Humulus lupulus	CC(=O)CC(C)(C)SCC(N)C(=O)O	-3.95
Humulus lupulus	COC(=O)CCCC(C)C	-3.94
Humulus lupulus	CCCC(S)CCO	-3.94
Humulus lupulus	C/C1=CCC[C@@]2(C)O[C@@H]2CC(C)(C)/C=C/C1	-3.93
Humulus lupulus	CC[C@H](C)[C@H](N)C(=O)O	-3.93
Magnolia officinalis	CC1=CCC=C(C)CCC(C)(C)C=CC1	-3.93
Melissa officinalis	CCCC(=O)CCCC=O	-3.92
Humulus lupulus	CCCOC(=O)C(C)CC	-3.91
Humulus lupulus	CCOC(=O)CC(C)C	-3.91
Humulus lupulus	CC1=C[C@@H]2C(=C(C)CC[C@H]2C(C)C)CC1	-3.91
Humulus lupulus	O=CC(O)COP(=O)(O)O	-3.91
Melissa officinalis	C=C(C)[C@@H]1CC=C(C)[C@@H](O)C1	-3.9
Valeriana officinalis	CC1=CCC2C(C1)C2(C)C	-3.89
Humulus lupulus	CCCCCCCCCCCCC(C)C	-3.87
Magnolia officinalis	CCCCCC(C)(CC)CCCC	-3.87
Humulus lupulus	CC(C)[C@H](N)C(=O)O	-3.86
Magnolia officinalis	CCCCCCCCCCCCCCCCCCCCCCCC	-3.85
Humulus lupulus	COC(=O)CCCCC(C)C	-3.83
Humulus lupulus	CCCCCCC(CO)CCCC	-3.82
Humulus lupulus	C=CCCCCCCC(=O)OC	-3.81
Humulus lupulus	CCC(C)C(=O)O	-3.81
Humulus lupulus	CCCCCCCCCCC(C)=O	-3.81

Magnolia officinalis	C=C1CC[C@H]2C[C@@H]1C2(C)C	-3.81
Humulus lupulus	CCOC(=O)C(C)CC	-3.8
Humulus lupulus	CC(=O)CC(C)C	-3.79
Humulus lupulus	CCCCCCCCCCCO	-3.79
Magnolia officinalis	CCCCCCCCCCCCCCC=O	-3.79
Magnolia officinalis	CC12CCC(C1)C(C)(C)C2O	-3.79
Magnolia officinalis	CCCCCCCCCCCCCCCCCCCCCCC	-3.78
Magnolia officinalis	C=C(C)C1CC=C(CO)CC1	-3.78
Valeriana officinalis	C=C[C@]1(C)CC[C@@H](C(=C)C)C[C@H]1C(=C)C	-3.77
Magnolia officinalis	CC1(C)C2CCC(C=O)C1C2	-3.77
Humulus lupulus	C=C(C)C=O	-3.76
Humulus lupulus	CCCCCCC(=O)OCC	-3.76
Humulus lupulus	CCC(S)CCO	-3.75
Magnolia officinalis	CCCCCCCO	-3.75
Magnolia officinalis	CC1(C)C2CC=C(CO)C1C2	-3.75
Magnolia officinalis	CCCCCCCCCCCCCCCCCOC(=O)C(Cl)Cl	-3.75
Humulus lupulus	C=C/C(C)=C/CC=C(C)C	-3.74
Magnolia officinalis	C=C1C2CCC(C2)C1(C)C	-3.74
Magnolia officinalis	CC(C)C12CCC(C)(CC1)O2	-3.74
Valeriana officinalis	C=C1CCC2(C(C)C)CC12	-3.73
Valeriana officinalis	CC1(C)[C@@H]2CC[C@@]1(C)[C@H](O)C2	-3.73
Humulus lupulus	CCCCCCC(C)CC(C)(C)C	-3.73
Valeriana officinalis	C=C1C[C@H]2CC[C@@H]1C2(C)C	-3.72
Valeriana officinalis	Cc1cncc2c1CC[C@@H]2C	-3.71
Magnolia officinalis	C=C/C(C)=C/CC=C(C)C	-3.71
Humulus lupulus	CCCCCCC(=O)OC	-3.7
Valeriana officinalis	CC12CCC(CC1=O)C2(C)C	-3.69
Humulus lupulus	C=CC(C)(C)O	-3.69
Humulus lupulus	CCCCCCCCCC(C)=O	-3.69
Humulus lupulus	CC(C)CC(=O)C(=O)O	-3.68
Magnolia officinalis	CC1(C)[C@H]2CC[C@]1(C)[C@H](O)C2	-3.68
Magnolia officinalis	CC1CC(O)C2C(C1)C2(C)C	-3.68
Magnolia officinalis	CCCCCCCCCCCCCCCCCCCCC	-3.67
Magnolia officinalis	C=C1C(=O)CC2CC1C2(C)C	-3.67
Valeriana officinalis	CC1(C)C2CC=C(CO)C1C2	-3.66
Humulus lupulus	CCCCCCCCOC(=O)C(C)C	-3.66
Humulus lupulus	CCC(C)C(=O)OC	-3.66
Humulus lupulus	CCC/C=C/C=O	-3.66
Humulus lupulus	CCCCCCCCCCCCCCCCC(=O)OC	-3.66
Valeriana officinalis	CC1(C)C2CCC1(C)C(O)C2	-3.65
Humulus lupulus	CC1(C)C2CC=C(CO)C1C2	-3.64
Humulus lupulus	CCCCCCCC(C)=O	-3.64
Valeriana officinalis	C=C1CCC2CC1C2(C)C	-3.63
Humulus lupulus	C=C/C(C)=C/CC=C(C)C	-3.63
Valeriana officinalis	CCCCCCCCCCCCCCC	-3.62
Valeriana officinalis	C=C(C)[C@H]1CC=C(C)CC1	-3.61
Magnolia officinalis	C=C(C)[C@H]1CC=C(C)CC1	-3.61
Magnolia officinalis	C=C1C=CC2(C(C)C)CC12	-3.61
Magnolia officinalis	CCCCC=CCC=CCCCCCCCCC(=O)OC	-3.61
Magnolia officinalis	C=C1C(=O)C2CC1C(C)(C)C2	-3.61
Humulus lupulus	CCC(C)C(=O)OCCC(C)C	-3.6
Humulus lupulus	CC(C)CC(=O)O	-3.6
Magnolia officinalis	CCCCCCO	-3.6
Magnolia officinalis	CC1=CC[C@H](C(C)C)C=C1	-3.6
Magnolia officinalis	CC1(C)[C@H]2CC[C@]1(C)[C@@H](OC=O)C2	-3.6
Humulus lupulus	CC(C)C(C)C(C)(C)C	-3.58
Magnolia officinalis	CCCCCCCCCCCCCC	-3.58
Humulus lupulus	C=C(C)CCOP(=O)(O)OP(=O)(O)O	-3.57
Humulus lupulus	CC(C)C(=O)O	-3.57
Valeriana officinalis	CCCCCCCCCCCCCCCC(=O)OC	-3.56
Magnolia officinalis	CCCCCCCCCCCCCCCCC(=O)OC	-3.56
Humulus lupulus	CCCCC(C)=O	-3.54
Humulus lupulus	CCOC(=O)C(C)C	-3.54
Magnolia officinalis	CCCCCCCCCCCCCCCCCCCCC	-3.54
Valeriana officinalis	CC1=CCC2CC1C2(C)C	-3.53
Valeriana officinalis	CC=Cc1ccc(OC)cc1	-3.53
Humulus lupulus	CCCCCCCC/C=CCCCCCCCC=O	-3.53
Humulus lupulus	CCCCCCCCC(=O)OC	-3.53
Humulus lupulus	C=C1CCC2CC1C2(C)C	-3.53
Humulus lupulus	CC1=CCC2CC1C2(C)C	-3.53
Melissa officinalis	CC(C)=CCC/C(C)=C/C=O	-3.52
Humulus lupulus	CCCCCCCCCCC(=O)OC	-3.52
Humulus lupulus	CC(=O)O	-3.52
Magnolia officinalis	C=C1CCC2CC1C2(C)C	-3.52
Valeriana officinalis	COc1cc(C)ccc1C(C)C	-3.51
Magnolia officinalis	CC1=C[C@@H](O)[C@H](C(C)C)CC1	-3.51
Humulus lupulus	CCCCCCCCCCCCCC	-3.5
Humulus lupulus	CCCCCCCCC=O	-3.5
Humulus lupulus	CCCCCCCCCCCC	-3.48
Magnolia officinalis	CC1=CCC2CC1C2(C)C	-3.48
Humulus lupulus	CCCCCC=CCCC(=O)OC	-3.46
Humulus lupulus	CCCCCCCCCCCCC	-3.45
Magnolia officinalis	CC=CC#CCCCC	-3.43
Humulus lupulus	CCCCCCCCC(C)=O	-3.42
Valeriana officinalis	CC(C)CC(=O)O	-3.41
Valeriana officinalis	CC1=CC=C(C(C)C)CC1	-3.36
Magnolia officinalis	CC1=CCC(=C(C)C)CC1	-3.35
Humulus lupulus	CC(C)=CC=O	-3.31
Melissa officinalis	CCCC1=NCCCC1	-3.3
Humulus lupulus	CCCCC=CCCC(=O)OC	-3.28
Valeriana officinalis	CCCCCC=O	-3.27

Magnolia officinalis	CC1=CC=C(C(C)C)CC1	-3.25
Valeriana officinalis	CC1=CCC(=C(C)C)CC1	-3.24
Humulus lupulus	CCCCCC=O	-3.23
Magnolia officinalis	CC1=CCC(C(C)C)=CC1	-3.22
Magnolia officinalis	CCCCCCCCC=O	-3.19
Magnolia officinalis	CC1=CCC2C(C1)C2(C)C	-3.17
Humulus lupulus	CCCCCOC(=O)C(C)C	-3.14
Humulus lupulus	CCCCCCCC(=O)OC	-3.13
Humulus lupulus	CCCCC/C=C/C=O	-3.13
Humulus lupulus	CCC=O	-3.09
Valeriana officinalis	CC1=CCC(C(C)C)=CC1	-3.07
Melissa officinalis	CCC1CCC(CC)O1	-3.07
Humulus lupulus	CCCCCCCCCC(=O)OC	-3
Humulus lupulus	CC(C)=O	-2.98
Humulus lupulus	CCC(C)=O	-2.96
Humulus lupulus	CCO	-2.89
Humulus lupulus	CC=C(C)CO	-2.71
Humulus lupulus	CSSSC	-2.06
Humulus lupulus	CC1=CC[C@@]23[C@@H]([C@@H]12)[C@H](C(C)C)CC[C@H]3C	0
Magnolia officinalis	c1(c(cc2c(c1)[C@@H]([N](CC2)(C)C)Cc1cc(c(cc1)OC)O)OC)O	0
Magnolia officinalis	C[C@H]1[C@H]2[C@H](C[C@H]3[C@@H]4C[C@@H](O)[C@H]5C[C@@H](O[C@@H]6O[C@H](CO)[C@H](O[C@@H]7O[C@H](CO)[C@@H](O)[C@H](O)[C@H]7O)[C@H](O)[C@H]6O)[C@H](O)C[C@]5(C)[C@H]4CC[C@]23C)O[C@]12CC[C@H](CO)CO2	0
Humulus lupulus	C=Cc1c(C)c2n3c1/C=C1N=C(/C=c4/c(C)c5c(n4[Mg]3)=C(C3=N/C(=C□)[C@@H](C)[C@@H]3CCC(=O)OC/C=C(C)CCCC(C)CCCC(C)CCCC(C)C)C(C(=O)OC)C5=O)C(CC)=C1C=O	0
